# Plant hormones and neurotransmitter interactions mediate antioxidant defenses under induced oxidative stress in plants

**DOI:** 10.3389/fpls.2022.961872

**Published:** 2022-09-09

**Authors:** Ali Raza, Hajar Salehi, Md Atikur Rahman, Zainab Zahid, Maryam Madadkar Haghjou, Shiva Najafi-Kakavand, Sidra Charagh, Hany S. Osman, Mohammed Albaqami, Yuhui Zhuang, Kadambot H. M. Siddique, Weijian Zhuang

**Affiliations:** ^1^Key Laboratory of Ministry of Education for Genetics, Breeding and Multiple Utilization of Crops, Oil Crops Research Institute, Center of Legume Crop Genetics and Systems Biology/College of Agriculture, Fujian Agriculture and Forestry University, Fuzhou, China; ^2^Laboratory of Plant Cell Biology, Department of Biology, Bu-Ali Sina University, Hamedan, Iran; ^3^Grassland and Forage Division, National Institute of Animal Science, Rural Development Administration, Cheonan, South Korea; ^4^Institute of Environmental Sciences and Engineering, School of Civil and Environmental Engineering, National University of Sciences and Technology, Islamabad, Pakistan; ^5^Department of Biology, Plant Physiology, Faculty of Science, Lorestan University, Khorramabad, Iran; ^6^Pharmaceutical Sciences Research Center, Health Institute, Kermanshah University of Medical Sciences, Kermanshah, Iran; ^7^State Key Laboratory of Rice Biology, China National Rice Research Institute, Chinese Academy of Agricultural Sciences, Hangzhou, China; ^8^Department of Agricultural Botany, Faculty of Agriculture, Ain Shams University, Cairo, Egypt; ^9^Department of Biology, Faculty of Applied Science, Umm Al-Qura University, Makkah, Saudi Arabia; ^10^College of Life Sciences, Fujian Agriculture and Forestry University, Fuzhou, China; ^11^The UWA Institute of Agriculture, The University of Western Australia, Perth, WA, Australia

**Keywords:** abiotic stress, climate change, drought stress, GABA, genetic engineering, melatonin, transgenic approach

## Abstract

Due to global climate change, abiotic stresses are affecting plant growth, productivity, and the quality of cultivated crops. Stressful conditions disrupt physiological activities and suppress defensive mechanisms, resulting in stress-sensitive plants. Consequently, plants implement various endogenous strategies, including plant hormone biosynthesis (e.g., abscisic acid, jasmonic acid, salicylic acid, brassinosteroids, indole-3-acetic acid, cytokinins, ethylene, gibberellic acid, and strigolactones) to withstand stress conditions. Combined or single abiotic stress disrupts the normal transportation of solutes, causes electron leakage, and triggers reactive oxygen species (ROS) production, creating oxidative stress in plants. Several enzymatic and non-enzymatic defense systems marshal a plant’s antioxidant defenses. While stress responses and the protective role of the antioxidant defense system have been well-documented in recent investigations, the interrelationships among plant hormones, plant neurotransmitters (NTs, such as serotonin, melatonin, dopamine, acetylcholine, and γ-aminobutyric acid), and antioxidant defenses are not well explained. Thus, this review discusses recent advances in plant hormones, transgenic and metabolic developments, and the potential interaction of plant hormones with NTs in plant stress response and tolerance mechanisms. Furthermore, we discuss current challenges and future directions (transgenic breeding and genome editing) for metabolic improvement in plants using modern molecular tools. The interaction of plant hormones and NTs involved in regulating antioxidant defense systems, molecular hormone networks, and abiotic-induced oxidative stress tolerance in plants are also discussed.

## Introduction

The rapidly growing global population necessitates a significant upsurge in agricultural production. Nevertheless, climate change-induced environmental factors significantly impact crop production worldwide (Raza et al., 2019a; Bahar et al., 2020; Fahad et al., 2021a). Climate variations impact the earth and agriculture through alterations in annual rainfall, mean temperature, heat waves, mutations in weeds, pests, and microbes, atmospheric ozone or carbon dioxide levels, and sea levels. The risk of climate change has significantly increased research interest, as these changes will adversely impact crop production and food security globally (Raza et al., 2019a; Bahar et al., 2020; Fahad et al., 2021b; Farooq et al., 2022; Haider et al., 2022). Specifically, to achieve the sustainable development goal proposed by the FAO of ‘zero hunger’ (SDG2) for an extra 2.3 billion individuals by the end of 2050, agricultural outputs must increase by 70% (Bahar et al., 2020)^1^. SDG2 is a multidimensional goal that extends beyond food security and requires comprehensive investigation^2^. Due to their sessile lifestyle, plants face numerous abiotic stresses, including salinity, drought, waterlogging, heavy metals, and temperature, that hinder crop production and threaten the world’s ecosystems (Raza et al., 2019a, 2020, 2021a, b, 2022a, b, c; Hasanuzzaman et al., 2020a; Mir et al., 2022). These stresses are the main factors affecting plant physiological, biochemical, and cellular mechanisms, as they modulate mixed responses that attempt to avert damage and promote plant persistence under adverse stress conditions. Ultimately, stress-induced cellular, biochemical and molecular modifications improve plant growth and development (Ahanger et al., 2017; Nguyen et al., 2018; Hasan et al., 2021; Raza, 2021, 2022a,b).

Identifying plant response mechanisms to various abiotic stresses is crucial for plant biotechnologists. However, stress tolerance characteristics are challenging to incorporate into plants using conventional breeding methods, and current breeding and molecular tools are inadequate to feed the growing world population. Accordingly, novel and powerful techniques (such as foliar treatment or application of diverse biotechnological tools) are needed to improve crop stress tolerance. In the era of climate change, it is crucial to recognize the endogenous mechanisms regulating the various processes and mechanisms responsible for abiotic stress tolerance (Tanveer and Yousaf, 2020; Fahad et al., 2021c; Farooq et al., 2021; Sharma et al., 2021, 2022a). Furthermore, improving plant growth rates and adaptation to environmental stimuli by altering hormonal signals in cells and tissues is important. For example, enabling plants to exhibit resistance/tolerance to environmental stimuli without decreasing growth is critical for meeting the world’s exponentially growing food demand, especially under environmentally challenging conditions (FAO, 2017).

**FIGURE 1:**
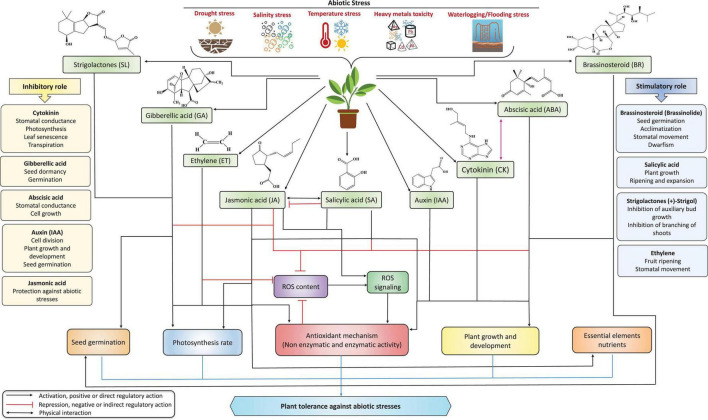
The role of phytohormones in improving plant tolerance against multiple abiotic stresses. Under stress conditions, phytohormones can modulate the stress intensity in plants by triggering defense mechanisms and thus regulate physio-biochemical processes by increasing plant tolerance to environmental stress. CK, GA, ABA, IAA, and JA mainly play inhibitory roles, whereas BRs, SA, SLs, and ethylene play stimulatory roles in improving several physiological and biochemical mechanisms under stress conditions. Notably, ABA is a primary driving force, playing a vital role alone or combined with other hormones under stress. Furthermore, CKs and auxin play a dual role (inhibitory and stimulatory) by regulating plant growth and development processes.

Exogenous application or/and genes responsible for the biosynthesis of plant hormones or phytohormones, such as abscisic acid (ABA), jasmonic acid (JA), salicylic acid (SA), brassinosteroids (BRs), indole-3-acetic acid (IAA), cytokinins (CKs), ethylene (ET), gibberellic acid (GA), and strigolactones (SLs), could be used to develop climate-resilient crops with improved yields under stressful conditions (Figure 1; Cooper et al., 2018; Ku et al., 2018; Raza et al., 2019b; Yao et al., 2020; Mubarik et al., 2021; Sabagh et al., 2021; Waadt et al., 2022). As plant growth regulators, plant hormones are produced in minimal amounts but can regulate diverse plant cellular processes. They act as chemical envoys to interconnect cellular actions and play vital roles in harmonizing several signal transduction pathways through abiotic stress responses and adjusting exterior and interior responses to stimuli (Voß et al., 2014; Ku et al., 2018; Sabagh et al., 2021). Neurotransmitters (NTs) are a group of neuroregulatory molecules produced by mammals and plants (Tanveer and Shabala, 2020) that play a pivotal role in organogenesis, flowering, photosynthesis, reproduction, and plant adaptation to environmental factors (Akula and Mukherjee, 2020). Recent studies found evidence of plant hormones and NTs involved in plant development, stress adaptation, and stress tolerance (Arnao and Hernández-Ruiz, 2017, 2021). The interaction of NTs with plant hormones regulates plant antioxidant systems and stress indicators, reducing stress-induced oxidative injuries (Fu et al., 2017; Yang et al., 2019). Moreover, NTs are potent elements that interact with plant growth regulators in response to various environmental stimuli, increasing stress tolerance.

Abiotic stress factors generate reactive oxygen species (ROS), causing oxidative stress in most plants (Hasanuzzaman et al., 2020a). To combat the effects of stress, plants have evolved various enzymatic and non-enzymatic antioxidants to scavenge ROS, known as redox regulatory mechanisms (Hasanuzzaman et al., 2020a; Tiniola et al., 2021). Oxidative stress in plants is mainly counteracted through endogenous self-protective machinery comprising antioxidant defense mechanisms—both enzymatic [superoxide dismutase (SOD), catalase (CAT), ascorbate peroxidase (APX), glutathione reductase (GR), monodehydroascorbate reductase (MDHAR), dehydroascorbate reductase (DHAR), glutathione peroxidase (GPX), guaiacol peroxidase (GOPX), and glutathione *S*-transferase GST)] and non-enzymatic [ascorbic acid (AsA), glutathione (GSH), phenolic acids, alkaloids, flavonoids, carotenoids, alpha-tocopherol, and non-protein amino acids] (Hasanuzzaman et al., 2020a). Ideally, the antioxidant defense system and ROS accumulation in plant cells maintain a steady-state equilibrium to maintain appropriate redox biology responses which regulate diverse processes necessary for plant growth and development (Mittler, 2017; Hasanuzzaman et al., 2020a; Mittler et al., 2022). However, under stress, ROS overproduction annihilates this balance, generating cellular injury, programmed cell death (PCD), and enhanced ROS production (Raja et al., 2017; Mittler et al., 2022). Importantly, in addition to modulating numerous mechanisms and producing compounds to enhance abiotic stress tolerance, plant hormones enhance the activity of antioxidant defense systems.

The ROS-scavenging effects of antioxidants depend on the tissue and organelle types. The interplay between ROS, antioxidants, and plant hormones and the changes in metabolic networks determine plant survival in stressful environments. Consequently, several approaches combined with exogenous phytohormone supplementation are used to modulate gene expression and signaling pathways to improve the ability of plants to cope with stressful conditions (Riyazuddin et al., 2019; Srivastava et al., 2019; Jiang et al., 2020; Saxena et al., 2020). This review explores current knowledge on the role of plant hormones in crop improvement under normal and stressful conditions. Further, we also reviewed the plant metabolic advancement using genetic engineering tools like gene overexpression and editing via clustered regularly interspaced short palindromic repeats-CRISPR-associated proteins (CRISPR/Cas) system. Moreover, this review also explores the plant hormones and NTs interactions to enhance abiotic-induced oxidative stress tolerance by regulating antioxidant potential.

## Abiotic stresses: A challenge to the agricultural system

As abiotic stresses cause extensive crop losses worldwide, exploring how they disturb plant growth and development at the physiological, biochemical, and molecular levels is vital for improving plant productivity. Climate changes lead to major abiotic stresses such as temperature (high/low), drought, salinity, toxic metals, waterlogging, and nutrient imbalance, which significantly hinder crop productivity (Raza et al., 2019a, 2020, 2021a, 2022a, b, c; Fahad et al., 2021a,b; Raza, 2021; Farooq et al., 2022). Stress duration, stress progress, and the plant growth phase are the key factors that affect plant stress responses (Feller and Vaseva, 2014; Sharma et al., 2021).

Vulnerability or tolerance to a given stressor varies between crop species and genotypes. Drought is possibly the most significant abiotic stress, significantly reducing agricultural production worldwide. Drought retards plant growth by altering membrane stability, pigmentation, osmotic balance, water balance, and photosynthetic activity (Suzuki et al., 2014; Sallam et al., 2019; Gondal et al., 2021; Wasaya et al., 2021). Salinity is the next most prominent abiotic stress impairing crop productivity (Isayenkov and Maathuis, 2019; Alamri et al., 2020; Raza et al., 2022c), followed by various other environmental stresses. Soil salinity comprises saline, alkaline, and saline–alkaline soils categorized as increased salt intensity, pH, and sodium level, respectively (Isayenkov and Maathuis, 2019). Likewise, low and high-temperature stresses lead to cell dehydration, cell starvation, and the breakdown of plant proteins, causing cell wall lysis (Suzuki et al., 2014; He et al., 2021; Raza et al., 2021a,b). The upsurge in macro- and micro-nutrients due to alterations in nutrient uptake, transport, assimilation, and their (macro- and micro-nutrients) biological interactions also negatively affects plant productivity (Salim and Raza, 2020; Amanullah et al., 2021; Bukhari et al., 2021; Makawita et al., 2021). Another factor is the presence of metals/metalloids in the environment due to physical and anthropogenic actions, disturbing basic physiological and biochemical processes in plants (Hasanuzzaman et al., 2020b,c; Raza et al., 2021d, 2022a). Under submerged and waterlogged conditions, oxygen concentration—normoxia, hypoxia, or anoxia—significantly affects plant growth and production (Zahra et al., 2021). Additionally, exposure to light and UV radiation, pH changes, gaseous contaminants, and several mechanical factors have been associated with plant stress (Suzuki et al., 2014).

The detrimental effects of most abiotic stresses hinge on ROS overproduction at some point. ROS molecules oxidize and degrade carbohydrates, lipids, nucleic acids, and proteins in plant cells (Hasanuzzaman et al., 2020a); numerous studies have revealed that plant hormones have positive, protective roles against these stresses. In addition, several interventions have successfully leveraged plant hormones in promoting plant tolerance to an individual or combined stressors.

## Oxidative stress: A combined effect

**FIGURE 2:**
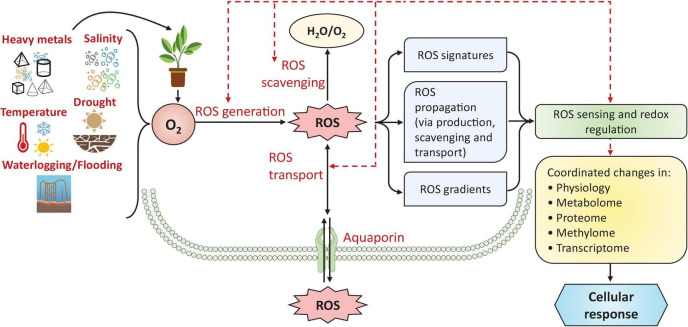
Management of ROS metabolism and signaling in plants under stress conditions. Cellular ROS accumulation is controlled by three main methods—(1) ROS generation, (2) ROS scavenging, and (3) ROS transport—which maintain ROS concentrations and produce various ROS signatures and gradients that act as signals in various abiotic factor-response signal transduction pathways. These redox regulations lead to coordinated changes in the plant’s physiology, metabolome, proteome, methylome, and transcriptome. Dashed arrows show that ROS generation, scavenging, and transport can be controlled by the ‘redox state’ of plant cells under stress. Figure based on the concept of Mittler et al. (2004, 2022). For more information on ROS metabolism and signaling, readers are referred to Mittler et al. (2022). ROS, reactive oxygen species; O_2_, oxygen; H_2_O, water.

The abiotic-stress-induced disturbance of numerous metabolic functions and physiological processes leads to ROS overproduction (Choudhury et al., 2017). The damaging properties of free oxygen radicals are called ‘oxidative stress.’ Redox reactions are common in living creatures and account for most ROS generation (Decros et al., 2019). In plant cells, redox homeostasis is the normal condition where a competent defense system maintains an appropriate balance between ROS production and eradication (i.e., antioxidant activity) (Paciolla et al., 2016). A basal amount of ROS is crucial for appropriate redox signaling (Mittler, 2017; Mittler et al., 2022); the term ‘redox biology’ refers to the role of ROS as signaling compounds that regulate and sustain typical physiological events of plants (Schieber and Chandel, 2014; Kumar et al., 2016; Mittler, 2017; Mittler et al., 2022). Low levels of ROS trigger signaling that alters regular plant metabolism, while excess ROS initiates oxidative cellular impairment (Mittler et al., 2004; Decros et al., 2019). Consequently, biomolecular mechanisms that ensure stable equilibrium among ROS production and scavenging are powerfully harmonized, functioning with the cellular redox-sensitive apparatus to generate and regulate downstream signaling events in a cell-precise and context-precise manner (Panieri and Santoro, 2015; Mittler et al., 2022). During the stress conditions, ROS accumulation in plant cells disturbs the ‘redox state’ of various proteins, such as enzymes, receptors, and tiny molecules, triggering, altering, or participating in various abiotic factor-response signal transduction pathways (Figure 2; Mittler et al., 2004, 2022).

There is a growing perception that the roles of the enzymes and metabolites of redox metabolism extend far beyond the simple ROS-scavenging function. For one, as dedicated ROS signaling processors, these enzymes act as integral parts of a complex signaling system (Leister, 2019). The broader roles of ROS (mainly H_2_O_2_) under stress came into focus at the start of the 21st century. Several researchers documented H_2_O_2_ as a signaling molecule, promoting acclimation progression and enhancing tolerance to various environmental stresses (Khedia et al., 2019). In addition, ROS produced in a chloroplast under stress might divert electrons from the photosynthetic apparatus, preventing associated injury; ROS also similarly defend mitochondria (Choudhury et al., 2017). Concerning signaling, cell wall peroxidase (POD/POX) might subsidize ROS production, with H_2_O_2_ acting as a messenger upstream of calcium (Ca^2+^), protein phosphorylation, and mitogen-activated protein kinase (MAPK) pathways (Tanveer and Ahmed, 2020).

**FIGURE 3:**
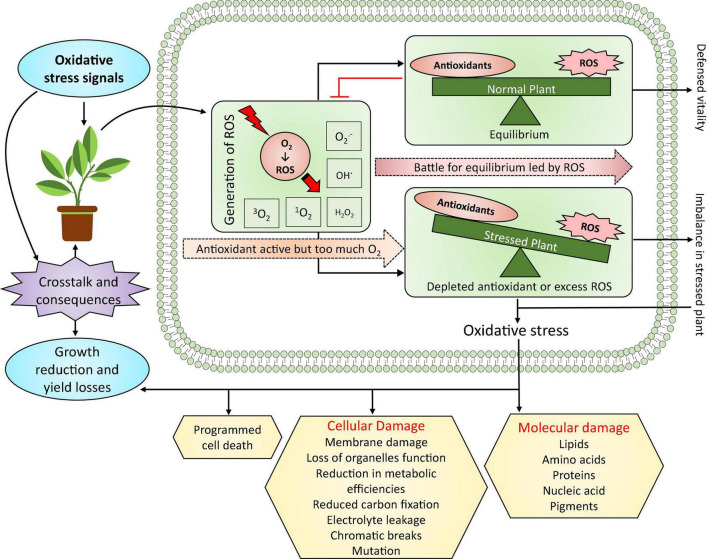
Oxidative stress in plants and its significance. Under abiotic-stress-induced oxidative stress, ROS generation is the most significant step, which leads to the battle for equilibrium between ROS and antioxidant defense. This involves substantial crosstalk and consequences between stress signals and plant growth and yield reduction. For instance, minor damage caused by oxidative stress can improve growth and yield, whereas extreme oxidative stress can significantly reduce plant growth and yield—modified from Hasanuzzaman et al. (2020a). ROS, reactive oxygen species; H_2_O_2_, hydrogen peroxide; O_2_, oxygen; ^1^O_2_, singlet oxygen; ^3^O_2_, triplet oxygen; ${\text{O}}_{2}^{∙-}$, superoxide; OH^∙^, hydroxyl radical.

Recent work elaborated on the crosstalk between ROS and plant hormones, particularly ET and ABA, in the context of stress responses and improving tolerance, confirming ROS’s double role under stress conditions (Medeiros et al., 2020; Postiglione and Muday, 2020). In addition, ROS can adjust plant metabolism under abiotic stress, activating redox responses that regulate the transcription and translation of stress acclimation proteins and enzymes, ultimately defending plant cells from injury (Choudhury et al., 2017; Mittler, 2017; Mittler et al., 2022). In addition, H_2_O_2_ modifies the NO and Ca^2+^ signaling pathways, controlling plant growth and development and other cellular and physiological responses (Niu and Liao, 2016; Singh et al., 2020). Notably, disrupting the balance between ROS production and ROS scavenging by the antioxidant defense system that leads to ROS overproduction causes follow-on oxidative damage. Such oxidative stress gives rise to molecular and cellular damage, leading to cell death (Figure 3; Hasanuzzaman et al., 2020a).

## Interplay of plant hormones in conferring abiotic-induced oxidative stress tolerance in plants

**FIGURE 4:**
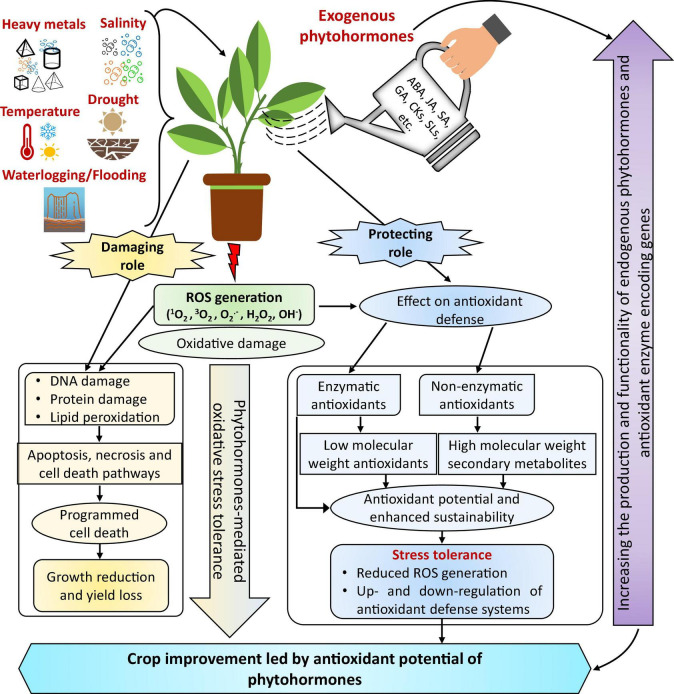
The proposed model demonstrates the state-of-the-art potential of a phytohormone-mediated antioxidant defense system under abiotic stress. Notably, phytohormones decrease the damaging effect of oxidative stress induced by abiotic stress because they act as secondary messengers to initiate antioxidants and can thus scavenge ROS in stressed plants. Interestingly, exogenous phytohormone application can reduce ROS overproduction, enhancing the activities of several antioxidant defense systems and stress tolerance; it varies with the type of stress, plant type, and duration. However, ROS overproduction can significantly reduce growth and yield under stressful conditions. In addition, exogenous phytohormones can increase the production and functionality of endogenous hormones and transcript levels of antioxidant enzyme-encoding genes, e.g., *SOD, CAT*, and *POD*. The optimal dose and growth stage for phytohormone application needs further investigation—modified from Raza et al. (2021c) with permission from the publisher (Springer Nature).

Plant hormones are a driving force for modulating abiotic stress responses, plant growth, and developmental mechanisms (Figure 1; Wani et al., 2016; Ku et al., 2018; Raza et al., 2019b; Mubarik et al., 2021; Sabagh et al., 2021; Waadt et al., 2022). Accordingly, managing endogenous phytohormone levels with exogenous supplementation and modern biotechnological techniques can help engineer plant metabolism and improve plant stress tolerance (Wani et al., 2016). Plant hormone mechanisms of action, including oxidative stress and the management of ROS production, stimulate divergent mechanisms *in vitro* (Szechyńska-Hebda et al., 2007; Wani et al., 2016; Rehman et al., 2021; Al-Zahrani et al., 2022; Waadt et al., 2022). Concerning ROS, plant hormones improve the activities of enzymatic and non-enzymatic antioxidant defense systems (Figure 4). Several plant hormones control biochemical and physiological mechanisms under stressful conditions. Thus, there is a growing interest in leveraging plant hormones to enhance the activity of antioxidant systems and improve plant tolerance to oxidative stress induced by abiotic factors (Table 1).

**TABLE 1:** Application of some major plant hormones to plants under stress and their impact on antioxidant systems. Notably, all presented studies increased stress tolerance and improved plant health. Abbreviations are explained in the text.

Stress type	Stress condition	Concentration	Species	Key findings and impact on antioxidant systems	References
**Abscisic acid (ABA)**					
Drought and heat	PEG solution (−0.7 MPa), 8 h, and 28–42°C with an interval of 2°C h^–1^	100 μM	Maize (*Zea mays* L.)	ABA-induced antioxidant defense system increased stress tolerance, and SOD, CAT, APX, and GR activities increased in leaves and roots	Hu et al., 2010
Chilling	10°/6°C (day/night); 1, 3, 6, 12, 24, 48, 72, and 120 h	0.57 mM	Chili pepper (*Capsicum annuum* L.)	ABA increased stress tolerance by increasing SOD and guaiacol peroxidase activities	Guo et al., 2012
Drought	15% PEG; 1, 2, 3, 4, and 5 days	10 μM	Wheat (*Triticum aestivum* L.)	ABA enhanced drought tolerance by regulating GSH-AsA encoding genes and increasing GSH and AsA activities in leaves and roots	Liting et al., 2015
Drought and salinity	5, 10, and 15% PEG; 10 days, and 0.87 and 1% NaCl, 7 days	50 μM	Cotton (*Gossypium hirsutum* L.)	ABA application increased stress tolerance and improved SOD and CAT activities and proline level	Liang et al., 2016
Cold, salinity, osmotic and UV-B radiation, respectively	4°C, 1, 3, 6, 12, or 24 h; 200 mM NaCl; 20% PEG and 8 W UV-B lamp, 0.5, 1, 3, 6, or 12 h, respectively	50 μM	Arabidopsis (*Arabidopsis thaliana* L.)	Exogenous ABA increased tolerance to salt and oxidative stresses and improved SOD and CAT activities under stress	Li et al., 2017a
Heavy metals	Total and available Cd 8.46 and 2.12 mg kg^–1^; total and available Pb 753 and 230 mg kg^–1^	0, 20, 40, and 60 μmol L^–1^	*H. spectabile*, *S. alfredii*	ABA application increased total Chl content and antioxidant capacity and significantly alleviated metal-induced growth inhibition	Cheng et al., 2022
Salinity	200 mM NaCl, 72 h	1 mM SNP and 10 μM ABA	Rice (*Oryza sativa* L.)	ABA improved stress tolerance and was involved in the recovery of antioxidation pathways, osmotic tolerance, and carbohydrate metabolism	Saha et al., 2022
**Jasmonic acid (JA)**					
Salinity	150 mM NaCl; 3 days	2 mM	Wheat (*Triticum aestivum* L.)	Exogenous supply of JA increased stress tolerance by reducing MDA and H_2_O_2_ levels and improving CAT, SOD, POD, and APX activities, and GSH, Chl b, and carotenoid concentrations	Qiu et al., 2014
Drought	15% PEG, 48 h	0.5 mM	*Brassica* spp.	JA treatment improved oxidative stress tolerance by increasing proline contents, AsA/DHA ratios, GSSG, GPX, GR, Gly I, DHAR activities. It also increased seedling fresh and dry weights	Alam et al., 2014
Nickel	2 mM Ni solution, 15 days	1 nM	Soybean (*Glycine max* L.)	JA supplementation improved osmolytes and increased the activities of SOD (40.04%), POD (28.22%), CAT (48.53%), and APX (56.79%)	Sirhindi et al., 2016
Salinity	0.67, 5, 10, and 15 dS m^–1^	0.5 mM	Barley (*Hordeum vulgare* L.)	JA treatment increased stress tolerance by improving the efficiency of SOD, CAT, POX, and K^+^/Na^+^	Pakar et al., 2016
Cold	5°C; 12, 24, and 48 h	150 μM	Apple (*Malus baccata* L.)	JA reduced oxidative injury and improved activities of AsA, DHA, APX, MDHAR, DHAR, GSH/GSSG ratio, and GR at different time points (12, 24, and 48 h)	Li et al., 2017b
Heavy metals	Total and available Cd 50 and 4.05 mg kg^–1^; total and available Pb 680 and 9.08 mg kg^–1^	0, 0.2, 0.4, and 0.8 mmol L^–1^	Quail bush (*Atriplex lentiformis* L.)	JA enhanced phytoextraction efficiency and plant growth, antioxidant enzyme activities (e.g., APX, PPO), phenolic compounds, and the synthesis of osmoregulatory compounds (e.g., soluble carbohydrates and proline)	Ibrahim et al., 2022
Heavy metals	50 μM Cd, 45 days	0.5 mM	Pea (*Pisum sativum* L.)	JA improved stress tolerance, enzymatic activities (e.g., NRA, NiRA, SOD, POD, and CAT), and Chl *a* and *b* and carotenoids	Abbas et al., 2022
Heavy metals	0, 100, 200, and 400 μM Ni	0, 5, and 10 μM	Sweet alyssum (*Alyssum inflatum*)	JA ameliorated root biomass and plant tolerance by restricting Ni translocation to shoots, accumulating in roots, and increasing antioxidant defense systems	Najafi-Kakavand et al., 2022
**Salicylic acid (SA)**					
Salinity	NaCl at 0, 100, 200, 300 and 400 mM L^–1^	1.0 mM L^–1^	Rice (*Oryza sativa* L.)	SA reduced the harmful effects of salinity by modulating the activities of antioxidant systems; SOD, CAT, and POX increased under salt stress and decreased with SA application	Jini and Joseph, 2017
Drought	70% FWC; 6 days	10 or 100 μM	Wheat (*Triticum aestivum* L.)	SA treatment improved stress tolerance, SOD, CAT, and GPX activities, and proline content	Kolupaev et al., 2018
Salinity	NaCl 50, 100, and 150 mM; 9 weeks	0.5 mM	Mustard (*Brassica carinata* L.)	Foliar SA supplementation reduced the salinity effect and improved the efficiency of SOD (29–32%), CAT (27–25%), and POX (179–194%)	Husen et al., 2018
Cold	4°C; 2 or 4 days	3 mM AsA	Common bean (*Phaseolus vulgaris* L.)	Exogenous AsA reduced the damaging effect of stress and improved APX, SOD, POD, and CAT activities	Soliman et al., 2018
Heat	42°C; 36 h	1 mM	Tomato (*Solanum lycopersicum* L.)	Increased activities of SOD (36.6%), POD (136.3%), CAT (250%), and APX (65.8%), decreasing oxidative injury	Jahan et al., 2019
Artificial magnetism	250 ppm, 20 days	250 μM SA	Pea (*Pisum sativum* L.)	SA improved various growth attributes, photosynthetic pigments (Chl *a, b*, and carotenoids), and soluble sugar contents	Naseer et al., 2022
Heavy metals	500 μM Cd, 15 days	0.25 μM	Milk thistle (*Silybum marianum* L.)	SA application improved growth attributes (root/shoot length, leaf area, and root/shoot fresh and dry weights), photosynthetic pigments (Chl *a*, *b*, and carotenoids), and secondary metabolites (anthocyanin, soluble phenolics, and tannins) and decreased oxidative parameters such as MDA and H_2_O_2_ contents	Nizar et al., 2022
Salinity	10, 35, and 70 mM NaCl	0, 0.25, 0.50, and 1 mM	Pomegranate (*Punica granatum* L.)	SA application improved chlorophyll, total phenolic, carbohydrate, and proline contents and POD and CAT activities and decreased MDA content, electrolyte leakage (EL), Na, and Cl levels	Khalil et al., 2022
Salinity	100, 200, and 300 mmol L^–1^ NaCl solution	0, 0.2, 0.4, 0.6, 0.8, and 1.0 mmol L^–1^	Soapwort (*Saponaria officinalis* L.)	SA improved salt tolerance capacity by modulating photosynthetic rate, osmoprotectants, antioxidant levels, and ion homeostasis	Xu et al., 2022c
**Brassinosteroids (BRs)**					
Salinity	NaCl 100 and 150 mM; 3 weeks	0, 0.5, 1.5, and 2.5 mg L^–1^	Peppermint (*Mentha piperita* L.)	BR-treated plants had improved stress tolerance and SOD, CAT, and APX activities	Çoban and Baydar, 2016
Water deficit	18–20 days after start of experiment	0, 50, and 100 nM EBR	Cowpea (*Vigna unguiculata* L.)	EBR reduced cell damage and increased stress tolerance by increasing the activities of SOD (25%), CAT (29%), APX (50%), and POX (149%) and PSII efficiency	Lima and Lobato, 2017
Cold	4°C; 3 days	0.1 μM	Tomato (*Solanum lycopersicum* L.)	Endogenous BRs increased chilling tolerance and APX, MDAR, DHAR, and GR activities	Xia et al., 2018
Cold	4°C; 1, 3, 6, 12, 24, 72, and 120 h	0, 0.01, 0.1, 1, 10, and 10 μM	*Elymus nutans* L.	BRs improved cold tolerance and enhanced CAT, APX, GR, DHAR, and MDAR activities	Fu et al., 2019
Drought	Suppressing 100% water needs at 30–37 and 73–80 days after transplanting	0, 1, 2, 4, or 8 ml	Naranjilla (*Solanum quitoense* Lam.)	Foliar DI-31 (BRs) sprays enhanced leaf photosynthesis and photosynthetic pigment concentrations and reduced MDA	Castañeda-Murillo et al., 2022
Heavy metals	400 μM Al	0.01 μM	Rice (*Oryza sativa* L.)	BRs alleviated Al injury by lowering MDA and H_2_O_2_ levels and increasing antioxidant activities and photosynthetic pigments	Basit et al., 2022b
**Indole-3-acetic acid (IAA)/auxin**					
Salinity and heavy metal	160 mM NaCl and 250 μM CdCl_2;_ 24–48 h in 7-day-old seedlings	100 μM IAA-Asp	Pea (*Pisum sativum* L.)	IAA improved stress tolerance by regulating APX, GPX, and POX activities; no effect on CAT activity	Ostrowski et al., 2016
Heavy metal	1, 3, 6, 9, 12, and 15 mg Cd kg^–1^ soil (after 15 days of growth)	10 μM and 100 μM	Fenugreek (*Trigonella foenum-graecum* L.)	IAA supplementation increased AsA-GSH cycle, reduced cell damage, and increased the activities of different antioxidant defense enzymes such as SOD, CAT, GSH, POD, GST, APX, DHA, DHAR, and GR	Bashri and Prasad, 2016
Salinity	3, 4, 5 and 6 dS m^–1^ NaCl at 3 days intervals	50 μM	Rice (*Oryza sativa* L.)	Increased activities of AsA, ASO, α-tocopherol under salt stress	Saedipour, 2016
Drought	Withholding water for 3 weeks	80 ppm	Wheat (*Triticum aestivum* L.)	IAA improved plant health under stress and increased activities of SOD (33%, 15.26%, and 38%) and POD (90%, 77%, and 82%)	Muhammad et al., 2016
Salinity	NaCl 120 mM; 2 weeks	12.5, 25, and 50 ppm	Sunflower (*Helianthus annuus* L.)	IAA improved stress tolerance and the activities of CAT (152.24% and 350.64%) and PPO (110.14% and 100.82%) under salinity	Zayed et al., 2017
Salinity	80 mM NaCl	2 mM IAA	Eggplant (*Solanum melongena* L.)	Foliar IAA sprays ameliorated the salinity effects on biomass production, biochemical, physiological, and yield attributes	Shahzad et al., 2022
Heavy metals	30 mg kg^–1^ Cd-spiked soil	10-mg kg^–1^ IAA	Perennial ryegrass (*Lolium perenne* L.)	IAA alleviated Cd stress and increased biomass, Chl content, SOD activity, sucrose activity, fluorescein diacetate (FDA) hydrolase activity, and Cd removal rate by 14.5%, 19.9%, 24.3%, 12.1%, 20.4%, and 15.1%, respectively	Xu et al., 2022b
**Cytokinins (CKs)**					
Drought	Water equivalent to 50% evapotranspiration (ET) during 1–19 days, 40% ET during 20–32 days, and 20–30% ET during 32–40 days	10 and 100 μM	Creeping bentgrass (*Agrostis stolonifera* L.)	Foliar supplementation of CKs improved stress tolerance and enhanced the activities of SOD (25%), APX (22%), CAT (17%, and POD (24%)	Chang et al., 2016
Heavy metal	100 μM	0.01, 0.1, and 1 μM 100 μM DPU Pb supplied on day 3, 5, and 7 of algal cultivation	*Acutodesmus obliquus* L.	CK treatment enhanced the activities of AsA (41%), GSH (76%), SOD (76%), CAT (41%), GR (110%), APX (43%), and proline (126%) under Pb toxicity and improved plant adaptation to Pb	Piotrowska-Niczyporuk et al., 2018
Drought and high temperature	20% PEG, and 37 ± 2°C; 10 days	10 ppm BAP	Wheat (*Triticum aestivum* L.)	Reduced the harmful impact of combined stress and improved CAT, APX, and proline activities	Kumari et al., 2018
Salinity	0, 75 or 150 mM NaCl	50 or 40 μM *t*-*Z*-Ck	Maize (*Zea mays* L.)	*t*-*Z*-Ck enhanced maize growth and productivity by suppressing the effects of oxidative stress caused by saline water irrigation	Azzam et al., 2022
Drought	40 and 120 mm evaporation	20 μM CK and 20 μM ABA	Wheat (*Triticum aestivum* L.)	Foliar-applied CK alleviated drought stress and increased K^+^, Ca^2+^, Mg^2+^, Fe^2+^, and Zn^2+^, but decreased Na^+^	Khosravi-nejad et al., 2022
**Gibberellins (GAs)**					
Salinity	100 mM NaCl; 10 days	0.1 mM	Okra (*Abelmoschus esculentus* L.)	Foliar application of GA_3_ reduced the adverse effects of NaCl stress, enhanced the activities of SOD, CAT, and POD, and reduced EL, MDA, and H_2_O_2_ contents	Wang et al., 2019
Salinity	100 mM NaCl every 4 to 20 days	1.4 μM	Tomato (*Solanum lycopersicum* L.)	GA_3_ decreased oxidative stress by increasing the activities of CAT (49.7), APX (45.4%), GR (152.0%), DHAR (39.2%), MDHAR (98.1%), and GPX (63.2%), enhancing Chl contents, and decreasing the over-generation of ROS and glycolate oxidase activity	Siddiqui et al., 2020
Cerium oxide nanoparticles (CeO_2_-NPs)	100, 200, 300, 400, and 600 mg kg^–1^	100 and 200 mg L^–1^	Wheat (*Triticum aestivum* L.)	Foliar spray of GA increased plant growth, Chl and nutrient contents, and yield, and decreased oxidative stress by increasing CAT, SOD, APX, and POD activities	Iftikhar et al., 2020
Heat	44°C; 30 days	100 μM	Date palm (*Phoenix dactylifera* L.)	GA_3_-mediated oxidative stress decreases with decreasing MDA and superoxide anions and increasing CAT, POD, APX, and polyphenol oxidase activities	Khan et al., 2020
Salinity	100 mM NaCl for 41 days	0.1 mM	Papaya (*Carica papaya* L.)	GA_3_ acted as a growth regulator and osmoregulatory solute and increased adaptation against salt stress by increasing stomatal conductance, plant biomass, and stem height	Álvarez-Méndez et al., 2022
Salinity	0 and 5 dS m^–1^ Na stress	10^–4^ M for 12 h	Pea (*Pisum sativum* L.)	GA_3_ alleviated salt stress and increased plant biomass and yield, Chl content, antioxidant enzyme activity, and soluble protein content, and reduced Na^+^ transport	Gurmani et al., 2022
**Strigolactones (SLs)**					
Salinity	100 and 200 mM NaCl; 7 days	0.18 μM GR24	Rapeseed (*Brassica napus* L.)	SLs increased canola growth and photosynthesis and reduced oxidative stress by modulating POD and SOD activities and decreasing MDA content	Ma et al., 2017
Drought	PEG-6000; 2, 12, 24, 72, 96, and 120 h	1, 3, and 5 μM GR24	Common grape vine (*Vitis vinifera* L.)	SLs improved drought tolerance by decreasing EL, ROS, MDA, and Chl contents and increasing SOD activity (105 and 90%)	Min et al., 2019
Drought	40% water holding capacity	10 and 20 μM	Maize (*Zea mays* L.)	SLs improved plant growth by improving gas exchange parameters, water relations, and Chl pigments	Sattar et al., 2022
Salinity	150 mM NaCl	15 μM	Tomato (*Solanum lycopersicum* L.)	SLs participated in NO-enhanced salinity tolerance in tomato seedlings by increasing photosynthetic pigment content, enhancing antioxidant capacity, and improving endogenous SLs synthesis	Liu et al., 2022
**Ethylene**					
Heavy metals	100 mg kg^–1^ soil Cu	200 μL L^–1^ ethephon	Mustard (*Brassica juncea* L.)	Ethylene mitigated the negative effect of Cu and decreased Cu accumulation, lowering lipid peroxidation, lignin accumulation, and ROS content	Rather et al., 2022

### Abscisic acid

Abscisic acid (ABA) is an isoprenoid phytohormone formed in the plastid 2-C methyl-D-erythritol-4-phosphate pathway. It is a well-studied phytohormone due to its action and diverse roles in plant adaptation to several abiotic stresses and, accordingly, termed a stress hormone (Trivedi et al., 2016). In the past two decades, ABA has been documented as a vital messenger in the adaptive response to abiotic stresses, and its role in stress resistance has gained much consideration. Endogenous ABA levels rise quickly in response to abiotic stress, triggering specific signaling pathways and altering gene expression levels (Trivedi et al., 2016; Yao et al., 2020; Golfazani et al., 2022).

Several studies have investigated ABA’s antioxidant potential in plants subject to stressful environments (Table 1). Reports have linked ABA-induced abiotic stress tolerance with glutathione (GSH) and ascorbate (AsA) in higher plants. Wheat (*Triticum aestivum* L.) plants subjected to exogenous ABA under drought stress enhanced drought tolerance by regulating genes involved in GSH/AsA synthesis, consequently increasing GSH and AsA activities in leaves and roots and decreasing malondialdehyde (MDA) and H_2_O_2_ levels. Due to the regulation of GSH/AsA synthesis, ABA-mediated drought stress in wheat seedlings by improving the plant phenotype, including leaf wilting, stunted plant height, and growth, and quantitatively enhanced overall physiological and growth parameters, including plant height (11.4%), shoot fresh weight (21.8%), shoot dry weight (21.4%), root fresh weight (34.9%), and root dry weight (23.1%) (Liting et al., 2015). In another study, the flavanone 3-hydroxylase (*PnF3H*) gene from Antarctic moss was evaluated in *Arabidopsis* under cold, salinity, osmotic, and UV radiation stresses; exogenously applied ABA increased tolerance to salt and oxidative stresses, improved SOD and CAT activities under salt and oxidative stress, and altered the flavonoid constituents of transgenic plants (Li et al., 2017a). An early root growth assay showed improved root phenotypes in the ABA treatments (0.5 and 0.75 μM), with *PnF3H*-overexpressing lines (AtOE) exhibiting better germination rates and longer primary roots (Li et al., 2017a). Likewise, 30 μM exogenous ABA applied to wheat plants increased endogenous ABA and SOD, CAT, and POD activities and improved drought tolerance (Bousba et al., 2020). In Chinese arborvitae (*Platycladus orientalis*) seedlings, exogenous ABA (0.5, 1, 10, 100, and 200 μM) significantly mitigated oxidative stress by modulating ROS metabolism, increasing SOD, POD, and CAT activities (by 69%, 91%, and 94%, respectively), AsA, total GSH, and proline contents, and decreasing H_2_O_2_ and MDA contents under H_2_O_2_ stress (Yao et al., 2020). According to their results, the 1 μM L^−1^ ABA treatment had the greatest increase in transcript levels of antioxidant enzyme-encoding genes such as *Cu/Zn-SOD*, *CAT*, *GR*, *APX*, and *MDAR* under H_2_O_2_-induced stress.

Abscisic acid can reduce oxidative stress in plants exposed to metal stress by stimulating antioxidant mechanisms. Kamran et al. (2021a) established that ABA and 6-benzylaminopurine (BAP) applied at 0.5 and 10 μM (individually or in combination) to tomato (*Solanum lycopersicum* L.) seedlings reduced the effects of oxidative stress caused by 400 μM cobalt (Co). The treatments, especially ABA + BAP at 10 μM, augmented SOD, APX, CAT, and POD activities, reduced MDA and H_2_O_2_ levels, increased chlorophyll *a*, *b*, and carotenoid contents, ameliorated root and shoot biomass and length, improved membrane stability and leaf relative water content (RWC), and decreased Co uptake and accumulation (Kamran et al., 2021a). Similar results occurred in mung bean (*Vigna radiata* L.) plants subjected to 50 and 100 μM Cd. Foliar application of ABA (5, 10, and 15 μM) improved the physio-biochemical processes of plants exposed to Cd stress, including increased roots and shoot weight and length, inhibiting Cd accumulation in roots and shoots, enhancing carotenoid content, augmenting the activities of POD (stems and leaves), CAT (roots and leaves), and APX (stems), and boosting the contents of AsA (roots and stems), polyphenols (roots), and proline (stems and leaves) (Leng et al., 2021). According to Cao et al. (2021), proline accumulation and the expression of genes encoding enzymes in the proline biosynthesis pathway (e.g., Os*P5CS1*, Os*P5CS2*, and Os*ProDH*) need ABA to mediate proline biosynthesis in rice (*Oryza sativa* L.) under hypoxia stress. Treatment with an ABA synthesis inhibitor (norflurazon) inhibited proline synthesis and exacerbated oxidative stress, both of which were reversed by exogenous ABA application.

A glutathione peroxidase gene (*GPX6*) is a redox-related gene up regulated under copper stress in response to ABA (Milla et al., 2003). *GPX3* is involved in H_2_O_2_ homeostasis and signal relaying in guard cells, regulating ROS levels and stomata according to ABA levels (Miao et al., 2006). While the ABA signal induction mechanism activates the expression of antioxidant enzymatic and non-enzymatic defense systems genes, ABA also induces secondary messengers activating defensive responses through ROS production (Sakamoto et al., 2008). Direct molecular genetics and cell biological evidence show that ROS are rate-limiting secondary messengers in ABA signaling. Kwak et al. (2003) reported that disruption of two partially redundant *Arabidopsis* guard-cell-expressed NADPH oxidase catalytic subunit genes (*AtrbohD* and *AtrbohF*) impairs ABA signaling. In tomato seedlings, saline–alkaline stress-induced ABA synthesis and signal transduction. Exogenously applied ABA relieved the saline–alkaline stress by regulating osmotic adjustment and Chl contents, promoting the accumulation of proline and soluble sugars, reducing ROS content, and improving the antioxidant defense system (Xu et al., 2022a). In conclusion, ABA improves the abiotic stress performance of crop plants by modulating ROS metabolism and improving the antioxidant defense system.

### Jasmonic acid

Jasmonic acid/jasmonates are fatty acid derivatives that include compounds such as jasmonate (JA), jasmonate iso-leucine conjugate (JA-Ile), and methyl jasmonate (MeJA) (Wasternack and Strnad, 2018). These endogenous signaling molecules are involved in diverse developmental processes in higher plant species and were previously known as stress-related hormones (Wasternack and Strnad, 2018; Lang et al., 2020; Raza et al., 2021c). Notably, JA is the best-characterized, most well-known, and most abundant jasmonate. The antioxidant potential of JA under several abiotic stresses has been explored (Table 1).

One study treated apple (*Malus pumila* L.) seedlings with JA or JA biosynthesis inhibitor (Ibuprofen, IBU) to explore how the AsA-GSH cycle responds to oxidative injury caused by cold stress (Li et al., 2017b). The study found that exogenous JA stimulated AsA synthesis by increasing DHAR activity and concluded that MDHAR is an auxiliary pathway, regardless of the increased MDHAR activity. Furthermore, the IBU treatment significantly inhibited antioxidant enzymes (APX, DHAR, and MDHAR), indicating that endogenous JA participated in the antioxidant process via gene expression by modulating the AsA-GSH cycle in the low-temperature treatment. Chen et al. (2014) also showed that exogenous MeJA induced endogenous JA accumulation in mangrove (*Kandelia obovata* L.) seedlings and simultaneously enhanced the antioxidant capacity.

Pakar et al. (2016) evaluated the growth, development, antioxidant system activity, and ion storage of barley (*Hordeum vulgare* L.) supplied with exogenous JA under salt stress. The JA treatment increased salt tolerance by improving SOD, CAT, and POX activities and K^+^/Na^+^ accumulation efficiency. In addition, JA significantly improved plant height and peduncle length in control and 5 dS m^–1^ salinity treatments. It also increased overall leaf area and grain number, particularly grain number in the 15 dS m^–1^ treatment (13.5% decline) compared to the control (24.0% decline) (Pakar et al., 2016). Sirhindi et al. (2016) showed that soybean (*Glycine max* L.) supplemented with JA could overcome Ni stress, boost osmolytes, enhance expression of antioxidant enzyme genes, and increase the activities of SOD (40.04%), POD (28.22%), CAT (48.53%), and APX (56.79%). Relative to the controls, Ni-treated plants with JA supplementation had reduced NADPH oxidase activity (30.04%), improved shoot length (30.74%), and a slightly reduced germination rate (8.16%). Furthermore, JA stimulated overall plant growth, increasing root length (70.06%), dry weight (11.47%), and total chlorophyll content (38.70%) (Sirhindi et al., 2016).

Najafi-Kakavand et al. (2019) reported that two populations of *Alyssum inflatum* exposed to Ni (100, 200, and 400 μM) and treated with JA (5 and 10 μM) and SA (50 and 200 μM) alone or combined reduced oxidative stress, improving biomass and Ni toxicity tolerance. Thus, JA and SA inhibited ROS by triggering SOD, CAT, POD, and APX activities and increasing proline and carotenoid contents. The treatments also reduced Ni translocation from roots to aerial parts, preventing Ni accumulation in shoots and reducing Ni toxicity. Sugar beet (*Beta vulgaris* L.) plants under drought stress treated with JA (10 μM) had increased activities of several antioxidant enzymes (APX by 290%, CAT by 80%, and POX by 94%), root biomass (21%), and stress tolerance (Ghaffari et al., 2020).

Chinese licorice (*Glycyrrhiza uralensis* L.) plants exposed to salinity and supplemented with different concentrations of MeJA had increased salt tolerance compared to stress-free control plants (Lang et al., 2020). Notably, MeJA impacted enzyme activities in a concentration-dependent way, increasing SOD activity at 45 μM, POD activity at 30 μM, CAT activity at 15 μM, and GPX activity at 30 μM, and reducing APX activity at 30 μM. MeJA also mediated the adverse effects of salt stress on seedlings, with significant improvements in hypocotyl diameter, hypocotyl length, and radicle length at 45 μM, hypocotyl diameter and radical length at 30 μM, and radicle diameter at 15 and 30 μM (Lang et al., 2020). In mustard (*Brassica parachinensis* L.) exposed to chromium (Cr) stress (150 and 300 μM), exogenous JA application, especially at 10 μM, improved growth and photosynthesis by reducing oxidative stress, boosting the activities of APX, SOD, CAT, GR, GST, GPX, MDHAR, DHAR, and glyoxalase enzymes, and enhancing AsA and GSH contents, which increased the uptake of nutrient elements and limited Cr accumulation (Kamran et al., 2021b). In photothermosensitive-genic-male-sterile (PTSGMS) rice lines exposed to high-temperature stress at the flowering stage (anthesis), the MeJA treatment augmented CAT activity and AsA content, reducing H_2_O_2_ levels in stigmas and improving plant vitality, thus increasing fertility and seed set (Chen et al., 2021a). Regarding the possible mechanism, JA and its methyl ester can induce ethylene production, leading to ROS generation in plants (O’Donnell et al., 1996; D’Haeze et al., 2003; Chen et al., 2020). However, little is known about the crosstalk between ethylene and antioxidant enzymes for mediating ROS generation under stress. Still, ethylene and ROS can positively affect plant growth and development at low concentrations (D’Haeze et al., 2003).

In a recent study, Yan et al. (2022) showed that 100 μM MeJA improved tomato drought tolerance and increased biomass. The MeJA treatment enhanced root exudation and whole plant respiration rate to maintain relatively high water content. Moreover, MeJA improved the expression of carotenoid cleavage dioxygenase and reduced the ABA level, maintaining high stomatal conductance and consequently improving tomato drought tolerance (Yan et al., 2022). In soybeans, foliar application of JA (100 μmol L^–1^) alleviated the adverse effects of salt stress by increasing the photosynthetic pigments Chl *a* and Chl *b* concentrations of soluble proteins and phenol in the leaves (Noor et al., 2022). Moradi et al. (2022) revealed that salinity (50 mM NaCl) increased H_2_O_2_ and proline contents and SOD activity and decreased the MSI percentage and total soluble protein content. Silicon nanoparticles and MeJA decreased the H_2_O_2_ content and increased the transcription level of salinity-related genes like *DREB, cAPX, MnSOD*, and *GST* (Moradi et al., 2022). Thus, JA grants plant tolerance to various abiotic stresses and aids in developing crops adaptable to climate change.

### Salicylic acid

Salicylic acid (SA) is a unique phenolic compound that modulates pathogenesis-related protein expression. Besides defense responses, SA has roles in stimulating plant growth, ripening, expansion, and abiotic stress responses (Per et al., 2017; Jahan et al., 2019). The biosynthesis of SA arises via the isochorismate (IC) and phenylalanine ammonia-lyase (PAL) pathways (Per et al., 2017). Notably, low levels of SA increase antioxidant defense in plants (Table 1); however, high levels lead to cell death or vulnerability to abiotic stresses (Jumali et al., 2011). For example, Jumali et al. (2011) found that high SA levels increased sarcosine oxidase gene expression with time, catalyzing the oxidative demethylation of sarcosine and generating H_2_O_2_ (Nishiya, 2000).

In barley, exogenous SA increased cold tolerance by modulating SOD, CAT, and POX activities (Mutlu et al., 2016). Under salt stress, SA reduced K^+^ leakage in *Arabidopsis* root tissues and increased H^+^-ATPase activity to induce Na^+^/H^+^ exchanger at the plasma membrane and reduce Na^+^ accumulation in the cytosol (Jayakannan et al., 2013). In Ethiopian mustard (*Brassica carinata* L.), foliar SA supplementation alleviated salt-induced damage by increasing the efficiency of SOD (29–32%), CAT (25–27%), and POX (179–194%) (Husen et al., 2018). In two mustard varieties, Adet and Merawi, SA application mediated salt stress by significantly improving plant growth parameters. Adet had a higher biomass yield than Merawi, but root biomass did not significantly increase at 0.5 mM SA. For Adet treated with 50 mM NaCl, co-application of SA (0.5 mM) and NaCl yielded the maximum leaf number (11.24), root length (13.79 cm), shoot length (42.83 cm), and leaf width (53.85 mm) (Husen et al., 2018). Similarly, heat-stressed tomato treated with exogenous SA increased the activity of SOD (36%), POD (136%), CAT (250%), and APX (65%) by enhancing photosynthesis and antioxidant enzyme efficiencies (Jahan et al., 2019). The SA treatment decreased oxidative injury and improved heat tolerance. Seed pretreatment with 1.0 mM SA enhanced heat stress tolerance by improving phenotypic traits (Jahan et al., 2019). Pigeon pea (*Cajanus cajan* L.) plants pre-treated with SA (0.5 mM and 1.0 mM) and subjected to heat stress had improved heat tolerance and increased activity of CAT (0.77-fold with 0.5 mM SA and 1-fold with 1.0 mM SA) and POX (1.24-fold with 0.5 mM SA and 1.37-fold with 1.0 mM SA) (Kaur et al., 2019).

Mustard plants (*Brassica juncea* L.) exogenously treated with SA (0.25 mM) alleviated Pb-induced oxidative injury and increased the activities of several antioxidant enzymes and compounds such as APX, GR, POD, CAT, MDHAR, DHAR, AsA, and GSH (Hasanuzzaman et al., 2019). SA application also increased the GSH/GSSG ratio and improved overall plant growth and biomass production; 0.25 mM Pb(NO_3_)_2_ and 0.25 mM SA applied 45 days after sowing produced the maximum plant height (45.50 cm), fresh weight (16.00 g), and dry weight (3.08 g), while 1.0 mM Pb(NO_3_)_2_ and 0.25 mM SA did not significantly improve growth or biomass but enhanced seed yield by 15% (Hasanuzzaman et al., 2019). Similarly, 1.0 mM SA increased SOD, CAT, and POD activities and enhanced proline and glycine betaine contents in basil (*Ocimum basilicum*), subsequently reducing the adverse effects of H_2_O_2_ and MDA induced by drought stress due to improved chlorophyll a and b contents, CO_2_ assimilation rate, stomatal conductance, and transpiration rate (Zulfiqar et al., 2021). Likewise, Wang et al. (2021) revealed that 0.1 mM foliar SA spray to rice exposed to Cd (1 mg kg^–1^) until the flowering stage stimulated antioxidative mechanisms, reduced oxidative stress, and alleviated seed Cd accumulation. In rice under Cd stress, SA served as a hydroxyl radical scavenger (Yang et al., 2004; Liu et al., 2016). As a scavenger, SA modulated the expression of Cd transporter genes (*OsLCT1* and *OsLCD*) in the aerial parts of rice by regulating H_2_O_2_ quantity and its associated signaling, preventing Cd storage in shoots and grain (Wang et al., 2021).

In sorghum (*Sorghum bicolor* L.), exogenous SA improved salt stress tolerance by inducing proline accumulation, antioxidant enzyme activities, increased the protection of photosynthetic machinery, maintained photosynthetic activities, and improved plant growth (Rajabi Dehnavi et al., 2022). Under saline conditions at 150 mg L^–1^, SA is considered the most effective for preventing the damaging effects of salinity (Rajabi Dehnavi et al., 2022). In another study, Fatima et al. (2022) revealed that 0.5 and 1 mM foliar SA spray to apple mint (*Mentha suaveolens*) plants exposed to Cu (40 mM) decreased Cu concentrations in various plant parts, which was accompanied by increases in K, P, and Ca concentrations. SA also exerted a remedial effect on the performance of essential oils, mainly at 0.5 mM (Fatima et al., 2022). Plant hormones and antioxidants play a vital role in fruit ripening, quality, and parthenocarpic fruit formation (Irfan et al., 2021; Su et al., 2021; Sharif et al., 2022; Tayal et al., 2022). Under water-stressed conditions, foliar-applied SA reduced MDA and H_2_O_2_ contents and ion leakage and increased SOD and POX activities under all irrigation regimes (Biareh et al., 2022). Stress and SA spraying increased fruit quality by increasing soluble carbohydrates and decreasing citric acid content (Biareh et al., 2022). Regarding oil quality, water stress increased saturated fatty acid content but spraying SA increased linoleic and oleic acid contents (Biareh et al., 2022). Consequently, SA significantly helps plants to react and cope with environmental challenges.

### Brassinosteroids

Brassinosteroids (BRs) are a comparatively innovative set of polyhydroxy steroidal plant hormones that encourage robust growth and development. To date, more than 70 BRs have been extracted from plants; brassinolide, 28-homobrassinolide, and 24-epibrassinolide have the greatest bioactivity and are most popular in stress-related physiological investigations (Tanveer, 2019; Tanveer et al., 2019; Nolan et al., 2020; Hafeez et al., 2021). These plant hormones are present in most plant parts (Vukašinović and Russinova, 2018; Nolan et al., 2020) and play critical roles in numerous developmental processes and improve plant health under stressful conditions (Tanveer, 2019; Tanveer et al., 2019; Hafeez et al., 2021). In particular, BRs and related compounds have the potential to modify the antioxidant defense-mediated stress-bearing potential of plants under multiple abiotic stresses (Table 1).

While water deficit conditions hamper cowpea growth and development, 24-epibrassinolide (EBR; active BR) reduced cell damage and increased stress tolerance by mitigating ROS (O_2_^–^ and H_2_O_2_) accumulation through the increased activity of SOD (25%), CAT (29%), APX (50%), and POX (149%). It also improved PSII efficiency under stress conditions. In addition, EBR (100 nM) increased leaf, shoot, and root dry biomass by 11%, 7%, and 10%, respectively, and total dry biomass (10%) (Lima and Lobato, 2017). BR improved cold tolerance in *Elymus nutans*, enhancing the activities of CAT, APX, GR, DHAR, and MDAR but not SOD (Fu et al., 2019). Notably, 0.1–1 μM EBR improved plant growth under cold stress, but higher concentrations decreased fresh weight (Fu et al., 2019). In soybean, exogenous EBR application (50 and 100 nM) improved drought tolerance, with 50 nM EBR increasing the activities of SOD (20%), CAT (40%), APX (28%), and POX (14%), and 100 nm EBR further increasing these activities.

Soybean seedlings subjected to water deficit reduced the diameters of root epidermis, root cortex, root endodermis, vascular cylinder, and root metaxylem; however, 100 nM EBR increased these parameters by 21%, 15%, 12%, 38%, and 15% (Dos Santos Ribeiro et al., 2019). Moreover, the 100 nM EBR treatment improved root length (18%), hypocotyl length (7%), seedling length (15%), root dry matter (58%), hypocotyl dry matter (11%), and seedling dry matter (13%), with corresponding increases of 16%, 4%, 12%, 16%, 4%, and 5%, respectively at 50 nM EBR. The 100 nM EBR treatment also promoted seed germination by 6% in the first count to germination and 4% in total germination (Dos Santos Ribeiro et al., 2019). The authors also mentioned that reductions in ROS (superoxide and H_2_O_2_) concentrations and membrane damage (MDA and electrolyte leakage) were intrinsically related to the higher activities of antioxidant enzymes, confirming the benefits of BRs on the antioxidant system. In another study, maize plants subjected to salt stress were pre-treated with various concentrations of 28-homobrassinolide (HBL) or EBR (Rattan et al., 2020). In plants pre-treated with 0.0001 μM HBL and 0.01 μM EBR, POD activity increased by 20.97% and 22.01%, respectively. Pretreatment with 1.0 μM HBL and 0.01 μM EBR increased CAT activity by 62.34% and 11.75%. Similarly, 0.01 μM HBL and 1.0 μM EBR improved DHAR activity by 17.82% and 27.87%, respectively, while 0.01 μM HBL and 0.01 μM EBR enhanced MDHAR activity by 7.6% and 14.28%, respectively (Rattan et al., 2020). Thus, BR application helps preserve cellular conditions by regulating ion metabolism, enhancing osmoprotectant accumulation, and strengthening the antioxidative defense system in salinity-stressed seedlings.

Heidari et al. (2021) showed that EBR foliar spray (5 mg L^–1^) to two tomato cultivars (cold-sensitive and tolerant) subjected to 9°C reduced the adverse effects of ROS by increasing antioxidant enzyme activities, including CAT and GPX. In addition, endogenous levels of IAA and GA_3_ increased, especially in the cold-sensitive cultivar, augmenting the growth of those plants. EBR application also enhanced ABA levels at 9°C, constituting a synergistic response to cold stress (Heidari et al., 2021). A 1.0 mg L^–1^ EBR pretreatment to high-temperature-stressed melons improved photosynthesis and photochemical activity and balanced the distribution of excitation between photosystems to avoid ROS production (Zhang et al., 2013). A 2-year field experiment on dragon’s head (*Lallemantia iberica*) at four irrigation levels revealed that foliar BR application (0, 0.5, 1, and 1.5 μM) counteracted the adverse effects of drought stress by triggering antioxidative mechanisms, including increasing CAT, SOD, POD, and PPO activities, ROS scavenging, and proline content. However, BR had no significant effect on membrane stability or grain yield (Naservafaei et al., 2021). While BRs can mitigate environmental stresses by activating antioxidant enzymes and decreasing ROS, they may also be involved in ROS production and act in BR-mediated ROS signaling, which is essential for some plant developmental processes such as tapetum degradation and pollen fertility in tomato (Yan et al., 2020). In this process, signaling regulator BRASSINAZOLE RESISTANT 1 (BZR1) could directly bind to the promoter of RESPIRATORY BURST OXIDASE HOMOLOG 1 (RBOH1), mediating ROS production, which promotes pollen and seed development by triggering PCD and tapetal cell degradation (Yan et al., 2020). Loss or gain of function in the BR biosynthetic DWARF (DWF) or *BZR1* genes altered the timing of ROS production and PCD in tapetal cells, resulting in delayed or premature tapetal degeneration (Yan et al., 2020). Basit et al. (2022a) showed that seed priming with BRs and spermine (SPM) mitigated Cr toxicity by limiting its uptake in rice. BR and SPM application improved the seed germination rate, Chl content, PSII system, and total soluble sugars, minimizing ROS production, MDA content, and electrolyte leakage under Cr stress. In addition, BRs and SPM controlled antioxidant enzyme and non-enzyme activities to diminish Cr-induced cellular oxidative losses (Basit et al., 2022a). Likewise, brassinosteroids insensitive 1 (*BRI1*) is a BR receptor that triggers BR signaling. Wang et al. (2022a) revealed that chilling stress rapidly induced *SlBRI1* expression in tomatoes, reaching its highest level at 3 h, resulting in low relative electrolyte leakage, MDA content, and ROS accumulation. The *SlBRI1OE* plants had higher proline contents and SOD, POD, and CAT activities than the control. These outcomes reveal that *SlBRI1* positively regulates chilling tolerance mainly through the ICE1–CBF–COR pathway in tomatoes (Wang et al., 2022a). In another study, BRs and H_2_O_2_ signaling regulated melatonin-induced drought and cold tolerance in perennial ryegrass (*Lolium perenne*) (Fu et al., 2022). As a result, BRs interact under varied abiotic stress situations and improve the ability of plants to withstand stress and defend themselves.

### Indole-3-acetic acid/auxin

Despite being the subject of many studies, indole-3-acetic acid (IAA, or auxin) biosynthesis, transport, and signaling pathways are not well understood, mainly under stress conditions. Nonetheless, a few interlocking pathways have been proposed for auxin biosynthesis in plants, including four tryptophan-dependent and independent pathways (Brumos et al., 2018; Blakeslee et al., 2019). IAA is a multifaceted phytohormone with dynamic plant growth/development roles, particularly under stressful environments (Blakeslee et al., 2019). There is increasing evidence that IAA is essential for plant adaptation to various stresses because it modulates the antioxidant defense system (Table 1); however, more investigations are needed to understand the regulatory role of IAA.

Three wheat varieties treated with IAA under drought stress increased SOD activity by 33%, 15%, and 38%, and POD activity by 90%, 77%, and 82%, respectively, increasing drought tolerance (Muhammad et al., 2016). In the same study, foliar IAA application restored plant fresh weight by increasing RWC. Xing et al. (2016) reported that NAA (α-naphthaleneacetic acid) application to soybean seedlings under drought stress increased endogenous IAA and ABA, H_2_O_2_ accumulation, and antioxidant enzyme activities during early and late stress phases, improving drought tolerance. Since auxins contribute to plant growth regulation, they can alleviate the deleterious effects caused by abiotic stresses. For example, the application of 25 ppm IAA to sunflower (*Helianthus annuus* L.) (cultivars Sakha53 and China) under salt stress (120 mM NaCl) resulted in the highest salt tolerance index values for shoot fresh biomass (161.96% and 188.62%), shoot dry biomass (229.52% and 201.66%), root fresh biomass (202.48% and 167.30%), root dry biomass (292.50% and 370.00%), leaf area (167.97% and 152.84%), shoot length (136.82% and 162.74%), and root length (168.08% and 169.70%) (Zayed et al., 2017). In the same study, IAA supplementation had improved CAT activity (152% and 350%) and PPO (110% and 100%) (Zayed et al., 2017). In another study, potato plants pre-treated with IAA (7 and 14 μM) *in vitro* under various salt stress levels (0, 40, 60, and 80 mM NaCl) had less oxidative injury than untreated plants due to improved SOD and POD activities (Khalid and Aftab, 2020). Moreover, a 30-day treatment with 14 μM IAA improved callus fresh weight in plants subjected to 40 and 60 mM NaCl. Indeed, IAA application increased the root length, root number, and overall root growth for plants exposed to up to 80 mM NaCl. Additionally, the positive impact of IAA was evident in morphological changes, i.e., dark brown to light brown callus, indicating the elimination of necrotic symptoms (Khalid and Aftab, 2020).

In spinach (*Spinacia oleracea* L.) exposed to copper (Cu) toxicity, leaves treated with various IAA concentrations (10–60 mg L^–1^) decreased the toxic effect of Cu and improved plant health by increasing the activities of SOD (46%), POD (57%), and APX (99%) (Gong et al., 2020). Moreover, IAA application under Cu stress increased total plant fresh and dry weights; 60 mg L^–1^ IAA produced the highest fresh (49.17%) and dry (69.99%) weights relative to the control. Moreover, the combined total plant biomass (leaves, stems, and roots) also increased with increasing IAA concentration (Gong et al., 2020). Similarly, tea (*Camellia sinensis*) plants treated with IAA (2 μM and 10 μM) under cadmium (Cd) stress decreased MDA content, and improved plant growth. It mitigated Cd-induced oxidative stress by modulating CAT, APX, POD, and SOD activities (Zhang et al., 2020). Plant height and diameter increased by 9.8% and 9.2% at 2 μM IAA (T1) and 18.1% and 36.5% at 10 μM IAA (T2), respectively, relative to the control. The IAA applications also significantly increased root vigor by 46.5% in T1 and 56.9% in T2 (Zhang et al., 2020). Similarly, 5 μM IAA applied to eggplant (*Solanum melongena*) under 25 μM arsenate (AsV) ameliorated root length and biomass by reducing AsV accumulation, amending the adverse effects of ROS, sequestrating AsV in cells, and ameliorating GSH redox status (Alamri et al., 2021). Similarly, in faba bean (*Vicia faba*), exogenous IAA application (200 ppm) ameliorated growth by improving tolerance against salinity stress (60 and 150 mM NaCl). Thus, IAA is involved in the osmotic protection of roots, shoots, and seeds by regulating proline, soluble sugars, free amino acids, and protein contents. It mediates improvements in K^+^, Ca^2+^, and Mg^2+^ homeostasis and Na^+^ translocation but inhibits root Na^+^ accumulation. IAA also reduces oxidative stress due to salinity by improving SOD, CAT, POD, and APX activities, thus increasing root and shoot growth, biomass, and nodule number (Abdel Latef et al., 2021a,b). According to Molassiotis et al. (2010), IAA and other plant hormones can move from salt-treated roots to leaves to induce NO synthesis, trigger NO transport throughout the plant, and generate defense responses following salt stress. He et al. (2022) revealed that 20 mg L^–1^ exogenous IAA application significantly increased rice (*Oryza sativa*) growth and reduced As accumulation in grain. In another study, IAA and Si-NPs alleviated Cr-IV stress in rice seedlings; Cr-IV increased ROS levels, whereas IAA and Si-NPs detoxified ROS to enhance plant tolerance and defense mechanisms (Sharma et al., 2022b). Likewise, exogenous IAA (10 mg kg^–1^) relieved Cd stress (30 mg kg^–1^) in ryegrass (*Lolium perenne*) and improved Cd absorption (Xu et al., 2022b). IAA increased ryegrass biomass, Chl content, SOD activity, sucrose activity, fluorescein diacetate (FDA) hydrolase activity, and Cd removal rates by 14.5%, 19.9%, 24.3%, 12.1%, 20.4%, and 15.1%, respectively, but decreased POD activity, soil basal respiration, and Cd soil residues by 8.0%, 15.0%, and 17.0%, respectively, compared with the control (Xu et al., 2022b). So, IAA improves plant tolerance and antioxidant defense mechanisms, reducing the oxidative damage brought on by abiotic stress.

### Cytokinins

Cytokinins (CKs) are byproducts of purine bases with an isoprenoid or aromatic side chain at the *N*^6^ site. This class of molecules includes zeatin (Z), dihydrozeatin (DZ), and *N*^6^-(Δ^2^-isopentenyl) adenine (iP) (Márquez-López et al., 2019). CKs play influential roles in various growth and developmental processes, dominating under stress conditions. Table 1 documents some examples of the antioxidant potential of CKs under stressful environments.

Evidence suggests that CKs have positive and negative effects on stress tolerance. For example, endogenous CK concentrations significantly decreased under extended stresses (Merewitz et al., 2011). Wang et al. (2015) suggested that reduced CK homeostasis in plant cells modifies salt stress responses in *Arabidopsis* through ROS-mediated regulation. They concluded that endogenous CK overproduction (via *AtIPT8* overexpression) negatively affected plant salt tolerance by modulating stress-responsive gene expression, ROS production, and Chl contents. Interestingly, ROS production also increased in the salt treatment, along with the endogenously overproduced CKs. However, many other studies report positive effects of CKs on stress tolerance.

Salt-stressed perennial ryegrass plants treated with BAP (25 μM) mitigated salt stress by increasing the activities of various antioxidant enzymes and molecules, including AsA (67.22%), GSH (9.00%), SOD (17.82%), CAT (39.91%), APX (91.46%), MDHAR (six-fold), and GR (66.67%) (Ma et al., 2016). Similarly, exogenous BAP application increased turf quality and leaf length by up to 19.43% and 26.83%, respectively, compared to the unalleviated salt stress condition. Moreover, the yellow to green leaf area ratio declined by 55.46% after BAP exposure (Ma et al., 2016). Foliar supplementation with CKs in creeping bentgrass (*Agrostis stolonifera*) increased drought tolerance and enhanced the activities of SOD (25%), APX (22%), CAT (17%), and POD (24%) under drought stress. These results indicate that foliar treatment with CKs boosts physiological responses, the antioxidant system, and stress tolerance (Chang et al., 2016).

In another study, green alga (*Chlorophyta*) under Pb toxicity supplemented with CKs had an inhibitory effect on Pb accumulation, increasing cell numbers by up to 149% compared to the control. The CKs treatment also enhanced the activities of AsA (41%), GSH (76%), SOD (76%), CAT (41%), GR (110%), and APX (43%), increased proline content (126%), and improved plant adaptation to Pb toxicity (Piotrowska-Niczyporuk et al., 2018). Similarly, exogenous application of BAP (50 μM) to faba bean under salt stress enhanced salt tolerance and increased APX and SOD activities (Samea-Andabjadid et al., 2018); similar results occurred in another study on faba bean under salinity stress (150 mM NaCl) treated with BAP (0.9 mM) (Abdel Latef et al., 2021a). Bashri et al. (2021) reported that foliar application of kinetin at 10, 50, and 100 μM to fenugreek (*Trigonella*) seedlings grown in Cd-contaminated soils (3 and 9 mg kg^–1^) improved growth by reducing H_2_O_2_ content. By stimulating APX, GR, and DHAR activities, which are all involved in the ascorbate-glutathione cycle, and increasing photosynthetic pigment contents, kinetin improved the photosynthesis rate under Cd stress. Avalbaev et al. (2021) investigated the combined effect of 0.1 μM MeJA and 0.04 μM cytokinin 6-benzylaminopurine (BAP) on salinity-induced oxidative injury in wheat (*Triticum aestivum* L.) seedlings. The MeJA and BAP treatment reduced the salt-induced pro-oxidants/antioxidants imbalance and membrane damage, increased the accumulation of proline and dehydrins, and improved the barrier properties of cell walls in seedling roots by accelerating lignin deposition. Thus, endogenous CKs in MeJA-induced manner increase the salinity tolerance of wheat plants (Avalbaev et al., 2021). Likewise, under saline conditions (4, 7, and 10 dS m^–1^ NaCl), a foliar spray of 50 μM of BAP decreased root and leaf Na^+^ contents and increased root and leaf K^+^ contents, shoot growth, and amino acid content and composition in seeds of faba bean (*Vicia faba* L.) (Ghassemi-Golezani and Samea-Andabjadid, 2022). To conclude, CKs is a central regulator in plant growth and abiotic stress responses.

### Gibberellins

Gibberellins (GAs) are a vast set of tetracyclic diterpenoid carboxylic acids, with only some acting as growth hormones in higher plants, mainly GA_1_ and GA_4_ (Hedden, 2018). There is growing evidence of the dynamic roles of GAs in abiotic stress responses and tolerance (Colebrook et al., 2014; Hedden, 2018; Ullah et al., 2022). Recent studies have explored the antioxidant role of GAs under numerous stresses (Table 1). Notably, these hormones cooperate with other plant hormones in many evolving and stress-responsive processes.

In okra (*Abelmoschus esculentus* L.) exposed to salinity, foliar application of GA_3_ (0.1 mM) reduced the adverse effects of NaCl by enhancing growth attributes, chlorophyll and carotenoid contents, and SOD, CAT, and POD activities, and reduced electrolyte leakage (EL), MDA content, and H_2_O_2_ content (Wang et al., 2019). Furthermore, shoot and root lengths increased from 22.7 cm and 9.9 cm (salt stress only) to 28.2 cm and 10.8 cm (salt stress + GA_3_ application), respectively, as did root and shoot fresh and dry weights (Wang et al., 2019). Likewise, combined GA_3_ (1.4 μM) and melatonin, exogenously applied to tomato plants, enhanced Chl content and reduced the over-generation of ROS and glycolate oxidase activity. Both melatonin and GA_3_ protected seedlings from ROS damage by regulating Δ1-pyrroline-5-carboxylate synthetase activity, an intermediate product of proline biosynthesis and catabolism (Siddiqui et al., 2020). Proline behaves like a non-enzymatic antioxidant, and its metabolism propels cellular signaling processes for promoting cellular apoptosis or survival (Liang et al., 2013). Siddiqui et al. (2020) reported that GA application boosted shoot and root lengths by 72.2% and 56.7%, shoot and root fresh weights by 195.5% and 133.3%, and shoot and root dry weights by 307.2% and 215.8%, respectively, and the activities of CAT (49.7%), APX (45.4%), GR (152.0%), DHAR (39.2%), MDHAR (98.1%), GPX (63.2%), polyphenol oxidase, lipoxygenase, and redox homeostasis, thus decreasing the oxidative stress induced by NaCl stress. In bracted bugleweed (*Ajuga integrifolia*) shoot culture, exogenous GA application increased biomass in a dose-dependent manner, with 1, 2.5, and 5 mg L^–1^ increasing dry weights by 15.25, 16.27, and 17.97 g L^–1^, respectively (Abbasi et al., 2020a).

Wheat treated with foliar spray of GA (100 and 200 mg L^–1^) to mitigate cerium oxide nanoparticles (CeO_2_-NPs) significantly increased plant growth, yield, and Chl and nutrient contents compared to the control (Iftikhar et al., 2020). GA also reduced oxidative stress induced by CeO_2_-NPs by increasing CAT, SOD, APX, and POD activities, and increased plant height, spike height, root and shoot dry weights, and grain dry weight, with the GA effects more pronounced at 200 mg L^–1^ than 100 mg L^–1^ (Iftikhar et al., 2020). Similarly, in sweet cherry (*Prunus avium* L.) exposed to chilling stress, CAT, SOD, POD, and APX activities increased after 5 days of GA_4_ treatment. In addition, GA_4_ acted as a dormancy-breaking agent, producing earlier flower buds than other applied agents (Cai et al., 2019). In date palm (*Phoenix dactylifera* L.) under heat stress, exogenous GA_3_ improved growth traits, such as shoot and root lengths and fresh and dry weights, and CAT, POD, APX, and polyphenol oxidase activities and decreased oxidative stress, with noteworthy reductions in MDA and superoxide anions (Khan et al., 2020). The greatest improvements in growth parameters occurred in the GA_3_ treatment combined with silicon (Khan et al., 2020). In great millet (*Sorghum bicolor*) seedlings, salinity stress (0, 100, and 200 mM NaCl) led to oxidative stress, reducing germination percentage, biomass, growth, and photosynthetic pigment content; exogenous GA_3_ treatments (0, 144.3, 288.7, and 577.5 μM) and nitrogen fertilizer (0, 90, and 135 kg N ha^–1^) reduced the oxidative stress by increasing SOD and POD activities, thus improving photosynthesis and seedling growth (Ali et al., 2021). Hakla et al. (2021) reported similar results in a study on mung bean (*Vigna radiata*) exposed to Cd toxicity (CdCl_2_, 500 μM); GA_3_ at 500 μM improved the adverse effects of Cd stress on plant growth, photosynthetic apparatus, and metabolic processes. Salinity stress (150 mM NaCl) in wheat reduced germination, biomass, and photosynthetic pigments. The exogenous GA_3_ treatment (150 mg L^–1^ g) alleviated the salt stress, improving growth and yield, reducing oxidative stress, and enhancing various growth parameters (Iqbal et al., 2022). Islam et al. (2022) reported that foliar application of CK and GA_3_ improved the growth and biomass of mung bean exposed to 5 days of waterlogging. CK and GA_3_ reduced the elevated MDA and ROS levels and improved total phenolic, flavonoid, proline, and total soluble sugar contents. The results suggest that CK or GA_3_ can reduce waterlogging-induced damage to mung bean and other cash crops (Islam et al., 2022). Thus, GAs enhance plant tolerance to abiotic stress by improving plant metabolism processes, such as regulating membrane permeability, enzymatic activities, osmolytes, and ion uptake.

### Strigolactones

Strigolactones (SLs), an emerging phytohormone class, are derived from carotenoids. Several enzymes located in the cytosol and plastids react with carotenoids to generate SLs in the form of apocarotenoid compounds (Cooper et al., 2018; Hossain et al., 2021). SLs are exuded by 80% of the roots of land plants and have been implicated in the symbiotic relationships of arbuscular mycorrhizae (Cooper et al., 2018; Zwanenburg and Blanco-Ania, 2018). Recent discoveries have reported that SLs can modulate various molecular and physiological processes to adapt to abiotic stresses. However, only a few studies have exploited their influence on antioxidant defense systems in response to abiotic and oxidative stress (Table 1). Therefore, more studies are needed to understand the emerging and protective role of this phytohormone.

Ma et al. (2017) reported that SL (0.18 μM GR24) applied to rapeseed plants under NaCl stress increased growth (mainly shoot and root fresh and dry weights), chlorophyll content (soil and plant values increased by up to 23% compared to the corresponding plants under salinity), and photosynthesis, and reduced oxidative stress by modulating POD and SOD activities and lowering MDA content. Similarly, spraying grapevines under drought stress with SL (GR24; 1, 3, and 5 μM) improved drought tolerance by reducing EL, ROS, MDA, and Chl contents, and IAA and zeatin riboside levels and increasing SOD activity (105% and 90%) and ABA content (Min et al., 2019). Drought stress alone reduced the activity of several other antioxidant defense enzymes. Crosstalk between SLs and other hormones, especially ABA, could be important in GR24-induced drought tolerance (Min et al., 2019). In the same study, the SL treatments mitigated phenotypic damage (such as yellowness and blight) induced by drought stress to varying degrees, particularly in the 5 μM GR24 treatment (Min et al., 2019). In another study, the foliar GR24 treatment enhanced drought tolerance by lowering EL and antioxidant enzyme activities (Sedaghat et al., 2017).

In woodland sage (*Salvia nemorosa*) exposed to environmental salinity, SL treatments (0.1, 0.2, 0.3, and 0.4 μM) reduced the harmful effects of salinity by increasing proline accumulation and decreasing POD, CAT, SOD, and DR activities (Sharifi and Bidabadi, 2020). Exogenous application of SLs also improved plant growth rates, particularly at 0.4 μM SL. Exogenous SL improved the growth and biomass of tea crab apple (*Malus hupehensis* Rehd) seedlings under KCl stress by protecting photosynthetic machinery, regulating osmotic balance by increasing proline, free proteins, and soluble sugar contents, and mitigating oxidative stress by reducing ROS levels by triggering the enzymatic system (POD, CAT) (Zheng et al., 2021). In two barley (*Hordeum vulgare*) genotypes (Cd sensitive and resistant) exposed to 10 mM Cd, 1 mM GR24 reduced Cd accumulation, protected photosynthetic apparatus, increased nutrient uptake, and inhibited ROS (Qiu et al., 2021). In addition, GR24 increased nitric acid (NO) in the Cd-sensitive genotype, which was associated with modulation of the NO signaling pathway, increased SOD and POD activities, augmented AsA and GSH levels, and improved the activity of ascorbate-glutathione cycle enzymes, including APX, GPX, GR, DHAR, and MDHAR (Qiu et al., 2021). Likewise, 10 μM GR24 enhanced the accumulation of carbohydrates and the synthesis of sucrose-related enzyme activities, increased the photosynthetic efficiency and plant biomass, antioxidant enzyme activities, and antioxidant substance contents, and reduced H_2_O_2_ and MDA levels in cucumber (*Cucumis sativus*) seedlings under low light stress (Zhou et al., 2022). Similarly, exogenous SL relieved oxidative damage and photosynthetic inhibition of cucumber seedlings under salt stress through the MAPK cascade pathway (Zhang et al., 2022). Hence, SLs play crucial roles in plant growth and development and in alleviating multiple abiotic stresses.

### Ethylene

Ethylene (ET) is a gaseous phytohormone involved in numerous stages of plant growth and development and a range of physiological, biochemical, and molecular processes, including plant stress responses, seed development, root growth, photosynthesis, flowering, transpiration, stomatal closure, Chl degradation, fruit ripening, flower and leaf senescence, and leaf and petal abscission. ET is biosynthesized from methionine via *S*-adenosyl-L-methionine (*AdoMet*) and the cyclic non-protein amino acid ACC (Khan et al., 2017; Sun et al., 2017; Wang et al., 2017; Husain et al., 2020). ET controls ROS production under several abiotic stresses, including cold, heavy metal, drought, salinity, and waterlogging (Steffens, 2014; Zhang et al., 2016; Chen et al., 2017). ET-reactive transcription is pivotal for regulating ROS biosynthesis and signaling (Steffens, 2014).

Wu et al. (2008) reported that expression of the ET-responsive gene *JERF-3* in tobacco (*Nicotiana tabacum*) plants is induced under redox destabilization, increasing plant tolerance to salinity, drought, and freezing due to the increased expression of genes associated with oxidative stress. Furthermore, induced expression of *JERF-3* enhances SOD activity and reduces ROS accumulation in tobacco plants. Under salinity stress (0.15 M NaCl), root length of tobacco doubled in the overexpression lines compared to wild-type, while osmotic stress (0.2 mM mannitol) increased lateral root growth, including root length (34%). After 20 days of drought stress, more than 85% of the 3-week-old wild-type seedlings had wilted, while the overexpression lines showed normal growth (Wu et al., 2008). Introducing ET-responsive genes linked to antioxidants can improve tolerance to drought-induced oxidative stress. Moreover, Wang et al. (2012) documented an ET-responsive *OsWR1* gene as reactive during drought stress, countering oxidative stress by enhancing wax synthesis in leaves and thus thwarting water loss and oxidative injury in rice. In the same study, 87.8% of wild-type seedlings exhibited drought sensitivity, evidenced by rolled leaves, but only 18.5–28.6% of seedlings from the *OsWR1* overexpression line (*Ox-WR1*) had a drought-responsive phenotype (Wang et al., 2012). Furthermore, where 80% of wild-type seedlings exhibited tolerance, 71.5–78.9% of seedlings from a line with RNA interference silencing *OsWR1* (*RI-WR1*) showed drought-sensitive phenotypes. These results suggest a regulatory role for ET mediated by ET-responsive genes (Wang et al., 2012). In soybean, high temperature improved the ET generation rate and reduced POD, SOD, and CAT activities (Djanaguiraman et al., 2011). In contrast, the application of an ET inhibitor (1-methyl cyclopropane; 1 μg L^–1^) reduced ROS accumulation and enhanced CAT, POD, and SOD activities (Djanaguiraman et al., 2011). Notably, ET regulated the inhibition of membrane lipid peroxidation by reducing O_2_^–^ and H_2_O_2_ contents and improving the antioxidant defense system in soybean under high temperature (Djanaguiraman et al., 2011).

Fine-tuning of osmosis and redox conditions via plant hormones is crucial for plant tolerance to drought. One study showed that ET signaling modulates soluble sugars and proline gathering for adaptation to osmotic stress. In addition, ET-imperfect *Arabidopsis* mutants developed more oxidative stress than wild-type plants and had improved SOD and POD activities, intensifying oxidative stress under drought stress (Cui et al., 2015). The same study showed that ET-imperfect mutants (especially *ein2-5*) displayed hypersensitivity to water stress and decreased survival rate (almost 49%) relative to control plants (nearly 80%). The impact of exogenous ethylene (50 μM ethephon) was evaluated in Madagascar periwinkle (*Catharanthus roseus*) plants under Cd stress. Notably, ET remarkably decreased Cd content in whole plants and reduced MDA and H_2_O_2_ generation in leaves and roots, indicating a link between Cd toxicity tolerance and ET application (Chen et al., 2017).

For wheat seedlings exposed to salinity stress (100 mM NaCl), foliar treatment with 6% glucose and 200 μL ethephon alone or combined revealed that ethephon reduced glucose sensitivity and improved growth and photosynthesis. Furthermore, ET-induced reduced GSH content leads to enhanced expression of *psbA* and *psbB* genes, protecting photosynthetic activities under salinity stress (Sehar et al., 2021). Similarly, Chinese mustard (*Brassica juncea*) under salinity stress (100 mM NaCl) and treated with ethephon (200 μl L^–1^) and sulfur (S) (200 mg kg^–1^) reduced oxidative stress by inhibiting ROS activity and Na^+^ accumulation, which was attributed to ameliorating the activity of enzymes in the ascorbate-glutathione cycle (Fatma et al., 2021). The combined treatment improved thylakoid membranes and photosynthetic rate and reduced ABA storage in stomatal guard cells, increasing stomatal conductance in plants under salinity stress (Fatma et al., 2021). Heat stress increased the accumulation of H_2_O_2_ and thiobarbituric acid reactive substances (TBARS) but decreased starch and sucrose contents and photosynthesis. Exogenously applied ET sourced from ethephon reduced H_2_O_2_ and TBARS contents by increasing the enzymatic antioxidant defense system and improving carbohydrate metabolism, photosynthesis, and plant growth. In addition, the ethephon treatment improved photosynthesis by upregulating *psbA* and *psbB* genes in photosystem II in heat-stressed plants (Gautam et al., 2022). As a result, ET led to abiotic stress tolerance and reduced oxidative stress by inhibiting ROS activity and H_2_O_2_ contents and improving the antioxidant defense system. Further investigations are needed to reveal the antioxidant potential of ET under various abiotic stressors.

## Transgenic and CRISPR/Cas-based solution for crop improvement

**FIGURE 5:**
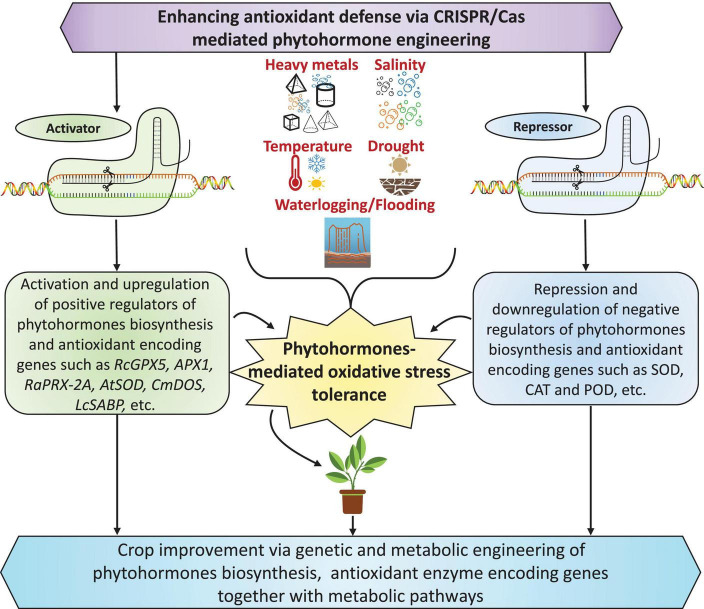
Schematic for improving phytohormone-mediated antioxidant defense via genetic and metabolic engineering using a modern gene-editing tool such as clustered regularly interspaced short palindromic repeats-CRISPR-associated proteins (CRISPR/Cas). Targeted engineering to activate or repress biosynthesis and enzyme-encoding genes can help enhance abiotic-stress-induced oxidative stress in plants by improving the activity of antioxidant defense systems. The genes within the boxes are based on the literature cited in the main text.

Since most crop productivity losses are due to abiotic stresses, research should focus on sustainable crop production by developing broad-spectrum abiotic-stress-tolerant plants. Genetic engineering tools like overexpression or knockdown/knockout via CRISPR/Cas system have recently become successful interventions for increasing crop productivity under challenging environmental conditions. Nevertheless, genetic engineering and transgenic research accomplishments rely on active plant transformation approaches, even for the amalgamation and practical expression of extraneous genes in a plant genome. Lately, scientists have focused on transgenic and gene editing research involving genes encoding antioxidant enzymes and their interaction with/regulation by phytohormone signaling and biosynthesis under various abiotic stresses (Figure 5).

A study by Liu et al. (2020a) discovered that *OsGT-2*, a crucial trihelix transcription factor in rice, significantly contributes to oxidative stress tolerance in rice as a stress-responsive factor. The CRISPR/Cas9-mediated *OsGT*γ*-2* transcript expression indicated *OsGT*γ*-2* transcripts accumulation in crop plants over time before increasing quickly in response to oxidative stress, showing its importance as a potential gene for future research (Liu et al., 2020a). Wang et al. (2022b) studied that transformation of *MruGSTU39* in *M. ruthenica*, and alfalfa improved plant growth and survival under drought stress by upregulating GST and GPX activities to detoxify ROS to reduce membrane damage (Wang et al., 2022b). Likewise, auxin response factors have been functionally characterized under water deficit. Chen et al. (2021b) revealed that the loss of function of SlARF4 (*arf4*) using CRISPR/Cas 9 enhanced plant tolerance to water stress and rehydration ability. In addition, *rf4* mutants had higher contents of antioxidant substances, SOD and CAT activities, and actual PSII photochemical efficiency and ultimately improved the water stress tolerance (Chen et al., 2021b). Another study reported that ABA-drought-ROS 3 (*OsADR3*) conferred drought stress tolerance by enhancing antioxidant defense and regulating *OsGPX1* (Li et al., 2021). The authors showed that CRISPR/Cas9-mediated knockout of *osadr3* increased the sensitivity of rice to drought and oxidative stress. These outcomes suggest that *OsADR3* has a positive regulatory role in drought stress tolerance by prompting antioxidant defense and is related to ABA signaling pathway in rice (Li et al., 2021). To examine the function of *OsPRP1* (proline-rich proteins-PRPs) in cold stress, rice mutant plants were created using CRISPR/Cas9 technology (Nawaz et al., 2019). It was discovered that mutant lines showed decreased antioxidant enzyme activity under cold stress along with down-regulation of the expression of three antioxidant genes (*SOD4, POX1*, and *OsCAT3*) that code for antioxidant enzyme activities. However, the exogenous SA treatment boosted the antioxidant enzyme activity and enhanced cold tolerance (Nawaz et al., 2019).

Overexpression of the alfalfa (*Medicago sativa*) GST gene (*MsGSTU8*) in transgenic tobacco (*Nicotiana tabacum*) plants improved salinity tolerance, mainly by causing GSH to scavenge ROS under stressful environments (Du et al., 2019). *MsGSTU8* expression is also induced by drought, low temperature, salinity, and ABA treatment. Transgenic tobacco plants had significantly enhanced SOD, GST, POD, and CAT activities and reduced EL, MDA content, and ROS accumulation (Du et al., 2019). Likewise, Srivastava et al. (2019) analyzed the rice gene *OsGSTU30* in transgenic *Arabidopsis* under drought and heavy metal (Cr) stress. Overexpression of *OsGSTU30* enhanced tolerance to both stresses relative to control plants and increased GST and GPX activities. In another study, overexpression of the *Rhodiola crenulata* gene *RcGPX5* in transgenic *Salvia miltiorrhiza* enhanced drought and oxidative stress tolerance and decreased MDA production. Transgenic plants had increased GR, APX, and GPX activities and decreased H_2_O_2_ and ${\text{O}}_{2}^{∙-}$ accumulation. Overexpression of *RcGPX5* also improved plant biomass, doubling root dry weight in transgenic plants (Zhang et al., 2019). Similarly, overexpression of the *Arabidopsis* gene *AtGPX5* modulated plant growth, development, and redox biology and enhanced the GSH pool and GPX activity under salinity in transgenic *Arabidopsis* (Riyazuddin et al., 2019). *AtGPX5* overexpression mediated seed growth under salt stress, and the mutants had significantly longer roots and greater lateral root density and fresh weights than the wild-type (Riyazuddin et al., 2019).

Likewise, overexpression of the *Arabidopsis* APX gene (*APX1*) in Chinese mustard (*Brassica juncea*) increased salinity tolerance, the activities of several antioxidant defense systems (APX by 1.9-fold, GPX by 1.5-fold, and POD by 2-fold), proline accumulation, and Chl content (Saxena et al., 2020). *TaPRX-2A* (POD enzymatic gene family) overexpression in transgenic wheat plants improved growth-related attributes and salinity tolerance, which was induced by NaCl, PEG-6000, H_2_O_2_, SA, MeJA, and ABA (Su P. et al., 2020). The overexpressed plants are evidenced by the higher RWC and longer shoots in the transgenic lines compared to CK (Su P. et al., 2020). Transgenic plants had enhanced SOD, POD, and CAT activities, decreasing ROS accumulation and MDA content (Su P. et al., 2020). In transgenic *Arabidopsis* seedlings, overexpression of two SOD genes, *AtSOD* and *CmSOD*, isolated from *Arabidopsis* and pumpkin (*Cucurbita moschata*), respectively, increased tolerance to chilling and oxidative stress (Lin et al., 2019). The seed germination rate in transgenic or WT plants did not significantly differ; however, the leaves of transgenic plants endured the chilling stress and were large, green, and healthy (Lin et al., 2019). Interestingly, transgenic plants increased Chl content and SOD activity and decreased ${\text{O}}_{2}^{∙-}$ accumulation.

Wang et al. (2020) reported that overexpression of the peach (*Prunus persica*) gene *SnRK1* (*PpSnRK1*α) in transgenic tomato plants enhanced salinity tolerance. Notably, transgenic plants had increased ROS metabolism attributable to increased activity of SOD (62–96%) and POD (8–15%) and improved expression of antioxidant-related genes. Li et al. (2019) reported that overexpression of the Chinese wolfberry (*Lycium chinense*) gene *LcSABP* (SA binding protein 2 or *SABP2*, a positive regulator in the SA pathway) in transgenic tobacco plants increased drought tolerance, mainly due to the upregulation of SA content. Stress tolerance in the transgenic plants was mainly attributed to increased Chl content and SOD, POD, and CAT activities and decreased MDA content. The RWC was also higher in the *LcSABP* transgenic lines than in the WT lines. Similarly, overexpression of a gene encoding an ET-responsive TF, *ERF96*, in transgenic *Arabidopsis* increased selenium tolerance, with remarkable increases in CAT, GPX, and GSH activities and reduced overall ROS accumulation (Jiang et al., 2020).

Jasmonic acid-deficient tomato mutant *def-1* exposed to salinity stress (100 mM NaCl) showed a two-fold increase in H_2_O_2_ level along with reduced plant growth parameters, including shoot (3.28 g) and root (0.72 g) dry weights, shoot length (9.04 cm), and leaf number (5.83) under treatment (Abouelsaad and Renault, 2018). Djemal and Khoudi (2022) showed that ethylene-responsive transcription factor *TdSHN1* from durum wheat (*Triticum durum*) conferred Cd, Cu, and Zn tolerances in transgenic yeast and tobacco. The transgenic tobacco lines exhibited more significant biomass accumulation and longer roots, reserved more Chl, and formed less ROS than WT plants. In addition, higher activities of ROS-scavenging enzymes (SOD and CAT) contributed to heavy metal tolerance (Djemal and Khoudi, 2022).

Overall, transgenic and gene-edited studies have made significant progress in recent years. Future investigations should focus on the metabolic engineering of the abovementioned genes, as metabolic and genetic engineering of the related pathways can open new perspectives and expand our knowledge on phytohormone-mediated antioxidant defense responses to abiotic stresses. In this regard, the CRISPR/Cas system is the most promising tool and can provide new avenues for engineering plant hormones and antioxidant biosynthesis genes to confer abiotic stress tolerance in various crop plants. The biosynthetic ways and synergy impacts of hormonal crosstalk are less explored; thus, further investigations are needed to elevate our knowledge and classify innovative genes encoding proteins involved in phytohormone metabolism to develop abiotic stress tolerance in plant species of interest. Recent discoveries have unlocked avenues for genetically engineering plant hormones to confer abiotic stress tolerance.

## Plant hormones and their interactions with neurotransmitters in stress tolerance

**FIGURE 6:**
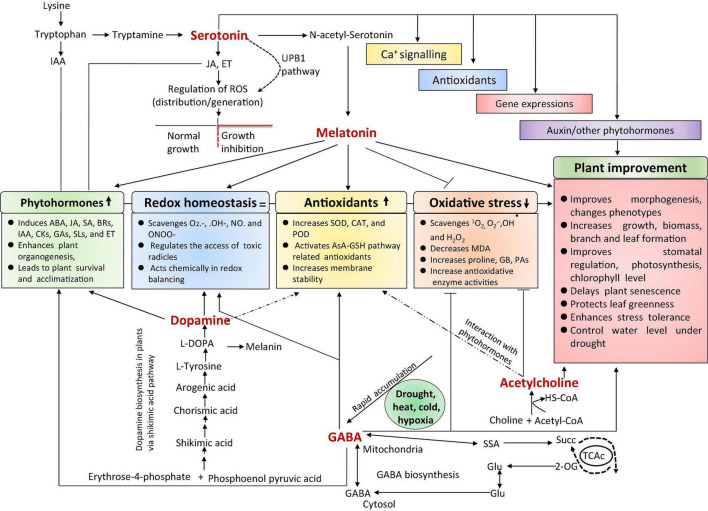
Possible mechanisms for the interaction of neurotransmitters and phytohormones and their roles in several physiological processes. Phytohormones and NTs coordinate as growth regulators, antioxidant activators, redox state managers, and oxidative stress reducers, improving plant growth and enhancing multiple stress tolerances. The illustration presents a series of physiological and biochemical mechanisms linked to the interaction of five NTs with different plant growth regulators. The solid and dashed lines indicate strong and weak interaction, respectively. Lines with bars show the inhibition of a physiological process by NTs. During this process, GABA accumulates rapidly in response to several abiotic stresses. GABA, γ-aminobutyric acid; ABA, abscisic acid; GAs, gibberellic acids; IAA, indole-3-acetic acid; SA, salicylic acid; BRs, brassinosteroids; JS, jasmonic acid; CKs, cytokines; GAs, gibberellic acids; SLs, strigolactones; ET, ethylene; SOD, superoxide dismutase, CAT, catalase; POD, peroxidase; AsA-GSH, ascorbate-glutathione; H_2_O_2_, hydrogen peroxide; ^1^O_2_, singlet oxygen; ${\text{O}}_{2}^{∙-}$, superoxide; OH, hydroxyl radical; MDA, malondialdehyde; GB, glycine betaine; Pas, polyamines; UPB1; UPBEAT1 transcription factor; Glu, glutamate; 2-OG, 2-oxoglutarate; TCAs, tricarboxylic acid cycle; SSA, succinic semialdehyde; Succ, succinate.

Plant neurobiology is fascinating for studying neurological signaling molecules such as NTs and cellular functions. Several NTs such as serotonin (SRT), melatonin (MLT), dopamine (DOPA), acetylcholine (Ach), and γ-aminobutyric acid (GABA) are produced in mammals and other living organisms (Akula and Mukherjee, 2020). Several recent reports highlight NTs in plant development and their interactions with plant hormones, stress adaptation, and tolerance (reviewed by Arnao and Hernández-Ruiz, 2017, 2021). Plant hormones such as ABA, JA, SA, BRs, IAA, CKs, GAs, SLs, and ET interact with NTs to improve morpho-physio-biological responses in normal and stressed plants (Fu et al., 2017; Yang et al., 2019; Abd El-Naby et al., 2020), which activate the antioxidant system and reduce ROS, MDA, and REL to mitigate abiotic stress-induced oxidative stress (Fu et al., 2017; Yang et al., 2019). In the following sections, we discuss the mechanisms involved in NTs and plant hormones that improve plant growth and abiotic stress tolerance, especially related to redox homeostasis (Figure 6).

### Serotonin

5-Hydroxytryptamine (5-HT), also known as serotonin (SRT), is a monoamine NT that acts as an important signaling and stress-reducing molecule. SRT is involved in various morpho-physiological processes, including stress responses, growth, and development (Erland et al., 2019), and has a significant role to develop crosstalk and interaction in conjunction with plant hormones and other associated biomolecules (Mukherjee, 2018). SRT possesses antioxidative and growth-inducing properties, which benefit plant adaptation and acclimatization (Kaur et al., 2015). SRT has less antioxidative potential than other NTs such as MLT. Little attention has been given to exploring the direct involvement of SRT in abiotic stress tolerance in plants, with most SRT studies highlighting its function in shoot organogenesis, root architecture, growth, flowering, senescence, and defense responses (Erland et al., 2019; Abbasi et al., 2020b; Akula and Mukherjee, 2020). However, stress-induced SRT regulates signaling, antioxidants, gene expression, and plant hormones (Abbasi et al., 2020b; Figure 6). Several studies have indicated that SRT modulates the antioxidant activity and ROS scavenging (Hayashi et al., 2016; Thrane et al., 2017; Erland et al., 2019). For example, SRT can reduce the toxic effect of tryptamine by increasing its antioxidant properties (Abbasi et al., 2020b). Senescence-induced SRT biosynthesis of *TDC* overexpression rice lines delayed plant senescence relative to SRT-deficient transgenic rice lines by improving the antioxidant defense system and reducing oxidative damage (Kang et al., 2009). In a recent study, He et al. (2021) reported that exogenous supplementation of SRT maintains osmotic potential by increasing soluble sugars, proline, and soluble proteins and enhancing the expression of *SOD*, *COR6.6*, *COR15*, and *CBFs* genes in rapeseed seedlings in response to cold stress. The overall findings indicate that a low dose (0.03 g L^–1^) of SRT maintains osmotic balance by increasing osmoprotectants and ROS scavenging activity in cells under cold stress (He et al., 2021). SRT also confers defense against biotic stresses (pathogen and herbivores) through its antioxidant properties and induction of cell wall reinforcement (Ramakrishna et al., 2011).

The genes involved in SRT, ET, and isoflavone biosynthesis are interlinked; isoflavone is an antioxidant-improving polyphenol found in several plants, including soybean, faba bean, chickpea, peanut, and other nuts (Thrane et al., 2017). A recent soybean study found that exogenous SRT regulated isoflavone by inducing ET and isoflavone biosynthetic genes (Kumar et al., 2021a,b). Furthermore, a complex interaction between SRT and auxin-responsive genes occurs in plants exposed to abiotic stress (Kaur et al., 2015; Mukherjee, 2018). However, a high SRT concentration (160 μM) did not regulate the auxin response in *Arabidopsis* roots, implying that SRT-mediated lateral root initiation is likely independent of auxin response (Pelagio-Flores et al., 2011).

Plant studies have gained significant insights into SRT-induced plant growth regulation and the interaction of JA, ET, and ROS (Figure 6). In *Arabidopsis*, a high concentration of SRT (300 μM) inhibited primary root growth, affected ROS distribution, and induced JA synthesis (Pelagio-Flores et al., 2016). Indeed, mutations involving the JA and ET pathways culminated in poor SRT sensitivity, inhibiting root growth. Accordingly, the interaction of SRT and JA appears to act as a negative regulator of root growth in *Arabidopsis* (Pelagio-Flores et al., 2016). SRT also has synergistic roles with MLT, increasing biomass production by approximately 80% in soybean under 32°C temperature stress (Kumar et al., 2021a). A study on a rice strain with a high-lysine phenotype demonstrated a link between the metabolic regulation of SRT and induction of the JA response (Yang et al., 2018), suggesting that coordinated events of SRT biosynthesis (induced by tryptophan decarboxylase expression) are linked to the JA pathway in rice grain endosperm. Furthermore, SRT accumulation in rice changed cell wall integrity, reduced biotic stress by acting as a ROS scavenger, and protected uninfected tissue from oxidative stress (Hayashi et al., 2016). The transformation of SRT into MLT was critical for regulating response intensity. Sunflower (*Helianthus annuus*) seedling roots under salt stress accumulated more SRT than MLT (Mukherjee et al., 2014), suggesting that the growth-inhibitory activity of SRT is lost transiently in cells due to its conversion to MLT.

### Melatonin

Melatonin (*N*-acetyl-5-methoxytryptamine; MLT) is an indole derivative synthesized from tryptophan through a biosynthetic pathway in animals and plants (Arnao and Hernández-Ruiz, 2017, 2021; Sharif et al., 2018; Raza et al., 2022d). It is a master regulator that influences many physiological processes in plants. Recent evidence suggests that MLT functions in response to multiple stressors, collaborating with other plant hormones to reduce stress-induced adverse effects, boost antioxidant activity, regulate abiotic stress-induced candidate genes/transcription factors, mitigate oxidative stress, and ultimately improve stress tolerance (Arnao and Hernández-Ruiz, 2021; Sun et al., 2021; Siddiqui et al., 2022; Raza et al., 2022d). The conferring mechanisms of MLT associated with abiotic stress tolerance that ensures redox homeostasis has been well-studied (Arnao and Hernández-Ruiz, 2021; Siddiqui et al., 2022). MLT acts to improve the redox state by scavenging ROS and RNS, such as ${\text{O}}_{2}^{∙-}$, OH^–^, NO, and peroxynitrite (ONOO^–^) (Arnao and Hernández-Ruiz, 2019). The detoxifying potential of MLT controls the access of these harmful radicles and maintains redox hemostasis (Figure 6). In addition, MLT induces the free-radicle-generating enzyme NADPH oxidase (known as Rbohs), generating ${\text{O}}_{2}^{∙-}$ and SOD to increase H_2_O_2_ (Fichman and Mittler, 2020; Arnao and Hernández-Ruiz, 2021). During this regulatory process, MLT also induces the expression of several key enzymes (e.g., CAT, POD, APX, AsR/GR, and Prx) responsible for detoxifying excess H_2_O_2_ (Sharif et al., 2018). Consequently, MLT regulates elements of the AsA-GSH cycle (Siddiqui et al., 2019). Under stress, excess ROS and/or RNS induce the expression of several genes (e.g., *T5H, TDC, COMT*, and *ASMT*) to increase endogenous MLT levels in stressed plants (Arnao and Hernández-Ruiz, 2019). Exogenous supplementation of MLT also counteracts ROS/RNS due to its antioxidant potential, contributing to abiotic stress tolerance in plants (Sharif et al., 2018; Arnao and Hernández-Ruiz, 2019, 2021; Fichman and Mittler, 2020).

In association with its significant role in abiotic stress tolerance in the context of redox homeostasis, MLT can improve plant vegetative growth, mineral content, fruit number, and yield. Exogenous supplementation of MLT (25–50 mg L^–1^) improved heat stress tolerance in orange (*Citrus sinensis*) trees, increasing shoot length (33.3%), chlorophyll content (10%), Fe content (50%), and fruit yield (33.3%) relative to the control (Abd El-Naby et al., 2020). MLT induced transcriptome variation in rapeseed seedlings, where a combined mechanism of candidate genes and plant hormones (CK, JA, and GA3) induced plant growth and protected plants from salt-induced oxidative stress (Tan et al., 2019). Under drought stress, MLT-inducing *TaCOMT* lines (*TaCOMT1, TaCOMT2*) had higher survival rates (75% and 80%) than the wild-type (33.75%) and higher GA_3_ and IAA levels (Yang et al., 2019). Numerous studies have explored the relationship between MLT and several classical plant hormones, including IAA, CKs, ABA, GAs, SA, BRs, and JA, which regulate plant improvement and abiotic stress tolerance. MLT delayed leaf senescence in *Arabidopsis* by downregulating IAA17 (Shi et al., 2015). However, some studies suggest that MLT-induced improved plant growth varies with MLT dose (Park, 2011; Wen et al., 2016; Jahan et al., 2021). For example, a moderate dose of MLT (50 μM) induced advantageous root proliferation in tomatoes, which was linked to IAA regulation. Examples of IAA-related genes and efflux transporters include *PIN1, PIN3, IAA19*, and *IAA24* (Wen et al., 2016). However, high concentrations of MLT (>100 μM) can reduce flowering and inhibit root length and lateral root proliferation (Park, 2011). Siddiqui et al. (2022) reported that MLT reduces the toxic effect of Cd by interacting with mineral nutrition, such as potassium, which modulates the activity of fructose-1,6-bisphosphatase and sedoheptulose-1,7-bisphosphatase, increasing photosynthetic efficiency, carbon assimilation in tomato under Cd stress. MLT also has protective roles related to leaf greenness, stress tolerance, and delayed senescence (Park, 2011), all of which are likely to relate to the CK response. Furthermore, MLT conferred heat stress tolerance and suppressed heat-induced leaf senescence in tomatoes by altering ABA and GA contents (Jahan et al., 2021). In this context, MLT supplementation reduced leaf yellowing, improved Fv/Fm ratio, reduced ROS generation, and induced the expression of *Rbohs*, chlorophyll catabolic genes, and senescence-associated genes (Jahan et al., 2021). Another study reported that MLT inhibited heat-induced leaf senescence in perennial ryegrass (*Lolium perenne*), which involved CK and ABA biosynthesis (Zhang et al., 2017). In a recent study, MLT application enhanced drought tolerance in maize by reducing the oxidative damage caused by excessive ROS production (Alharby and Fahad, 2020). MLT also maintained osmolyte accumulation, α-amylase activity, antioxidant enzyme activities, and photosynthesis systems in maize under drought stress (Alharby and Fahad, 2020).

The ABA hormone is an important endogenous messenger in plant responses to abiotic stresses and is required for fine-tuning plant growth and development. According to recent research, ABA can interact with MLT during abiotic stress exposure (Arnao and Hernández-Ruiz, 2017, 2021). Figure 6 illustrates existing data on the interactions between MLT and plant hormones and how MLT regulates or intervenes in plant hormone signaling and responses following stress exposure. In *Elymus nutans* grass under cold stress, exogenous MLT increased endogenous MLT and ABA contents, activating cold-responsive genes, inducing the antioxidant system, and improving cold tolerance (Fu et al., 2017). Furthermore, MLT protected TaCOMT *Arabidopsis* from drought by coordinating GAs, IAA, and ABA and increasing SOD, CAT, APX, and GR activities (Yang et al., 2019). A combined effect of MLT, GAs, and SA mitigated heat stress in orange trees (*Citrus sinensis*) (Abd El-Naby et al., 2020). In rapeseed seedlings, exogenous MLT regulated BR, GAs, and JA and the expression of GA synthesis genes *GA20ox, GA3ox, GID1, COMT, POD*, and *UGT*, improving hormone use efficiency, plant phenotype, and salt stress adaptation (Tan et al., 2019). Collectively, these studies indicate that MLT plays an essential role as a bio-stimulant in stress responses, interacting with plant hormones to achieve the beneficial effect of increasing tolerance to multiple environmental stressors.

### Dopamine

Dopamine (DOPA), norepinephrine, and epinephrine are well-known biogenic amines and NTs. DOPA, also called 3-hydroxytyramine and 3,4-dihydroxyphenethylamine, is an important NT in mammals and plants (Akula and Mukherjee, 2020), which interact with plant hormones (Liu et al., 2020b; Figure 6). For example, DOPA levels in potatoes (*Solanum tuberosum*) increased significantly in response to drought, UV, and ABA (Widrych et al., 2004). ABA, salt, and drought exposure induced norepinephrine in the same plants. Notably, exogenous DOPA induces its endogenous counterpart to regulate gene expression and reduce the adverse effects of drought, salt, and disease on plant physiological processes (Liu et al., 2020b). Akula and Mukherjee (2020) describe a mechanism associated with DOPA involvement in redox signaling, induction of antioxidant potential, and oxidative stress modulation, regulating plant survival with abiotic or biotic stress tolerance. Plants generate various redox chemicals (e.g., ${\text{O}}_{2}^{∙-}$, OH^–^, and H_2_O_2_) during physiological processes that can interact with DOPA (Bamel and Prabhavathi, 2020). During these processes, DOPA induces key enzymes (CAT, SOD, and POD) that reduce ROS levels in plants (Gomes et al., 2014; Figure 6). DOPA has antioxidant potential due to the presence of the hydroxyl (OH) group in the phenolic ring that supports the detoxification of dangerous ROS (Bamel and Prabhavathi, 2020). DOPA treatment inhibits IAA oxidase and thus root growth (root length, fresh weight, and dry weight). Furthermore, DOPA decreases cell viability and PAL and POD activities and decreases SOD activity; these modifications were attributed to higher auxin production in soybean roots (Guidotti et al., 2013). However, during the oxidation stress modulation process, tyrosinase oxidizes DOPA to produce dopaminoquinone, semiquinones, and quinones in a series of autoxidation processes that result in ROS production (Bamel and Prabhavathi, 2020). In contrast, DOPA can alleviate oxidative stress by enhancing AsA-GSH cycle-related enzymes (Akula and Mukherjee, 2020). DOPA also induces lignin-forming candidates with a lower level of H_2_O_2_ production in roots (Soares et al., 2007). Exogenous supplementation of DOPA enhanced drought tolerance in apple trees (*Malus pumila*) by regulating transcription factor WRKY, ethylene response factor ERF, and NAC (Gao et al., 2020a). Exogenous DOPA (0.1 mM) alleviated alkali stress (pH 9.0) in apple seedlings, significantly enhancing plant growth (66%) with vigorous phenotypes, fresh weight (45%), and net photosynthesis (two-fold) relative to untreated seedlings (Jiao et al., 2019).

Dopamine acts as a water-soluble antioxidant; a recent study reported that exogenous DOPA supplementation (100 μM) to watermelon improved seedling health and alleviated chilling stress by regulating proline content, SOD, POD, and CAT activities, and polyamine (PA) metabolism (Jiao et al., 2021). In addition, exogenous DOPA application significantly increased the activities of CAT (∼2-fold), SOD (50%), and POD (2.2-fold) and reduced H_2_O_2_ accumulation (∼30%), MDA content (15%), and EL (20%) relative to untreated plants (Jiao et al., 2019). Likewise, exogenous DOPA (100 μM) supplied to apple trees improved drought tolerance and positively regulated nutrient uptake, transport, and growth (Liang et al., 2018). In the same study, DOPA improved photosynthesis, chlorophyll level, and stomatal function by mitigating the inhibition of plant growth. In another study, DOPA induced an anti-senescence response that regulated nutrient uptake, transport, and restoration, influencing overall plant growth (Bamel and Prabhavathi, 2020). Furthermore, DOPA regulates the genes involved in DOPA biosynthesis and metabolism, leaf senescence, carbohydrate metabolism, SOS pathway, nitrate transporters, antioxidants (cAPX, cGR, MDHAR, DHAR-1), aquaporine (OsPIP), and IAA (IAA oxidase) (Liu et al., 2020b), improving plant adaptability and tolerance to stressors. Ultimately, DOPA is involved in diverse stress responses, physio-biological functions, and plant growth and development.

### Acetylcholine

Acetylcholine (Ach) is an important NT of the neuromuscular junction in vertebrates (Colombo and Francolini, 2019). The presence of Ach has been reported in several taxonomic groups in the plant kingdom (Roychoudhury, 2020), indicating that Ach and/or its associated molecules have a significant role in the response of plant communities to environmental factors. However, Ach research in plant systems is underrepresented compared to its animal counterparts. One study showed that Ach molecules promote normal growth and physiology and mitigate various stresses (Roychoudhury, 2020). Recent reports have revealed the role of Ach in abiotic stress tolerance (Qin et al., 2019, 2020; Su Y. et al., 2020; Tanveer and Shabala, 2020). For instance, Ach regulates ion transport candidate proteins (NHX, AKT1, and HKT1), balancing low Na^+^ and high K^+^ elements (Qin et al., 2019). Furthermore, Ach regulates electrical signaling in plants, and H^+^-ATPase blockers inhibited ATPase activity in Characeae cells. Whereas high Ach supplementation (1 and 5 mM) increased membrane depolarization and regulated Ca^2+^ concentration, indicating the response of Ach, membrane potential, and elemental concentration (Kisnieriene et al., 2012). Like Ach, choline acts as an NT and precursor of Ach biosynthesis (Figure 6). Choline supplementation enhanced the K^+^/Na^+^ ratio in holophytic paspalum by increasing K^+^ and Ca^2+^ concentrations and decreasing Na^+^ and Ca^2+^ (Gao et al., 2020b). In addition, choline interacted in a slow vacuolar channel where it is encoded by *TPC1* genes, trapping Na^+^ in a vacuole, leading to Na^+^ sequestration and osmotic balance in quinoa (*Chenopodium quinoa*) (Pottosin et al., 2014).

Acetylcholine interacts with plant hormones such as IAA, ET, and GAs, boosting antioxidants, osmoprotectants, and stress response genes (Figure 6) to impact plant growth and development (Di Sansebastiano et al., 2014). In tobacco (*Nicotiana tabacum*), Ach reduced Cd stress by increasing plant growth and the activities of SOD, CAT, APX, and GR, glutathione (GSH), non-protein thiols, and phytochelatins (PCs), and modulating Cd distribution in vacuoles and cell walls (Su Y. et al., 2020). In a recent study, Ach interacted with various plant hormones in several plants to ameliorate several environmental constraints and abiotic stimuli (Roychoudhury, 2020). In benth (*Nicotiana benthamiana*), exogenous Ach (10 μM) improved salt tolerance by increasing POD, SOD, soluble sugar, and proline accumulation (Qin et al., 2019). The same group later reported that Ach alleviated salinity (150 mM NaCl) stress in hydroponically grown benth by improving photosynthetic efficiency (48.1–67.9%), SOD (23.7–37.2%), POD (27.2–54.2%), proline (3.43-fold), and soluble sugars (32.4–33.9%), reducing ROS accumulation, chlorosis, and lipid peroxidation, and upregulating the genes *HEMA1, CHLH, CAO*, and *POR* (Qin et al., 2020). Roychoudhury (2020) reported that Ach rescues plants from abiotic stress-induced oxidative stress. For instance, Ach mitigated salt stress by regulating hormones, metabolites, Chl metabolism, photosynthesis, water balancing, and elimination of excess salt in tobacco root cells under salinity (Qin et al., 2020). Ach mitigated oxidative stress in tobacco under Cd stress by regulating enzymatic and non-enzymatic antioxidants (Su Y. et al., 2020). Ach also alleviated osmotic stress in soybean by enhancing plant growth, root and shoot weights, and total dry mass (Braga et al., 2017). *Salicornia europaea* is a strong halophyte that can survive high NaCl (over 3%) through ionic balance; Yamamoto et al. (2009) reported that acetylcholinesterase (AChE) activity increased in *S. europaea* roots and lower stem parts in response to saline conditions. Ach is emerging as a new area of plant research compared to other NTs; there is much to learn about its beneficial effects and interactions with plant hormones in response to various environmental stresses.

### γ-Aminobutyric acid

γ-Aminobutyric acid (GABA) is a ubiquitous four-carbon non-protein amino acid found in many living organisms, including plants (Akula and Mukherjee, 2020). In animals, GABA acts as an inhibitory NT. In plants, GABA accumulates quickly in response to drought, heat, cold, hypoxia, wounding due to herbivory, and infection (Bown and Shelp, 2016). GABA mitigates the adverse effects of stress by regulating plant signaling, hormone biosynthesis, osmoprotectants, and the antioxidative system (Podlešáková et al., 2019). Thus, endogenous GABA likely accumulates in response to abiotic and abiotic stresses. Exogenous GABA spray to muskmelon leaves enhanced abiotic stress (salinity/alkalinity) tolerance by maintaining redox homeostasis and chlorophyll biosynthesis (Jin et al., 2019; Figure 6). It also induced the free-radicle-generating enzyme (respiratory burst oxidase homolog D) and H_2_O_2_ accumulation, reducing H_2_O_2_ concentration by elevating SOD activity, and increased leaf Chl content by inducing the δ-aminolevulinic acid (*ALA*) gene (Jin et al., 2019). In completely wilted gad1/2 *Arabidopsis* mutant plants following drought exposure, a GABA-depleted *gad1/2* mutant showed greater sensitivity to drought and higher stomatal conductivity, with increased stomatal density (15%) and pore width (19.3%), than the WT (Bown and Shelp, 2016). However, the functionally complemented *gad1/2* × *gaba-t* mutant had more GABA than the WT, which significantly reversed these phenotypic and physiological characteristics in response to drought stress (Mekonnen et al., 2016). Exogenous GABA enhanced heat stress tolerance in sunflower by regulating plant growth, yield, antioxidant defense, and stress-responsive candidate genes, increasing proline (∼33.3%), total soluble sugars (16.6%), activities of SOD (1.25-fold), CAT (50%), GR (15%), and MDAR (16%), and gene expression, including *HSP, dehydrin, osmotin*, and *aquaporin* (Abdel Razik et al., 2021).

Interactions between GABA and plant hormones, along with other metabolites, have been studied extensively in various plant systems (Podlešáková et al., 2019). For instance, exogenous GABA supplementation in citrus significantly increased ABA, JA, SA, and IAA levels (Hijaz et al., 2018) and regulated salinity-responsive genes through ABA and ET signaling pathways in poplar (Ji et al., 2018; Figure 6), improving SOD and POD responses, regulating H_2_O_2_, and mediating the interaction of ABA, ET, and other plant hormones that in turn mediated the salt stress response. In addition, GABA levels during abiotic stress exposure can be related to auxin and other metabolites levels (Podlešáková et al., 2019). Plant root growth and drought tolerance have been linked to GABA accumulation and changes in plant hormones (IAA and JA) and other metabolites (L-arginine, spermidine), increasing antioxidant activity and plant survival (De Diego et al., 2015).

**FIGURE 7:**
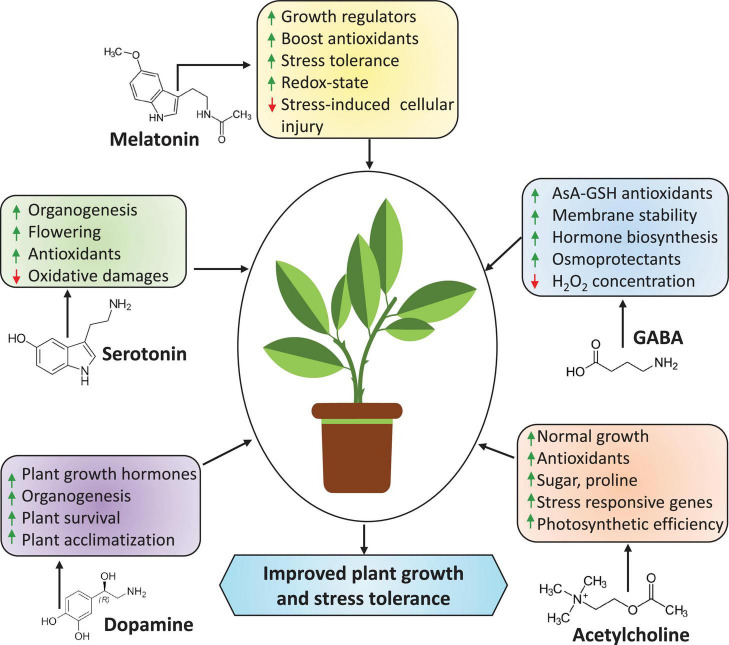
Physiological role of neurotransmitters (NTs) associated with plant growth and stress tolerance in plants. NTs coordinate the regulation of plant growth, development, and adaptation/acclimatization, increasing plant stress tolerance. Up/down arrows indicate the increase/decrease of a physiological parameter. GABA, γ-aminobutyric acid; AsA-GSH, ascorbate-glutathione cycle.

A functional link between GABA and CKs was demonstrated in transgenic barley plants, wherein overexpression of GABA-related genes (*GAD* and *ALMT*) influenced CKs to modify root architecture and aid drought stress avoidance (Pospíšilová et al., 2016). The transgenic barley had improved water content, growth, and yield parameters. In muskmelon (*Cucumis melo*), exogenous GABA improved combined salinity–alkalinity tolerance by regulating AsA, GSH, and chlorophyll synthesis precursors (*Glu, ALA, PBG, URO III*, *Mg-proto IX, Proto IX*, and *Pchl*), increasing Chl content (Jin et al., 2019). GABA also interacts with polyamines (PAs), improving plant stress tolerance by regulating stress-induced candidate gene expression (Podlešáková et al., 2019). These findings support the interaction of GABA with plant growth regulators, indicating its potential role in plant stress tolerance. However, NTs, their interactions with plant hormones, and their involvement in the aforementioned research offer a clear picture of plant improvements. All these studies showed that the interaction of NTs with plant hormones plays a vital role in stress tolerance and crop improvement. We also explored how five important NTs improve plant development, growth, and stress tolerance (Figure 7), which will help understand the significance of NTs in plant research.

## Conclusion and future directions

Worldwide, agricultural production is increasingly being curtailed due to unfavorable growth conditions imposed by diverse environmental stresses. Plant hormones play dynamic roles in arbitrating cellular homeostasis and improving plant health, and their ability to regulate numerous responses is an optimistic and dynamic arena for abiotic stress research. According to the reviewed literature, exogenous and endogenous plant hormones modulate antioxidant defense systems under oxidative and abiotic stresses. Moreover, plant hormones interact with NTs that regulate plant antioxidative mechanisms to relieve oxidative stress-induced injuries. Exploiting these mechanisms is multifaceted because the molecules work as secondary messengers to initiate antioxidants. While plants supplemented with exogenous hormone(s) exhibit improved antioxidant activity, ROS overproduction can still occur and be sustained. Specifically, hormones can induce numerous stress-related genes, and stimulating these genes can induce ROS, which can induce stress genes in a complex biochemical mechanism. Furthermore, various biochemical mechanisms convey stress signaling and can be initiated by exogenously applied plant hormones. Ultimately, exogenous plant hormones can temporarily reduce oxidative stress at a functional level, working as an acclimatization mechanism to increase antioxidant activities and aid in the biosynthesis of defensive compounds. Conversely, frequent or continued supply at higher concentrations can permanently disrupt plant metabolism. Nonetheless, the exogenous application of plant hormones has considerable potential to improve plant stress tolerance and reduce oxidative damage by regulating enzymatic and non-enzymatic antioxidant defense systems and the associated modulation of ROS levels. Recent progress in molecular and genetic tools has improved plant stress tolerance by developing transgenic lines with high antioxidant enzyme activities. In particular, overexpression of genes encoding antioxidant enzymes positively affects abiotic stress tolerance and increases the activities of encoded enzymes. Accurate identification and reporting of multiple candidate genes are needed to enhance the tolerance and yields of transgenic plants under stressful environments.

Whether plant hormones can be used to compensate for the stress-induced modulation of defense-related genes or signaling apparatuses and the actual mechanisms responsible for stress tolerance are unknown. Here, we revealed a mechanism involving several key NTs and plant hormones in several physiological processes and identified their role as a growth regulator, antioxidant activator, redox state manager, and oxidative stress reducer to improve plant growth and enhance multiple stress tolerances (Figure 6). Additionally, these studies combine several models related to each NTs’ physiological involvement in enhancing plant growth, development, and stress tolerance. The five key NTs interact with plant hormones and are involved in several physiological processes, including organogenesis, flowering, photosynthetic efficiency, antioxidant and osmoregulator regulation, and photosynthetic efficiency (Figure 7). Despite the advances in NT research in plants, several areas require attention in future studies. Applying NTs in agriculture and plant acclimatization will reveal more potential benefits. Identifying NT receptors and screening metabolically associated NTs involved in one or more pathways in plants can help improve overall plant health under stress conditions. Modulating these biomolecules in transgenic plants is important to understand their exact mechanism and action. Investigating NTs in plants and their interaction with endophytes would also be worthwhile. Little is known about how NTs affect genetic and epigenetic controls; thus, studies on NT derivatives could reveal promising insights into the epigenetic regulations induced by NTs under stressed plants. Furthermore, in an applied sense, applying NTs as biostimulants would benefit the plant, soil and environments. A cutting-edge metabolome approach is needed to investigate the stress-induced network of novel plant hormones, interactions between newly discovered plant NTs and plant hormones, their pleiotropic responses, and other possible interventions in the plant stress response. Thus, there is the possibility for research focusing on biosynthesis genes and physiological progressions in stress environments. Furthermore, the GE of phytohormone-mediated antioxidant biosynthesis and metabolic pathways may provide new evidence for stress tolerance mechanisms. The competence of plant hormones to induce multiple antioxidant pathways can be exploited in response to oxidative stress. Omics tools, such as genomics, transcriptomics, proteomics, and metabolomics, can be used to identify candidate genes, proteins, and metabolites associated with phytohormonal and NTs biosynthesis pathways. A significant challenge is the production of hormone-engineered plants and genetic manipulation of NT levels or receptors to enhance plant resistance to environmental stresses. Future investigations should use biotechnological tools such as CRISPR/Cas systems to develop gene-edited future plants that can withstand stressful field environments. Speed breeding has recently emerged as a powerful tool for boosting plant growth and development under anticipated environments. CRISPR/Cas could be coupled with speed breeding to develop transgenic plants that can feed millions and enhance global food security.

## Author contributions

AR conceived the idea and designed the table and figures. AR, HS, MAR, ZZ, MMH, MA, SC, SN-K, KHMS, and YZ contributed to the writing and literature search. AR, MA, HSO, KHMS, WZ, and YZ edited and revised the manuscript. All authors have read and approved the final version of the manuscript.

## Footnotes

1
https://www.fao.org/sustainable-development-goals/goals/goal-2/en/

2
https://www.un.org/sustainabledevelopment/hunger/

## References

[B1] Abbas T. Fan R. Hussain S. Sattar A. Khalid S. Butt M. (2022). Protective effect of jasmonic acid and potassium against cadmium stress in peas (*Pisum sativum* L.). *Saudi J. Biol. Sci.* 29 2626–2633. 10.1016/j.sjbs.2021.12.051 35531166 PMC9073065

[B2] Abbasi B. H. Ullah M. A. Nadeem M. Tungmunnithum D. Hano C. (2020a). Exogenous application of salicylic acid and gibberellic acid on biomass accumulation, antioxidant and anti-inflammatory secondary metabolites production in multiple shoot culture of *Ajuga integrifolia* Buch. Ham. ex D. Don. *Indus. Crops Prod.* 145:112098. 10.1016/j.indcrop.2020.112098

[B3] Abbasi B. H. Younas M. Anjum S. Ahmad N. Ali M. Fazal H. (2020b). “Serotonin in plant signalling and communication,” in *Neurotransmitters in Plant Signaling and Communication*, eds Baluška F. Mukherjee S. Ramakrishna A. (Cham: Springer International Publishing), 75–92. 10.1007/978-3-030-54478-2_4

[B4] Abd El-Naby S. K. M. Abdelkhalek A. Baiea M. H. M. Amin O. A. (2020). Mitigation of heat stress effects on washington navel orange by using melatonin, gibberellin and salicylic treatments. *Plant Arch.* 20 3523–3534.

[B5] Abdel Latef A. A. H. Akter A. Tahjib-Ul-Arif M. (2021a). Foliar application of auxin or cytokinin can confer salinity stress tolerance in *Vicia faba* L. *Agronomy* 11:790. 10.3390/agronomy11040790

[B6] Abdel Latef A. A. H. Tahjib-Ul-Arif M. Rhaman M. S. (2021b). Exogenous auxin-mediated salt stress alleviation in faba bean (*Vicia faba* L.). *Agronomy* 11:547. 10.3390/agronomy11030547

[B7] Abdel Razik E. S. Alharbi B. M. Pirzadah T. B. Alnusairi G. S. H. Soliman M. H. Hakeem K. R. (2021). γ-Aminobutyric acid (GABA) mitigates drought and heat stress in sunflower (*Helianthus annuus* L.) by regulating its physiological, biochemical and molecular pathways. *Physiol. Plantarum* 172 505–527. 10.1111/ppl.13216 32979274

[B8] Abouelsaad I. Renault S. (2018). Enhanced oxidative stress in the jasmonic acid-deficient tomato mutant def-1 exposed to NaCl stress. *J. Plant Physiol.* 226 136–144. 10.1016/j.jplph.2018.04.009 29758378

[B9] Ahanger M. A. Akram N. A. Ashraf M. Alyemeni M. N. Wijaya L. Ahmad P. (2017). Plant responses to environmental stresses from gene to biotechnology. *AoB Plants* 9:plx025. 10.1093/aobpla/plx025 28775828 PMC5534019

[B10] Akula R. Mukherjee S. (2020). New insights on neurotransmitters signaling mechanisms in plants. *Plant Signal. Behav.* 15:1737450. 10.1080/15592324.2020.1737450 32375557 PMC8570756

[B11] Alam M. M. Nahar K. Hasanuzzaman M. Fujita M. (2014). Exogenous jasmonic acid modulates the physiology, antioxidant defense and glyoxalase systems in imparting drought stress tolerance in different *Brassica* species. *Plant Biotechnol. Rep.* 8 279–293. 10.1007/s11816-014-0321-8

[B12] Alamri S. Hu Y. Mukherjee S. Aftab T. Fahad S. Raza A. (2020). Silicon-induced postponement of leaf senescence is accompanied by modulation of antioxidative defense and ion homeostasis in mustard (*Brassica juncea*) seedlings exposed to salinity and drought stress. *Plant Physiol. Biochem.* 157 47–59. 10.1016/j.plaphy.2020.09.038 33075710

[B13] Alamri S. Siddiqui M. H. Kushwaha B. K. Singh V. P. Ali H. M. (2021). Mitigation of arsenate toxicity by indole-3-acetic acid in brinjal roots: plausible association with endogenous hydrogen peroxide. *J. Hazard. Mater.* 405:124336. 10.1016/j.jhazmat.2020.124336 33153795

[B14] Alharby H. F. Fahad S. (2020). Melatonin application enhances biochar efficiency for drought tolerance in maize varieties: modifications in physio-biochemical machinery. *Agronomy J.* 112 2826–2847. 10.1002/agj2.20263

[B15] Ali A. Y. A. Ibrahim M. E. H. Zhou G. Nimir N. E. A. Elsiddig A. M. I. Jiao X. (2021). Gibberellic acid and nitrogen efficiently protect early seedlings growth stage from salt stress damage in Sorghum. *Sci. Rep.* 11:6672. 10.1038/s41598-021-84713-9 33758238 PMC7988071

[B16] Álvarez-Méndez S. J. Urbano-Gálvez A. Mahouachi J. (2022). Mitigation of salt stress damages in *Carica papaya* L. seedlings through exogenous pretreatments of gibberellic acid and proline. *Chilean J. Agricult. Res.* 82 167–176. 10.4067/S0718-58392022000100167 27315006

[B17] Al-Zahrani H. S. Alharby H. F. Fahad S. (2022). Antioxidative defense system, hormones, and metabolite accumulation in different plant parts of two contrasting rice cultivars as influenced by plant growth regulators under heat stress. *Front. Plant Sci.* 13:911846. 10.3389/fpls.2022.911846 35712584 PMC9196032

[B18] Amanullah, Ilyas M. Nabi H. Khalid S. Ahmad M. Muhammad A. (2021). Integrated foliar nutrients application improve wheat (*Triticum aestivum* L.) productivity under calcareous soils in drylands. *Commun. Soil Sci. Plant Anal.* 52 2748–2766. 10.1080/00103624.2021.1956521

[B19] Arnao M. B. Hernández-Ruiz J. (2017). Melatonin and its relationship to plant hormones. *Ann. Bot.* 121 195–207. 10.1093/aob/mcx114 29069281 PMC5808790

[B20] Arnao M. B. Hernández-Ruiz J. (2019). Melatonin and reactive oxygen and nitrogen species: a model for the plant redox network. *Melatonin Res.* 2 152–168. 10.32794/11250036 11250036

[B21] Arnao M. B. Hernández-Ruiz J. (2021). Melatonin as a regulatory hub of plant hormone levels and action in stress situations. *Plant Biol.* 23 7–19. 10.1111/plb.13202 33098247

[B22] Avalbaev A. Allagulova C. Maslennikova D. Fedorova K. Shakirova F. (2021). Methyl jasmonate and cytokinin mitigate the salinity-induced oxidative injury in wheat seedlings. *J. Plant Growth Regul.* 40 1741–1752. 10.1007/s00344-020-10221-1

[B23] Azzam C. R. Zaki S.-N. S. Bamagoos A. A. Rady M. M. Alharby H. F. (2022). Soaking Maize Seeds in Zeatin-Type Cytokinin Biostimulators improves salt tolerance by enhancing the antioxidant system and photosynthetic efficiency. *Plants* 11:1004. 10.3390/plants11081004 35448732 PMC9032616

[B24] Bahar N. H. Lo M. Sanjaya M. Van Vianen J. Alexander P. Ickowitz A. (2020). Meeting the food security challenge for nine billion people in 2050: What impact on forests? *Glob. Environ. Change* 62:102056. 10.1016/j.gloenvcha.2020.102056

[B25] Bamel K. Prabhavathi (2020). “Dopamine in Plant Development and Redox Signaling,” in *Neurotransmitters in Plant Signaling and Communication*, eds Baluška F. Mukherjee S. Ramakrishna A. (Cham: Springer International Publishing), 123–139. 10.1007/978-3-030-54478-2_7

[B26] Bashri G. Prasad S. M. (2016). Exogenous IAA differentially affects growth, oxidative stress and antioxidants system in Cd stressed *Trigonella foenum-graecum* L. seedlings: toxicity alleviation by up-regulation of ascorbate-glutathione cycle. *Ecotoxicol. Environ. Saf.* 132 329–338. 10.1016/j.ecoenv.2016.06.015 27344401

[B27] Bashri G. Singh S. Prasad S. M. Ansari M. J. Usmani S. Alfarraj S. (2021). Kinetin mitigates Cd-induced damagesto growth, photosynthesis and PS II photochemistry of *Trigonella* seedlings by up-regulating ascorbate-glutathione cycle. *PLoS One* 16:e0249230. 10.1371/journal.pone.0249230 34157031 PMC8219128

[B28] Basit F. Bhat J. A. Dong Z. Mou Q. Zhu X. Wang Y. (2022a). Chromium toxicity induced oxidative damage in two rice cultivars and its mitigation through external supplementation of brassinosteroids and spermine. *Chemosphere* 302 134423. 10.1016/j.chemosphere.2022.134423 35430206

[B29] Basit F. Liu J. An J. Chen M. He C. Zhu X. (2022b). Seed priming with brassinosteroids alleviates aluminum toxicity in rice via improving antioxidant defense system and suppressing aluminum uptake. *Environ. Sci. Pollut. Res.* 29 10183–10197. 10.1007/s11356-021-16209-y 34515933

[B30] Biareh V. Shekari F. Sayfzadeh S. Zakerin H. Hadidi E. Beltrão J. G. T. (2022). Physiological and qualitative response of Cucurbita pepo L. to salicylic acid under controlled water stress conditions. *Horticulturae* 8:79. 10.3390/horticulturae8010079

[B31] Blakeslee J. J. Spatola Rossi T. Kriechbaumer V. (2019). Auxin biosynthesis: spatial regulation and adaptation to stress. *J. Exp. Bot.* 70 5041–5049. 10.1093/jxb/erz283 31198972

[B32] Bousba R. Rached-Kanouni M. Benghersallah N. Djekoune A. Ykhlef N. (2020). Role of exogenous application of abscisic acid ABA in drought tolerance and evaluation of antioxidant activity in durum wheat genotypes. *Acta Sci. Nat.* 7 44–60. 10.2478/asn-2020-0019

[B33] Bown A. W. Shelp B. J. (2016). Plant GABA: not just a metabolite. *Trends Plant Sci.* 21 811–813. 10.1016/j.tplants.2016.08.001 27542324

[B34] Braga I. Pissolato M. D. Souza G. M. (2017). Mitigating effects of acetylcholine supply on soybean seed germination under osmotic stress. *Braz. J. Bot.* 40 617–624. 10.1007/s40415-017-0367-2

[B35] Brumos J. Robles L. M. Yun J. Vu T. C. Jackson S. Alonso J. M. (2018). Local auxin biosynthesis is a key regulator of plant development. *Develop. Cell* 47 306.–318. 10.1016/j.devcel.2018.09.022 30415657

[B36] Bukhari S. A. B. H. Lalarukh I. Amjad S. F. Mansoora N. Naz M. Naeem M. (2021). Drought stress alleviation by potassium-nitrate-containing chitosan/montmorillonite microparticles confers changes in *Spinacia oleracea* L. *Sustainability* 13:9903. 10.3390/su13179903

[B37] Cai B. Wang H. Liu T. Zhuang W. Wang Z. Qu S. (2019). Effects of gibberellins A4 on budbreak, antioxidant enzymes’ activity and proline content of flower buds in sweet cherry (*Prunus avium*). *Acta Physiol. Plant* 41:88. 10.1007/s11738-019-2876-z

[B38] Cao X. Wu L. Wu M. Zhu C. Jin Q. Zhang J. (2021). Abscisic acid mediated proline biosynthesis and antioxidant ability in roots of two different rice genotypes under hypoxic stress. *BMC Plant Biol.* 20:198. 10.1186/s12870-020-02414-3 32384870 PMC7206686

[B39] Castañeda-Murillo C. C. Rojas-Ortiz J. G. Sánchez-Reinoso A. D. Chávez-Arias C. C. Restrepo-Díaz H. (2022). Foliar brassinosteroid analogue (DI-31) sprays increase drought tolerance by improving plant growth and photosynthetic efficiency in lulo plants. *Heliyon* 8:e08977. 10.1016/j.heliyon.2022.e08977 35243095 PMC8873547

[B40] Chang Z. Liu Y. Dong H. Teng K. Han L. Zhang X. (2016). Effects of cytokinin and nitrogen on drought tolerance of creeping bentgrass. *PLoS One* 11:e0154005. 10.1371/journal.pone.0154005 27099963 PMC4839601

[B41] Chen J. Miao W. Fei K. Shen H. Zhou Y. Shen Y. (2021a). Jasmonates alleviate the harm of high-temperature stress during anthesis to stigma vitality of photothermosensitive genetic male sterile rice lines. *Front. Plant Sci.* 12:634959. 10.3389/fpls.2021.634959 33854518 PMC8039518

[B42] Chen M. Zhu X. Liu X. Wu C. Yu C. Hu G. (2021b). Knockout of auxin response factor SlARF4 improves tomato resistance to water deficit. *Int. J. Mol. Sci.* 22:3347. 10.3390/ijms22073347 33805879 PMC8037468

[B43] Chen J. Xu Y. Fei K. Wang R. He J. Fu L. (2020). Physiological mechanism underlying the effect of high temperature during anthesis on spikelet-opening of photo-thermosensitive genic male sterile rice lines. *Sci. Rep.* 10:2210. 10.1038/s41598-020-59183-0 32042005 PMC7010791

[B44] Chen J. Yan Z. Li X. (2014). Effect of methyl jasmonate on cadmium uptake and antioxidative capacity in *Kandelia obovata* seedlings under cadmium stress. *Ecotoxicol. Environ. Saf.* 104 349–356. 10.1016/j.ecoenv.2014.01.022 24736025

[B45] Chen Q. Wu K. Tang Z. Guo Q. Guo X. Wang H. (2017). Exogenous ethylene enhanced the cadmium resistance and changed the alkaloid biosynthesis in *Catharanthus roseus* seedlings. *Acta Physiol. Plant* 39:267. 10.1007/s11738-017-2567-6

[B46] Cheng L. Pu L. Li A. Zhu X. Zhao P. Xu X. (2022). Implication of exogenous abscisic acid (ABA) application on phytoremediation: plants grown in co-contaminated soil. *Environ. Sci. Pollut. Res.* 29 8684–8693. 10.1007/s11356-021-16241-y 34491497

[B47] Choudhury F. K. Rivero R. M. Blumwald E. Mittler R. (2017). Reactive oxygen species, abiotic stress and stress combination. *Plant J.* 90 856–867. 10.1111/tpj.13299 27801967

[B48] Çoban Ö Baydar N. G. (2016). Brassinosteroid effects on some physical and biochemical properties and secondary metabolite accumulation in peppermint (*Mentha piperita* L.) under salt stress. *Ind. Crops Prod.* 86 251–258. 10.1016/j.indcrop.2016.03.049

[B49] Colebrook E. H. Thomas S. G. Phillips A. L. Hedden P. (2014). The role of gibberellin signalling in plant responses to abiotic stress. *J. Exp. Biol.* 217 67–75. 24353205 10.1242/jeb.089938

[B50] Colombo M. N. Francolini M. (2019). Glutamate at the vertebrate neuromuscular junction: from modulation to neurotransmission. *Cells* 8:996. 10.3390/cells8090996 31466388 PMC6770210

[B51] Cooper J. W. Hu Y. Beyyoudh L. Yildiz Dasgan H. Kunert K. Beveridge C. A. (2018). Strigolactones positively regulate chilling tolerance in pea and in *Arabidopsis*. *Plant Cell Environ.* 41 1298–1310. 10.1111/pce.13147 29341173

[B52] Cui M. Lin Y. Zu Y. Efferth T. Li D. Tang Z. (2015). Ethylene increases accumulation of compatible solutes and decreases oxidative stress to improve plant tolerance to water stress in *Arabidopsis*. *J. Plant Biol.* 58 193–201. 10.1007/s12374-014-0302-z

[B53] De Diego N. Saiz-Fernández I. Rodríguez J. L. Pérez-Alfocea P. Sampedro M. C. Barrio R. J. (2015). Metabolites and hormones are involved in the intraspecific variability of drought hardening in radiata pine. *J. Plant Physiol.* 188 64–71. 10.1016/j.jplph.2015.08.006 26433462

[B54] Decros G. Baldet P. Beauvoit B. Stevens R. Flandin A. Colombié S. (2019). Get the balance right: ROS homeostasis and redox signalling in fruit. *Front. Plant Sci.* 10:1091. 10.3389/fpls.2019.01091 31620143 PMC6760520

[B55] D’Haeze W. De Rycke R. Mathis R. Goormachtig S. Pagnotta S. Verplancke C. (2003). Reactive oxygen species and ethylene play a positive role in lateral root base nodulation of a semiaquatic legume. *PNAS* 100 11789–11794. 10.1073/pnas.1333899100 12975522 PMC208836

[B56] Di Sansebastiano G.-P. Fornaciari S. Barozzi F. Piro G. Arru L. (2014). New insights on plant cell elongation: a role for acetylcholine. *Int. J. Mol. Sci.* 15 4565–4582. 10.3390/ijms15034565 24642879 PMC3975414

[B57] Djanaguiraman M. Prasad P. Al-Khatib K. (2011). Ethylene perception inhibitor 1-MCP decreases oxidative damage of leaves through enhanced antioxidant defense mechanisms in soybean plants grown under high temperature stress. *Environ. Exp. Bot.* 71 215–223. 10.1016/j.envexpbot.2010.12.006

[B58] Djemal R. Khoudi H. (2022). The ethylene-responsive transcription factor of durum wheat, TdSHN1, confers cadmium, copper, and zinc tolerance to yeast and transgenic tobacco plants. *Protoplasma* 259 19–31. 10.1007/s00709-021-01635-z 33759027

[B59] Dos Santos Ribeiro D. G. da Silva B. R. S. da Silva Lobato A. K. (2019). Brassinosteroids induce tolerance to water deficit in soybean seedlings: contributions linked to root anatomy and antioxidant enzymes. *Acta Physiol. Plant* 41:82. 10.1007/s11738-019-2873-2

[B60] Du B. Zhao W. An Y. Li Y. Zhang X. Song L. (2019). Overexpression of an alfalfa glutathione *S*-transferase gene improved the saline-alkali tolerance of transgenic tobacco. *Biol. Open* 8:bio043505. 10.1242/bio.043505 31471294 PMC6777358

[B61] Erland L. A. E. Yasunaga A. Li I. T. S. Murch S. J. Saxena P. K. (2019). Direct visualization of location and uptake of applied melatonin and serotonin in living tissues and their redistribution in plants in response to thermal stress. *J. Pineal Res.* 66:e12527. 10.1111/jpi.12527 30267543

[B62] Fahad S. Sönmez O. Saud S. Wang D. Wu C. Adnan M. (eds) (2021a). *Plant growth regulators for climate-smart agriculture*, First Edn. Boca Raton, FL: CRC Press. 10.1201/9781003109013

[B63] Fahad S. Sonmez O. Saud S. Wang D. Wu C. Adnan M. (eds) (2021b). *Climate change and plants: biodiversity, growth and interactions.* Boca Raton: CRC Press. 10.1201/9781003108931

[B64] Fahad S. Sonmez O. Saud S. Wang D. Wu C. Adnan M. (eds) (2021c). *Developing climate resilient crops: improving global food security and safety.* CRC Press: Boca Raton. 10.1201/9781003109037

[B65] FAO (2017). *The Future of Food and Agriculture Trends and Challenges.* Rome: Food and Agriculture Organization of the United Nations.

[B66] Farooq M. S. Uzair M. Raza A. Habib M. Xu Y. Yousuf M. (2022). Uncovering the research gaps to alleviate the negative impacts of climate change on food security: a review. *Front. Plant Sci.* 13:927535. 10.3389/fpls.2022.927535 35903229 PMC9315450

[B67] Farooq O. Ali M. Sarwar N. Rehman A. Iqbal M. M. Naz T. (2021). Foliar applied brassica water extract improves the seedling development of wheat and chickpea. *Asian J. Agric. Biol.* 2021 1–7. 10.35495/ajab.2020.04.219

[B68] Fatima E.-S. Zakaria H. Khalid A. J. (2022). Improving copper stress tolerance in *Mentha suaveolens* L. by foliar application of salicylic acid. *Res. Sq.* [Preprint]. 10.21203/rs.3.rs-1339811/v1

[B69] Fatma M. Iqbal N. Gautam H. Sehar Z. Sofo A. D’Ippolito I. (2021). Ethylene and sulfur coordinately modulate the antioxidant system and ABA accumulation in mustard plants under salt stress. *Plants* 10:180. 10.3390/plants10010180 33478097 PMC7835815

[B70] Feller U. Vaseva I. I. (2014). Extreme climatic events: impacts of drought and high temperature on physiological processes in agronomically important plants. *Front. Environ. Sci.* 2:39. 10.3389/fenvs.2014.00039

[B71] Fichman Y. Mittler R. (2020). Rapid systemic signaling during abiotic and biotic stresses: Is the ROS wave master of all trades? *Plant J.* 102 887–896. 10.1111/tpj.14685 31943489

[B72] Fu J. Sun P. Luo Y. Zhou H. Gao J. Zhao D. (2019). Brassinosteroids enhance cold tolerance in *Elymus nutans* via mediating redox homeostasis and proline biosynthesis. *Environ. Exp. Bot.* 167:103831. 10.1016/j.envexpbot.2019.103831

[B73] Fu J. Wu Y. Miao Y. Xu Y. Zhao E. Wang J. (2017). Improved cold tolerance in *Elymus nutans* by exogenous application of melatonin may involve ABA-dependent and ABA-independent pathways. *Sci. Rep.* 7:39865. 10.1038/srep39865 28045095 PMC5206618

[B74] Fu J. Zhang S. Jiang H. Zhang X. Gao H. Yang P. (2022). Melatonin-induced cold and drought tolerance is regulated by brassinosteroids and hydrogen peroxide signaling in perennial ryegrass. *Environ. Exp. Bot.* 196:104815. 10.1016/j.envexpbot.2022.104815

[B75] Gao T. Zhang Z. Liu X. Wu Q. Chen Q. Liu Q. (2020a). Physiological and transcriptome analyses of the effects of exogenous dopamine on drought tolerance in apple. *Plant Physiol. Biochem.* 148 260–272. 10.1016/j.plaphy.2020.01.022 31982861

[B76] Gao Y. Li M. Zhang X. Yang Q. Huang B. (2020b). Up-regulation of lipid metabolism and glycine betaine synthesis are associated with choline-induced salt tolerance in halophytic seashore paspalum. *Plant Cell Environ.* 43 159–173. 10.1111/pce.13657 31600831

[B77] Gautam H. Fatma M. Sehar Z. Iqbal N. Albaqami M. Khan N. A. (2022). Exogenously-sourced ethylene positively modulates photosynthesis, carbohydrate metabolism, and antioxidant defense to enhance heat tolerance in rice. *Int. J. Mol. Sci.* 23:1031. 10.3390/ijms23031031 35162955 PMC8835467

[B78] Ghaffari H. Tadayon M. R. Nadeem M. Razmjoo J. Cheema M. (2020). Foliage applications of jasmonic acid modulate the antioxidant defense under water deficit growth in sugar beet. *Spanish J. Agric. Res.* 17:0805. 10.5424/sjar/2019174-15380

[B79] Ghassemi-Golezani K. Samea-Andabjadid S. (2022). Exogenous cytokinin and salicylic acid improve amino acid content and composition of faba bean seeds under salt stress. *Gesunde Pflanzen* [Preprint]. 10.1007/s10343-022-00673-8

[B80] Golfazani M. M. Taghvaei M. M. Samizadeh Lahiji H. Ashery S. Raza A. (2022). Investigation of proteins’ interaction network and the expression pattern of genes involved in the ABA biogenesis and antioxidant system under methanol spray in drought-stressed rapeseed. *3 Biotech* 12:217. 10.1007/s13205-022-03290-4 35965657 PMC9365922

[B81] Gomes B. R. Siqueira-Soares R. C. Dos Santos W. D. Marchiosi R. Soares A. R. Ferrarese-Filho O. (2014). The effects of dopamine on antioxidant enzymes activities and reactive oxygen species levels in soybean roots. *Plant Signal Behav.* 9:e977704. 10.4161/15592324.2014.977704 25482756 PMC4622826

[B82] Gondal M. R. Saleem M. Y. Rizvi S. A. Riaz A. Naseem W. Muhammad G. (2021). Assessment of drought tolerance in various cotton genotypes under simulated osmotic settings. *Asian J. Agric. Biol.* 2021:202008437. 10.35495/ajab.2020.08.437

[B83] Gong Q. Li Z. Wang L. Dai T. Kang Q. Niu D. (2020). Exogenous of Indole-3-Acetic Acid Application Alleviates Copper Toxicity in Spinach Seedlings by Enhancing Antioxidant Systems and Nitrogen Metabolism. *Toxics* 8:1. 10.3390/toxics8010001 31878158 PMC7151742

[B84] Guidotti B. B. Gomes B. R. Siqueira-Soares R. Soares A. R. FerrareseFilho O. (2013). The effects of dopamine on root growth and enzyme activity in soybean seedlings. *Plant Signal Behav.* 8:e25477. 10.4161/psb.25477 23838960 PMC4002588

[B85] Guo W. Chen R. Gong Z. Yin Y. Ahmed S. He Y. (2012). Exogenous abscisic acid increases antioxidant enzymes and related gene expression in pepper (*Capsicum annuum*) leaves subjected to chilling stress. *Genet. Mol. Res.* 11 4063–4080. 10.4238/2012.September.10.5 23079969

[B86] Gurmani A. R. Wang X. Rafique M. Jawad M. Khan A. R. Khan Q. U. (2022). Exogenous application of gibberellic acid and silicon to promote salinity tolerance in pea (*Pisum sativum* L.) through Na+ exclusion. *Saudi J. Biol. Sci.* 29:103305. 10.1016/j.sjbs.2022.103305 35602866 PMC9119841

[B87] Hafeez M. B. Zahra N. Zahra K. Raza A. Khan A. Shaukat K. (2021). Brassinosteroids: molecular and physiological responses in plant growth and abiotic stresses. *Plant Stress* 2:100029. 10.1016/j.stress.2021.100029

[B88] Haider S. Raza A. Iqbal J. Shaukat M. Mahmood T. (2022). Analyzing the regulatory role of heat shock transcription factors in plant heat stress tolerance: a brief appraisal. *Mol. Biol. Rep.* 49 5771–5785. 10.1007/s11033-022-07190-x 35182323

[B89] Hakla H. R. Sharma S. Urfan M. Yadav N. S. Rajput P. Kotwal D. (2021). Gibberellins target shoot-root growth, morpho-physiological and molecular pathways to induce cadmium tolerance in *Vigna radiata* L. *Agronomy* 11:896. 10.3390/agronomy11050896

[B90] Hasan M. M. Rahman M. A. Skalicky M. Alabdallah N. M. Waseem M. Jahan M. S. (2021). Ozone induced stomatal regulations, MAPK and phytohormone signaling in plants. *Int. J. Mol. Sci.* 22:6304. 10.3390/ijms22126304 34208343 PMC8231235

[B91] Hasanuzzaman M. Bhuyan M. H. M. B. Zulfiqar F. Raza A. Mohsin S. M. Mahmud J. A. (2020a). Reactive oxygen species and antioxidant defense in plants under abiotic stress: revisiting the crucial role of a universal defense regulator. *Antioxidants* 9:681. 10.3390/antiox9080681 32751256 PMC7465626

[B92] Hasanuzzaman M. Bhuyan M. H. M. B. Raza A. Hawrylak-Nowak B. Matraszek-Gawron R. Al Mahmud J. (2020b). Selenium in Plants: Boon or Bane? *Environ. Exp. Bot.* 178:104170. 10.1016/j.envexpbot.2020.104170

[B93] Hasanuzzaman M. Bhuyan M. H. Raza A. Hawrylak-Nowak B. Matraszek-Gawron R. Nahar K. (2020c). Selenium toxicity in plants and environment: biogeochemistry and remediation possibilities. *Plants* 9:1711. 10.3390/plants9121711 33291816 PMC7762096

[B94] Hasanuzzaman M. Matin M. Fardus J. Hasanuzzaman M. Hossain M. Parvin K. (2019). Foliar application of salicylic acid improves growth and yield attributes by upregulating the antioxidant defense system in *Brassica campestris* plants grown in lead-amended soils. *Acta Agrobot.* 72:1765. 10.5586/aa.1765

[B95] Hayashi K. Fujita Y. Ashizawa T. Suzuki F. Nagamura Y. Hayano-Saito Y. (2016). Serotonin attenuates biotic stress and leads to lesion browning caused by a hypersensitive response to *Magnaporthe oryzae* penetration in rice. *Plant J.* 85 46–45. 10.1111/tpj.13083 26603141

[B96] He H. Lei Y. Yi Z. Raza A. Zeng L. Yan L. (2021). Study on the mechanism of exogenous serotonin improving cold tolerance of rapeseed (*Brassica napus* L.) seedlings. *Plant Growth Regul.* 94 161–170. 10.1007/s10725-021-00700-0

[B97] He Y. Zhang T. Sun Y. Wang X. Cao Q. Fang Z. (2022). Exogenous IAA alleviates arsenic toxicity to rice and reduces arsenic accumulation in rice grains. *J. Plant Growth Regul.* 41 734–741. 10.1007/s00344-021-10336-z

[B98] Hedden P. (2018). Gibberellin biosynthesis in higher plants. *Ann. Plant Rev.* 49 37–71. 10.1002/9781119312994.apr0531

[B99] Heidari P. Entazari M. Ebrahimi A. Ahmadizadeh M. Vannozzi A. Palumbo F. (2021). Exogenous EBR ameliorates endogenous hormone contents in tomato species under low-temperature stress. *Horticulturaer* 7:84. 10.3390/horticulturae7040084

[B100] Hijaz F. Nehela Y. Killiny N. (2018). Application of gamma-aminobutyric acid increased the level of phytohormones in Citrus sinensis. *Planta* 248 909–918. 10.1007/s00425-018-2947-1 29961199

[B101] Hossain A. Raza A. Maitra S. Asaduzzaman M. Islam M. R. Hossain M. J. (2021). “Strigolactones: A Novel Carotenoid-Derived Phytohormone–Biosynthesis, Transporters, Signalling, and Mechanisms in Abiotic Stress,” in *Plant Growth Regulators: Signalling Under Stress Conditions*, eds Aftab T. Hakeem K. R. (Singapre: Springer), 275. 10.1007/978-3-030-61153-8_13

[B102] Hu X. Liu R. Li Y. Tai F. Xue R. Li C. (2010). Heat shock protein 70 regulates the abscisic acid-induced antioxidant response of maize to combined drought and heat stress. *Plant Growth Regul.* 60 225–235. 10.1007/s10725-009-9436-2

[B103] Husain T. Fatima A. Suhel M. Singh S. Sharma A. Prasad S. M. (2020). A brief appraisal of ethylene signaling under abiotic stress in plants. *Plant Signal Behav.* 15:1782051. 10.1080/15592324.2020.1782051 32692940 PMC8550184

[B104] Husen A. Iqbal M. Sohrab S. S. Ansari M. K. A. (2018). Salicylic acid alleviates salinity-caused damage to foliar functions, plant growth and antioxidant system in Ethiopian mustard (*Brassica carinata* A, Br.). *Agric. Food Sec.* 7:44. 10.1186/s40066-018-0194-0

[B105] Ibrahim O. H. Ali E. F. Eissa M. A. (2022). Jasmonic Acid and EDTA-enhanced Cd and Pb phytoextraction by the halophytic plants quail bush [Atriplex lentiformis (Torr.) S. Wats]. *J. Soil Sci. Plant Nutr.* 22 1434–1445. 10.1007/s42729-021-00743-2

[B106] Iftikhar A. Rizwan M. Adrees M. Ali S. ur Rehman M. Z. Qayyum M. F. (2020). Effect of gibberellic acid on growth, biomass, and antioxidant defense system of wheat (*Triticum aestivum* L.) under cerium oxide nanoparticle stress. *Environ. Sci. Pollut. Res.* 27 33809–33820. 10.1007/s11356-020-09661-9 32535824

[B107] Iqbal M. S. Zahoor M. Akbar M. Ahmad K. Hussain S. Munir S. (2022). Alleviating the deleterious effects of salt stress on wheat (*Triticum aestivum* L.) By foliar application of gibberellic acid and salicylic acid. *Appl. Ecol. Environ. Res.* 20 119–134. 10.15666/aeer/2001_119134

[B108] Irfan M. Kumar P. Ahmad I. Datta A. (2021). Unraveling the role of tomato Bcl-2-associated athanogene (BAG) proteins during abiotic stress response and fruit ripening. *Sci. Rep.* 11:21734. 10.1038/s41598-021-01185-7 34741097 PMC8571320

[B109] Isayenkov S. V. Maathuis F. J. (2019). Plant salinity stress: many unanswered questions remain. *Front. Plant Sci.* 10:80. 10.3389/fpls.2019.00080 30828339 PMC6384275

[B110] Islam M. R. Rahman M. M. Mohi-Ud-Din M. Akter M. Zaman E. Keya S. S. (2022). Cytokinin and gibberellic acid-mediated waterlogging tolerance of mungbean (Vigna radiata L. Wilczek). *PeerJ* 10:e12862. 10.7717/peerj.12862 35186468 PMC8820211

[B111] Jahan M. S. Shu S. Wang Y. Hasan M. M. El-Yazied A. A. Alabdallah N. M. (2021). Melatonin pretreatment confers heat tolerance and repression of heat-induced senescence in tomato through the modulation of ABA- and GA-mediated pathways. *Front. Plant Sci.* 12:650955. 10.3389/fpls.2021.650955 33841479 PMC8027311

[B112] Jahan M. S. Wang Y. Shu S. Zhong M. Chen Z. Wu J. Q. (2019). Exogenous salicylic acid increases the heat tolerance in tomato (*Solanum lycopersicum* L.) by enhancing photosynthesis efficiency and improving antioxidant defense system through scavenging of reactive oxygen species. *Sci. Hortic.* 247 421–429. 10.1016/j.scienta.2018.12.047

[B113] Jayakannan M. Bose J. Babourina O. Rengel Z. Shabala S. (2013). Salicylic acid improves salinity tolerance in Arabidopsis by restoring membrane potential and preventing salt-induced K^+^ loss via a GORK channel. *J. Exp. Bot.* 64 2255–2268. 10.1093/jxb/ert085 23580750 PMC3654417

[B114] Ji J. Yue J. Xie T. Chen W. Du C. Chang E. (2018). Roles of γ-aminobutyric acid on salinity-responsive genes at transcriptomic level in poplar: involving in abscisic acid and ethylene-signalling pathways. *Planta* 248 675–690. 10.1007/s00425-018-2915-9 29948123

[B115] Jiang L. Yang J. Liu C. Chen Z. Yao Z. Cao S. (2020). Overexpression of ethylene response factor ERF96 gene enhances selenium tolerance in *Arabidopsis*. *Plant Physiol. Biochem.* 149 294–300. 10.1016/j.plaphy.2020.02.024 32097848

[B116] Jiao C. Lan G. Sun Y. Wang G. Sun Y. (2021). Dopamine alleviates chilling stress in watermelon seedlings via modulation of proline content, antioxidant enzyme activity, and polyamine metabolism. *J. Plant Growth Regul.* 40 277–292. 10.1007/s00344-020-10096-2

[B117] Jiao X. Li Y. Zhang X. Liu C. Liang W. Li C. (2019). Exogenous dopamine application promotes alkali tolerance of apple seedlings. *Plants* 8:580. 10.3390/plants8120580 31817831 PMC6963653

[B118] Jin X. Liu T. Xu J. Gao Z. Hu X. (2019). Exogenous GABA enhances muskmelon tolerance to salinity-alkalinity stress by regulating redox balance and chlorophyll biosynthesis. *BMC Plant Biol.* 19:48. 10.1186/s12870-019-1660-y 30709373 PMC6359809

[B119] Jini D. Joseph B. (2017). Physiological mechanism of salicylic acid for alleviation of salt stress in rice. *Rice Sci.* 24 97–108. 10.1016/j.rsci.2016.07.007 35328712

[B120] Jumali S. S. Said I. M. Ismail I. Zainal Z. (2011). Genes induced by high concentration of salicylic acid in’*Mitragyna speciosa*’. *Aust. J. Crop Sci.* 5:296.

[B121] Kamran M. Danish M. Saleem M. H. Malik Z. Parveen A. Abbasi G. H. (2021a). Application of abscisic acid and 6-benzylaminopurine modulated morpho-physiological and antioxidative defense responses of tomato (*Solanum lycopersicum* L.) by minimizing cobalt uptake. *Chemosphere* 263:128169. 10.1016/j.chemosphere.2020.128169 33297138

[B122] Kamran M. Wang D. Alhaithloul H. A. S. Alghanem S. M. Aftab T. Xie K. (2021b). Jasmonic acid-mediated enhanced regulation of oxidative, glyoxalase defense system and reduced chromium uptake contributes to alleviation of chromium (VI) toxicity in choysum (*Brassica parachinensis* L.). *Ecotoxicol. Environ. Saf.* 208:111758. 10.1016/j.ecoenv.2020.111758 33396081

[B123] Kang K. Kim Y. S. Park S. Back K. (2009). Senescence-induced serotonin biosynthesis and its role in delaying senescence in rice leaves. *Plant Physiol.* 150 1380–1393. 10.1104/pp.109.138552 19439571 PMC2705024

[B124] Kaur H. Mukherjee S. Baluska F. Bhatla S. C. (2015). Regulatory roles of serotonin and melatonin in abiotic stress tolerance in plants. *Plant Signal Behav.* 10:e1049788. 10.1080/15592324.2015.1049788 26633566 PMC4883943

[B125] Kaur N. Kaur J. Grewal S. K. Singh I. (2019). Effect of heat stress on antioxidative defense system and its amelioration by heat acclimation and salicylic acid pre-treatments in three Pigeonpea Genotypes. *Ind. J. Agric. Biochem.* 32 106–110. 10.5958/0974-4479.2019.00014.5

[B126] Khalid A. Aftab F. (2020). Effect of exogenous application of IAA and GA3 on growth, protein content, and antioxidant enzymes of *Solanum tuberosum* L. grown in vitro under salt stress. *In Vitro Cell Dev. Biol. Plant* 56 377–389. 10.1007/s11627-019-10047-x

[B127] Khalil H. A. El-Ansary D. O. Ahmed Z. F. (2022). Mitigation of salinity stress on pomegranate (*Punica granatum* L. cv. Wonderful) plant using salicylic acid foliar spray. *Horticulturae* 8:375. 10.3390/horticulturae8050375

[B128] Khan A. Bilal S. Khan A. L. Imran M. Shahzad R. Al-Harrasi A. (2020). Silicon and Gibberellins: Synergistic Function in Harnessing ABA Signaling and Heat Stress Tolerance in Date Palm (*Phoenix dactylifera* L.). *Plants* 9:620. 10.3390/plants9050620 32413955 PMC7285242

[B129] Khan N. A. Khan M. I. R. Ferrante A. Poor P. (2017). Ethylene: a key regulatory molecule in plants. *Front. Plant Sci.* 8:1782. 10.3389/fpls.2017.01782 29085384 PMC5650606

[B130] Khedia J. Agarwal P. Agarwal P. K. (2019). Deciphering hydrogen peroxide-induced signalling towards stress tolerance in plants. *3 Biotech* 9:395. 10.1007/s13205-019-1924-0 31656733 PMC6789057

[B131] Khosravi-nejad F. Khavari-nejad R. A. Moradi F. Najafi F. (2022). Cytokinin and abscisic acid alleviate drought stress through changing organic acids profile, ion immolation, and fatty acid profile to improve yield of wheat (*Triticum aestivum* L.) cultivars. *Physiol. Mol. Biol. Plants* 28 1119–1129. 10.1007/s12298-022-01173-9 35722511 PMC9203616

[B132] Kisnieriene V. Ditchenko T. I. Kudryashov A. P. Sakalauskas V. Yurin V. M. Ruksenas O. (2012). The effect of acetylcholine on Characeae K^+^ channels at rest and during action potential generation. *Cent. Eur. J. Biol.* 7 1066–1075. 10.2478/s11535-012-0085-5

[B133] Kolupaev Y. E. Karpets Y. V. Yastreb T. Lugovaya A. (2018). Combined effect of salicylic acid and nitrogen oxide donor on stress-protective system of wheat plants under drought conditions. *Appl. Biochem. Microbiol.* 54 418–424. 10.1134/S0003683818040099

[B134] Ku Y.-S. Sintaha M. Cheung M.-Y. Lam H.-M. (2018). Plant hormone signaling crosstalks between biotic and abiotic stress responses. *Int. J. Mol. Sci.* 19:3206. 10.3390/ijms19103206 30336563 PMC6214094

[B135] Kumar G. Saadm K. R. Arya M. Puthusseri B. Mahadevappa P. Shetty N. P. (2021a). The Synergistic role of serotonin and melatonin during temperature stress in promoting cell division, ethylene and isoflavones biosynthesis in *Glycine max*. *Curr. Plant Biol.* 26:100206. 10.1016/j.cpb.2021.100206

[B136] Kumar G. Saad K. R. Puthusseri B. Arya M. Shetty N. P. Giridhar P. (2021b). Exogenous serotonin and melatonin regulate dietary isoflavones profoundly through ethylene biosynthesis in soybean [*Glycine max* (L.) Merr.]. *J. Agric. Food Chem.* 69 1888–1899. 10.1021/acs.jafc.0c07457 33529027

[B137] Kumar V. Irfan M. Ghosh S. Chakraborty N. Chakraborty S. Datta A. (2016). Fruit ripening mutants reveal cell metabolism and redox state during ripening. *Protoplasma* 253 581–594. 10.1007/s00709-015-0836-z 26008650

[B138] Kumari S. Kumar S. Prakash P. (2018). Exogenous application of cytokinin (6-BAP) ameliorates the adverse effect of combined drought and high temperature stress in wheat seedling. *J. Pharm. Phytochem.* 7 1176–1180.

[B139] Kwak J. M. Mori I. C. Pei Z.-M. Leonhardt N. Torres M. A. Dangl J. L. (2003). NADPH oxidase AtrbohD and AtrbohF genes function in ROS-dependent ABA signaling in Arabidopsis. *EMBO J.* 22 2623–2633. 10.1093/emboj/cdg277 12773379 PMC156772

[B140] Lang D. Yu X. Jia X. Li Z. Zhang X. (2020). Methyl jasmonate improves metabolism and growth of NaCl-stressed *Glycyrrhiza uralensis* seedlings. *Sci. Hortic.* 266 109287. 10.1016/j.scienta.2020.109287

[B141] Leister D. (2019). Piecing the puzzle together: the central role of reactive oxygen species and redox hubs in chloroplast retrograde signaling. *Antioxid. Redox Signal* 30 1206–1219. 10.1089/ars.2017.7392 29092621

[B142] Leng Y. Li Y. Ma Y. H. He L. F. Li S. W. (2021). Abscisic acid modulates differential physiological and biochemical responses of roots, stems, and leaves in mung bean seedlings to cadmium stress. *Environ. Sci. Pollut. Res.* 28 6030–6043. 10.1007/s11356-020-10843-8 32986195

[B143] Li C. Liu S. Yao X. Chen K. Zhang P. (2017a). PnF3H, a flavanone 3-hydroxylase from the Antarctic moss *Pohlia nutans*, confers tolerance to salt stress and ABA treatment in transgenic *Arabidopsis*. *Plant Growth Regul.* 83 489–500. 10.1007/s10725-017-0314-z

[B144] Li L. Lu X. Ma H. Lyu D. (2017b). Jasmonic acid regulates the ascorbate–glutathione cycle in *Malus baccata* Borkh. roots under low root-zone temperature. *Acta Physiol. Plant* 39 174. 10.1007/s11738-017-2469-7

[B145] Li J. Zhang M. Yang L. Mao X. Li J. Li L. (2021). OsADR3 increases drought stress tolerance by inducing antioxidant defense mechanisms and regulating OsGPX1 in rice (*Oryza sativa* L.). *Crop J.* 9 1003–1017. 10.1016/j.cj.2020.12.005

[B146] Li Q. Wang G. Guan C. Yang D. Wang Y. Zhang Y. (2019). Overexpression of *LcSABP*, an orthologous gene for salicylic acid binding protein 2, enhances drought stress tolerance in transgenic tobacco. *Front. Plant Sci.* 10:200. 10.3389/fpls.2019.00200 30847000 PMC6393331

[B147] Liang B. Gao T. Zhao Q. Ma C. Chen Q. Wei Z. (2018). Effects of exogenous dopamine on the uptake, transport, and resorption of apple ionome under moderate drought. *Front. Plant Sci.* 9:755. 10.3389/fpls.2018.00755 29922323 PMC5996283

[B148] Liang C. Meng Z. Meng Z. Malik W. Yan R. Lwin K. M. (2016). *GhABF2*, a bZIP transcription factor, confers drought and salinity tolerance in cotton (*Gossypium hirsutum* L.). *Sci. Rep.* 6:35040. 10.1038/srep35040 27713524 PMC5054369

[B149] Liang X. W. Zhang L. Natarajan S. K. Becker D. F. (2013). Proline mechanisms of stress survival. *Antioxid. Redox Sign.* 19 998–1011. 10.1089/ars.2012.5074 23581681 PMC3763223

[B150] Lima J. Lobato A. (2017). Brassinosteroids improve photosystem II efficiency, gas exchange, antioxidant enzymes and growth of cowpea plants exposed to water deficit. *Physiol. Mol. Biol. Plants* 23 59–72. 10.1007/s12298-016-0410-y 28250584 PMC5313414

[B151] Lin K.-H. Sei S.-C. Su Y.-H. Chiang C.-M. (2019). Overexpression of the Arabidopsis and winter squash superoxide dismutase genes enhances chilling tolerance via ABA-sensitive transcriptional regulation in transgenic *Arabidopsis*. *Plant Signal Behav.* 14:1685728. 10.1080/15592324.2019.1685728 31680612 PMC6866689

[B152] Liting W. Lina W. Yang Y. Pengfei W. Tiancai G. Guozhang K. (2015). Abscisic acid enhances tolerance of wheat seedlings to drought and regulates transcript levels of genes encoding ascorbate-glutathione biosynthesis. *Front. Plant Sci.* 6:458. 10.3389/fpls.2015.00458 26175737 PMC4485351

[B153] Liu H. Li C. Yan M. Zhao Z. Huang P. Wei L. (2022). Strigolactone is involved in nitric oxide-enhanced the salt resistance in tomato seedlings. *J. Plant Res.* 135 337–350. 10.1007/s10265-022-01371-2 35106650

[B154] Liu X. Wu D. Shan T. Xu S. Qin R. Li H. (2020a). The trihelix transcription factor OsGTγ-2 is involved adaption to salt stress in rice. *Plant Mol. Biol.* 103 545–560. 10.1007/s11103-020-01010-1 32504260

[B155] Liu Q. Gao T. Liu W. Liu Y. Zhao Y. Liu Y. (2020b). Functions of dopamine in plants: a review. *Plant Signal Behav.* 15:1827782. 10.1080/15592324.2020.1827782 33040671 PMC7671028

[B156] Liu Z. Ding Y. Wang F. Ye Y. Zhu C. (2016). Role of salicylic acid in resistance to cadmium stress in plants. *Plant Cell Rep.* 35 719–731. 10.1007/s00299-015-1925-3 26849671

[B157] Ma N. Hu C. Wan L. Hu Q. Xiong J. Zhang C. (2017). Strigolactones improve plant growth, photosynthesis, and alleviate oxidative stress under salinity in rapeseed (*Brassica napus* L.) by regulating gene expression. *Front. Plant Sci.* 8:1671. 10.3389/fpls.2017.01671 29021800 PMC5623956

[B158] Ma X. Zhan J. Huang B. (2016). Cytokinin-mitigation of salt-induced leaf senescence in perennial ryegrass involving the activation of antioxidant systems and ionic balance. *Environ. Exp. Bot.* 125 1–11. 10.1016/j.envexpbot.2016.01.002

[B159] Makawita G. I. P. S. Wickramasinghe I. Wijesekara I. (2021). Using brown seaweed as a biofertilizer in the crop management industry and assessing the nutrient upliftment of crops. *Asian J. Agric. Biol.* 1:257. 10.35495/ajab.2020.04.257

[B160] Márquez-López R. E. Quintana-Escobar A. O. Loyola-Vargas V. M. (2019). Cytokinins, the Cinderella of plant growth regulators. *Phytochem. Rev.* 18 1387–1408. 10.1007/s11101-019-09656-6

[B161] Medeiros D. B. Barros J. A. Fernie A. R. Araújo W. L. (2020). Eating away at ROS to regulate stomatal opening. *Trends Plant Sci.* 25 220–223. 10.1016/j.tplants.2019.12.023 31932167

[B162] Mekonnen D. W. Flügge U.-I. Ludewig F. (2016). Gamma-aminobutyric acid depletion affects stomata closure and drought tolerance of *Arabidopsis thaliana*. *Plant Sci.* 245 25–34. 10.1016/j.plantsci.2016.01.005 26940489

[B163] Merewitz E. B. Gianfagna T. Huang B. (2011). Photosynthesis, water use, and root viability under water stress as affected by expression of SAG12-ipt controlling cytokinin synthesis in *Agrostis stolonifera*. *J. Exp. Bot.* 62 383–395. 10.1093/jxb/erq285 20841349 PMC2993921

[B164] Miao Y. Lv D. Wang P. Wang X. C. Chen J. Miao C. (2006). An *Arabidopsis* glutathione peroxidase functions as both a redox transducer and a scavenger in abscisic acid and drought stress responses. *Plant Cell* 18 2749–2766. 10.1105/tpc.106.044230 16998070 PMC1626619

[B165] Milla M. A. R. Maurer A. Huete A. R. Gustafson J. P. (2003). Glutathione peroxidase genes in *Arabidopsis* are ubiquitous and regulated by abiotic stresses through diverse signaling pathways. *Plant J.* 36 602–615. 10.1046/j.1365-313X.2003.01901.x 14617062

[B166] Min Z. Li R. Chen L. Zhang Y. Li Z. Liu M. (2019). Alleviation of drought stress in grapevine by foliar-applied strigolactones. *Plant Physiol. Biochem.* 135 99–110. 10.1016/j.plaphy.2018.11.037 30529172

[B167] Mir R. A. Bhat B. A. Yousuf H. Islam S. T. Raza A. Rizvi M. A. (2022). Multidimensional Role of Silicon to Activate Resilient Plant Growth and to Mitigate Abiotic Stress. *Front. Plant Sci.* 13:819658. 10.3389/fpls.2022.819658 35401625 PMC8984490

[B168] Mittler R. (2017). ROS are good. *Trends Plant Sci.* 22 11–19. 10.1016/j.tplants.2016.08.002 27666517

[B169] Mittler R. Vanderauwera S. Gollery M. Van Breusegem F. (2004). Reactive oxygen gene network of plants. *Trends Plant Sci.* 9 490–498. 10.1016/j.tplants.2004.08.009 15465684

[B170] Mittler R. Zandalinas S. I. Fichman Y. Breusegem F. V. (2022). Reactive oxygen species signalling in plant stress responses. *Nat. Rev. Mol. Cell Biol.* [Epub ahead of print]. 10.1038/s41580-022-00499-2 35760900

[B171] Molassiotis A. Tanou G. Diamantidis G. (2010). NO says more than ‘YES’ to salt tolerance: salt priming and systemic nitric oxide signaling in plants. *Plant Signal Behav.* 5 209–212. 10.4161/psb.5.3.10738 20061805 PMC2881262

[B172] Moradi P. Vafaee Y. Mozafari A. A. Tahir N. A.-R. (2022). Silicon Nanoparticles and Methyl Jasmonate Improve Physiological Response and Increase Expression of Stress-related Genes in Strawberry cv. Paros Under Salinity Stress. *Silicon* [Preprint]. 10.1007/s12633-022-01791-8

[B173] Mubarik M. S. Khan S. H. Sajjad M. Raza A. Hafeez M. B. Yasmeen T. (2021). A manipulative interplay between positive and negative regulators of phytohormones: a way forward for improving drought tolerance in plants. *Physiol. Plant* 172 1269–1290. 10.1111/ppl.13325 33421147

[B174] Muhammad N. Hakim Quraishi U. Chaudhary H. J. Munis M. F. H. (2016). Indole-3-acetic acid induces biochemical and physiological changes in wheat under drought stress conditions. *Philipp. Agric. Sci.* 99 19–24. 27611241

[B175] Mukherjee S. (2018). Novel perspectives on the molecular crosstalk mechanisms of serotonin and melatonin in plants. *Plant Physiol. Biochem.* 132 33–45. 10.1016/j.plaphy.2018.08.031 30172851

[B176] Mukherjee S. David A. Yadav S. Baluška F. Bhatla S. C. (2014). Salt stress-induced seedling growth inhibition coincides with differential distribution of serotonin and melatonin in sunflower seedling roots and cotyledons. *Physiol. Plantarum* 152 714–728. 10.1111/ppl.12218 24799301

[B177] Mutlu S. Atıcı Ö Nalbantoðlu B. Mete E. (2016). Exogenous salicylic acid alleviates cold damage by regulating antioxidative system in two barley (*Hordeum vulgare* L.) cultivars. *Front. Life Sci.* 9:99–109. 10.1080/21553769.2015.1115430

[B178] Najafi-Kakavand S. Karimi N. Ghasempour H. R. (2019). Salicylic acid and jasmonic acid restrains nickel toxicity by ameliorating antioxidant defense system in shoots of metallicolous and non-metallicolous *Alyssum inflatum* Náyr. populations. *Plant Physiol. Biochem.* 135 450–459. 10.1016/j.plaphy.2018.11.015 30497973

[B179] Najafi-Kakavand S. Karimi N. Ghasempour H.-R. Raza A. Chaichi M. Modarresi M. (2022). Role of Jasmonic and Salicylic Acid on Enzymatic Changes in the Root of Two *Alyssum inflatum* Náyr. Populations Exposed to Nickel Toxicity. *J. Plant Growth Regul.* [Preprint]. 10.1007/s00344-022-10648-8

[B180] Naseer H. Shaukat K. Zahra N. Hafeez M. B. Raza A. Nizar M. (2022). Appraisal of foliar spray of iron and salicylic acid under artificial magnetism on morpho-physiological attributes of pea (*Pisum sativum* L.) plants. *PLoS One* 17:e0265654. 10.1371/journal.pone.0265654 35421099 PMC9009661

[B181] Naservafaei S. Sohrabi Y. Moradi P. Sweeney E. M. Mastinu A. (2021). Biological response of *Lallemantia iberica* to brassinolide treatment under different watering conditions. *Plants* 10:496. 10.3390/plants10030496 33807761 PMC8000778

[B182] Nawaz G. Han Y. Usman B. Liu F. Qin B. Li R. (2019). Knockout of *OsPRP1*, a gene encoding proline-rich protein, confers enhanced cold sensitivity in rice (*Oryza sativa* L.) at the seedling stage. *3 Biotech* 9 1–18. 10.1007/s13205-019-1787-4 31192079 PMC6555840

[B183] Nguyen H. C. Lin K. H. Ho S. L. Chiang C. M. Yang C. M. (2018). Enhancing the abiotic stress tolerance of plants: from chemical treatment to biotechnological approaches. *Physiol. Plan.* 164 452–466. 10.1111/ppl.12812 30054915

[B184] Nishiya Y. (2000). A Mutant sarcosine oxidase in which activity depends on flavin adenine dinucleotide Protein. *Exp. Purif.* 20 95–97. 10.1006/prep.2000.1299 11035956

[B185] Niu L. Liao W. (2016). Hydrogen peroxide signaling in plant development and abiotic responses: crosstalk with nitric oxide and calcium. *Front. Plant Sci.* 7:230. 10.3389/fpls.2016.00230 26973673 PMC4777889

[B186] Nizar M. Shaukat K. Zahra N. Hafeez M. B. Raza A. Samad A. (2022). Exogenous Application of Salicylic Acid and Hydrogen Peroxide Ameliorate Cadmium Stress in Milk Thistle by Enhancing Morpho-Physiological Attributes Grown at Two Different Altitudes. *Front. Plant Sci.* 12:809183. 10.3389/fpls.2021.809183 35154205 PMC8830505

[B187] Nolan T. M. Vukašinoviæ N. Liu D. Russinova E. Yin Y. (2020). Brassinosteroids: multidimensional regulators of plant growth, development, and stress responses. *Plant Cell* 32 295–318. 10.1105/tpc.19.00335 31776234 PMC7008487

[B188] Noor J. Ullah A. Saleem M. H. Tariq A. Ullah S. Waheed A. (2022). Effect of Jasmonic acid foliar spray on the morpho-physiological mechanism of salt stress tolerance in two soybean varieties (*Glycine max* L.). *Plants* 11:651. 10.3390/plants11050651 35270123 PMC8931774

[B189] O’Donnell P. J. Calvert C. Atzorn R. Wasternack C. Leyser H. M. O. Bowles D. J. (1996). Ethylene as a signal mediating the wound response of tomato plants. *Science* 274 1914–1917. 10.1126/science.274.5294.1914 8943205

[B190] Ostrowski M. Ciarkowska A. Jakubowska A. (2016). The auxin conjugate indole-3-acetyl-aspartate affects responses to cadmium and salt stress in *Pisum sativum* L. *J. Plant Physiol.* 191 63–72. 10.1016/j.jplph.2015.11.012 26717013

[B191] Paciolla C. Paradiso A. de Pinto M. (2016). “Cellular redox homeostasis as central modulator in plant stress response,” in *Redox State as a Central Regulator of Plant-Cell Stress Responses*, eds Gupta D. Palma J. Corpas F. (Berlin: Springer), 1–23. 10.1007/978-3-319-44081-1_1

[B192] Pakar N. Pirasteh-Anosheh H. Emam Y. Pessarakli M. (2016). Barley growth, yield, antioxidant enzymes, and ion accumulation affected by PGRs under salinity stress conditions. *J. Plant Nutr.* 39 1372–1379. 10.1080/01904167.2016.1143498

[B193] Panieri E. Santoro M. M. (2015). ROS signaling and redox biology in endothelial cells. *Cell Mol. Life Sci.* 72 3281–3303. 10.1007/s00018-015-1928-9 25972278 PMC11113497

[B194] Park W. J. (2011). Melatonin as an endogenous plant regulatory signal: debates and perspectives. *J. Plant Biol.* 54 143–149. 10.1007/s12374-011-9159-6

[B195] Pelagio-Flores R. Ortíz-Castro R. Méndez-Bravo A. Macías-Rodríguez L. López-Bucio J. (2011). Serotonin, a tryptophan-derived signal conserved in plants and animals, regulates root system architecture probably acting as a natural auxin inhibitor in *Arabidopsis thaliana*. *Plant Cell Physiol.* 52 490–508. 10.1093/pcp/pcr006 21252298

[B196] Pelagio-Flores R. Ruiz-Herrera L. F. López-Bucio J. (2016). Serotonin modulates Arabidopsis root growth via changes in reactive oxygen species and jasmonic acid–ethylene signaling. *Physiol. Plantarum* 158 92–105. 10.1111/ppl.12429 26864878

[B197] Per T. S. Fatma M. Asgher M. Javied S. Khan N. A. (2017). “Salicylic acid and nutrients interplay in abiotic stress tolerance,” in *Salicylic Acid: A Multifaceted Hormone*, eds Nazar R. Iqbal N. Khan N. (Berlin: Springer), 221–237. 10.1007/978-981-10-6068-7_11

[B198] Piotrowska-Niczyporuk A. Bajguz A. Zambrzycka-Szelewa E. Bralska M. (2018). Exogenously applied auxins and cytokinins ameliorate lead toxicity by inducing antioxidant defence system in green alga *Acutodesmus obliquus*. *Plant Physiol. Biochem.* 132 535–546. 10.1016/j.plaphy.2018.09.038 30316163

[B199] Podlešáková K. Ugena L. Spíchal L. Doležal K. De Diego N. (2019). Phytohormones and polyamines regulate plant stress responses by altering GABA pathway. *N. Biotechnol.* 48 53–65. 10.1016/j.nbt.2018.07.003 30048769

[B200] Pospíšilová H. Jiskrová E. Vojta P. Mrízová K. Kokáš F. Èudejková M. M. (2016). Transgenic barley overexpressing a cytokinin dehydrogenase gene shows greater tolerance to drought stress. *N. Biotechnol.* 33 692–705. 10.1016/j.nbt.2015.12.005 26773738

[B201] Postiglione A. E. Muday G. K. (2020). The Role of ROS Homeostasis in ABA-Induced Guard Cell Signaling. *Front. Plant Sci.* 11:968. 10.3389/fpls.2020.00968 32695131 PMC7338657

[B202] Pottosin I. Bonales-Alatorre E. Shabala S. (2014). Choline but not its derivative betaine blocks slow vacuolar channels in the halophyte *Chenopodium quinoa*: Implications for salinity stress responses. *FEBS Lett.* 588 3918–3923. 10.1016/j.febslet.2014.09.003 25240200

[B203] Qin C. Ahanger M. A. Zhou J. Ahmed N. Wei C. Yuan S. (2020). Beneficial role of acetylcholine in chlorophyll metabolism and photosynthetic gas exchange in *Nicotiana benthamiana* seedlings under salinity stress. *Plant Biol.* 22 357–365. 10.1111/plb.13079 31811780

[B204] Qin C. Su Y. Y. Li B. S. Cheng Y. Q. Wei C. C. Yuan S. (2019). Acetylcholine mechanism of action to enhance tolerance to salt stress in *Nicotiana benthamiana*. *Photosynthetica* 57 590–598. 10.32615/ps.2019.084

[B205] Qiu C. Zhang C. Wang N. Mao W. Wu F. (2021). Strigolactone GR24 improves cadmium tolerance by regulating cadmium uptake, nitric oxide signaling and antioxidant metabolism in barley (*Hordeum vulgare* L.). *Environ. Pollut.* 273:116486. 10.1016/j.envpol.2021.116486 33484996

[B206] Qiu Z. Guo J. Zhu A. Zhang L. Zhang M. (2014). Exogenous jasmonic acid can enhance tolerance of wheat seedlings to salt stress. *Ecotoxicol. Environ. Saf.* 104 202–208. 10.1016/j.ecoenv.2014.03.014 24726929

[B207] Raja V. Majeed U. Kang H. Andrabi K. I. John R. (2017). Abiotic stress: interplay between ROS, hormones and MAPKs. *Environ. Exp. Bot.* 137 142–157. 10.1016/j.envexpbot.2017.02.010

[B208] Rajabi Dehnavi A. Zahedi M. Ludwiczak A. Piernik A. (2022). Foliar Application of Salicylic Acid Improves Salt Tolerance of Sorghum (*Sorghum bicolor* (L.) Moench). *Plants* 11:368. 10.3390/plants11030368 35161349 PMC8839348

[B209] Ramakrishna A. Giridhar P. Ravishankar G. A. (2011). Phytoserotonin: a review. *Plant Signal. Behav.* 6 800–809. 10.4161/psb.6.6.15242 21617371 PMC3218476

[B210] Rather B. A. Mir I. R. Masood A. Anjum N. A. Khan N. A. (2022). Ethylene-nitrogen synergism induces tolerance to copper stress by modulating antioxidant system and nitrogen metabolism and improves photosynthetic capacity in mustard. *Environ. Sci. Pollut. Res.* 29 49029–49049. 10.1007/s11356-022-19380-y 35212900

[B211] Rattan A. Kapoor D. Kapoor N. Bhardwaj R. Sharma A. (2020). Brassinosteroids Regulate Functional Components of Antioxidative Defense System in Salt Stressed Maize Seedlings. *J. Plant Growth Regul.* 39 1465–1475. 10.1007/s00344-020-10097-1

[B212] Raza A. (2021). Eco-physiological and Biochemical Responses of Rapeseed (*Brassica napus* L.) to Abiotic Stresses: consequences and Mitigation Strategies. *J. Plant Growth Regul.* 40 1368–1388. 10.1007/s00344-020-10231-z

[B213] Raza A. (2022a). Metabolomics: A systems biology approach for enhancing heat stress tolerance in plants. *Plant Cell Rep.* 41 741–763. 10.1007/s00299-020-02635-8 33251564

[B214] Raza A. (2022b). Plant biotechnological tools: Solutions for raising climate-resilient crop plants. *Modern Phytomorphol.* 15 132–133.

[B215] Raza A. Ashraf F. Zou X. Zhang X. Tosif H. (2020). “Plant Adaptation and Tolerance to Environmental Stresses: Mechanisms and Perspectives,” in *Plant Ecophysiology and Adaptation under Climate Change: Mechanisms and Perspectives I*, ed Hasanuzzaman M. (Berlin: Springer), 117–145. 10.1007/978-981-15-2156-0_5

[B216] Raza A. Hussain S. Javed R. Hafeez M. B. Hasanuzzaman M. (2021a). “Antioxidant Defense Systems and Remediation of Metal Toxicity in Plants,” in *Approaches to the Remediation of Inorganic Pollutants*, ed. Hasanuzzaman M. (Singapore: Springer), 91–124. 10.1007/978-981-15-6221-1_6

[B217] Raza A. Tabassum J. Kudapa H. Varshney R. K. (2021b). Can omics deliver temperature resilient ready-to-grow crops? *Crit. Rev. Biotechnol.* 41 1209–1232. 10.1080/07388551.2021.1898332 33827346

[B218] Raza A. Habib M. Charagh S. Kakavand S. N. (2021d). “Genetic engineering of plants to tolerate toxic metals and metalloids,” In *Handbook of bioremediation*, eds Hasanuzzaman M. Prasad M. N. V. (Cambridge, MA: Academic Press), 411–436. 10.1016/b978-0-12-819382-2.00026-0

[B219] Raza A. Charagh S. Zahid Z. Mubarik M. S. Javed R. Siddiqui M. H. (2021c). Jasmonic acid: a key frontier in conferring abiotic stress tolerance in plants. *Plant Cell Rep.* 40 1513–1541. 10.1007/s00299-020-02614-z 33034676

[B220] Raza A. Razzaq A. Mehmood S. S. Zou X. Zhang X. Lv Y. (2019a). Impact of climate change on crops adaptation and strategies to tackle its outcome: a review. *Plants* 8:34. 10.3390/plants8020034 30704089 PMC6409995

[B221] Raza A. Mehmood S. S. Tabassum J. Batool R. (2019b). “Targeting plant hormones to develop abiotic stress resistance in wheat,” in *Wheat production in changing environments*, eds Hasanuzzaman M. Nahar K. Hossain M. (Berlin: Springer), 557–577. 10.1007/978-981-13-6883-7_22

[B222] Raza A. Tabassum J. Zahid Z. Charagh S. Bashir S. Barmukh R. (2022a). Advances in “Omics”. Approaches for Improving Toxic Metals/Metalloids Tolerance in Plants. *Front. Plant Sci.* 12:794373. 10.3389/fpls.2021.794373 35058954 PMC8764127

[B223] Raza A. Tabassum J. Mubarik M. S. Anwar S. Zahra N. Sharif Y. (2022b). Hydrogen sulfide: an emerging component against abiotic stress in plants. *Plant Biol.* 24 540–558. 10.1111/plb.13368 34870354

[B224] Raza A. Tabassum J. Fakhar A. Z. Sharif R. Chen H. Zhang Ch. (2022c). Smart reprograming of plants against salinity stress using modern biotechnological tools. *Crit. Rev. Biotechnol.* 10.1080/07388551.2022.2093695 [Epub ahead of print]. 35968922

[B225] Raza A. Charagh S. García-Caparrós P. Rahman M. A. Ogwugwa V. H. Saeed F. (2022d). Melatonin-mediated temperature stress tolerance in plants. *GM Crops Food*. 13, 196–217. 10.1080/21645698.2022.2106111 35983948 PMC9397135

[B226] Rehman A. Hassan F. Qamar R. Rehman A. U. (2021). Application of plant growth promoters on sugarcane (*Saccharum officinarum* L.) budchip under subtropical conditions. *Asian J. Agric. Biol.* 2021:202003202. 10.35495/ajab.2020.03.202

[B227] Riyazuddin R. Bela K. Horváth E. Rigó G. Gallé Á Szabados L. (2019). Overexpression of the *Arabidopsis* glutathione peroxidase-like 5 gene (*AtGPXL5*) resulted in altered plant development and redox status. *Environ. Exp. Bot.* 167:103849. 10.1016/j.envexpbot.2019.103849

[B228] Roychoudhury A. (2020). Neurotransmitter acetylcholine comes to the plant rescue. *J. Mol. Cell Biol. Forecast.* 3:1019.

[B229] Sabagh A. E. Mbarki S. Hossain A. Iqbal M. A. Islam M. S. Raza A. (2021). Potential role of plant growth regulators in administering crucial processes against abiotic stresses. *Front. Agron.* 3:648694. 10.3389/fagro.2021.648694

[B230] Saedipour S. (2016). Role of exogenous application of auxin on antioxidant enzyme activities in rice under salt stress. *J. Plant Physiol. Breed.* 6 49–61.

[B231] Saha I. Ghosh A. Dolui D. Fujita M. Hasanuzzaman M. Adak M. K. (2022). Differential Impact of Nitric Oxide and Abscisic Acid on the Cellular and Physiological Functioning of sub1A QTL Bearing Rice Genotype under Salt Stress. *Plants* 11:1084. 10.3390/plants11081084 35448812 PMC9029218

[B232] Sakamoto H. Matsuda O. Iba K. (2008). ITN1, a novel gene encoding an ankyrin-repeat protein that affects the ABA-mediated production of reactive oxygen species and is involved in salt-stress tolerance in *Arabidopsis thaliana*. *Plant J.* 56 411–422. 10.1111/j.1365-313X.2008.03614.x 18643991

[B233] Salim N. Raza A. (2020). Nutrient use efficiency (NUE) for sustainable wheat production: a review. *J. Plant Nutr.* 43 297–315. 10.1080/01904167.2019.1676907

[B234] Sallam A. Alqudah A. M. Dawood M. F. Baenziger P. S. Börner A. (2019). Drought stress tolerance in wheat and barley: advances in physiology, breeding and genetics research. *Int. J. Mol. Sci.* 20:3137. 10.3390/ijms20133137 31252573 PMC6651786

[B235] Samea-Andabjadid S. Ghassemi-Golezani K. Nasrollahzadeh S. Najafi N. (2018). Exogenous salicylic acid and cytokinin alter sugar accumulation, antioxidants and membrane stability of faba bean. *Acta Biol. Hungar.* 69 86–96. 10.1556/018.68.2018.1.7 29575914

[B236] Sattar A. Ul-Allah S. Ijaz M. Sher A. Butt M. Abbas T. (2022). Exogenous application of strigolactone alleviates drought stress in maize seedlings by regulating the physiological and antioxidants defense mechanisms. *Cereal Res. Commun.* 50 263–272. 10.1007/s42976-021-00171-z

[B237] Saxena S. C. Salvi P. Kamble N. U. Joshi P. K. Majee M. Arora S. (2020). Ectopic overexpression of cytosolic ascorbate peroxidase gene (*Apx1*) improves salinity stress tolerance in *Brassica juncea* by strengthening antioxidative defense mechanism. *Acta Physiol. Plant* 42 1–14. 10.1007/s11738-020-3032-5

[B238] Schieber M. Chandel N. S. (2014). ROS function in redox signaling and oxidative stress. *Curr. Biol.* 24 R453–R462. 10.1016/j.cub.2014.03.034 24845678 PMC4055301

[B239] Sedaghat M. Tahmasebi-Sarvestani Z. Emam Y. Mokhtassi-Bidgoli A. (2017). Physiological and antioxidant responses of winter wheat cultivars to strigolactone and salicylic acid in drought. *Plant Physiol. Biochem.* 119 59–69. 10.1016/j.plaphy.2017.08.015 28843889

[B240] Sehar Z. Iqbal N. Khan M. I. R. Masood A. Rehman MdT (2021). Ethylene reduces glucose sensitivity and reverses photosynthetic repression through optimization of glutathione production in salt-stressed wheat (*Triticum aestivum* L.). *Sci. Rep.* 11:12650. 10.1038/s41598-021-92086-2 34135422 PMC8209215

[B241] Shahzad K. Siddiqi E. H. Ahmad S. Zeb U. Muhammad I. Khan H. (2022). Exogenous application of indole-3-acetic acid to ameliorate salt induced harmful effects on four eggplants (Solanum melongena L.) varieties. *Sci. Horticult.* 292:110662. 10.1016/j.scienta.2021.110662

[B242] Sharif R. Su L. Chen X. Qi X. (2022). Hormonal interactions underlying parthenocarpic fruit formation in horticultural crops. *Horticult. Res.* 9:uhab024. 10.1093/hr/uhab024 35031797 PMC8788353

[B243] Sharif R. Xie C. Zhang H. Arnao M. Ali M. B. Ali Q. (2018). Melatonin and its effects on plant systems. *Molecules* 23:2352. 10.3390/molecules23092352 30223442 PMC6225270

[B244] Sharifi P. Bidabadi S. S. (2020). Strigolactone could enhances gas-exchange through augmented antioxidant defense system in *Salvia nemorosa* L. plants subjected to saline conditions stress. *Indus. Crops Prod.* 151:112460. 10.1016/j.indcrop.2020.112460

[B245] Sharma M. Irfan M. Kumar A. Kumar P. Datta A. (2021). Recent insights into plant circadian clock response against abiotic stress. *J. Plant Growth Regul.* 221:112403. 10.1007/s00344-021-10531-y

[B246] Sharma M. Kumar P. Verma V. Sharma R. Bhargava B. Irfan M. (2022a). Understanding plant stress memory response for abiotic stress resilience: molecular insights and prospects. *Plant Physiol. Biochem.* 179 10–24. 10.1016/j.plaphy.2022.03.004 35305363

[B247] Sharma A. Vishwakarma K. Singh N. K. Prakash V. Ramawat N. Prasad R. (2022b). Synergistic action of silicon nanoparticles and indole acetic acid in alleviation of chromium (CrVI) toxicity in *Oryza sativa* seedlings. *J. Biotechnol.* 343 71–82. 10.1016/j.jbiotec.2021.09.005 34534595

[B248] Shi H. Reiter R. J. Tan D.-X. Chan Z. (2015). Indole-3-acetic acid inducible 17 positively modulates natural leaf senescence through melatonin-mediated pathway in *Arabidopsis*. *J. Pineal Res.* 58 26–33. 10.1111/jpi.12188 25324183

[B249] Siddiqui M. H. Alamri S. Al-Khaishany Y. M. Khan N. M. Al-Amri A. Ali M. H. (2019). Exogenous melatonin counteracts NaCl induced damage by regulating the antioxidant system, proline and carbohydrates metabolism in tomato seedlings. *Int. J. Mol. Sci.* 20:353. 10.3390/ijms20020353 30654468 PMC6358940

[B250] Siddiqui M. H. Alamri S. Alsubaie Q. D. Ali H. M. (2020). Melatonin and gibberellic acid promote growth and chlorophyll biosynthesis by regulating antioxidant and methylglyoxal detoxification system in Tomato seedlings under salinity. *J. Plant Growth Regul.* 39 1488–1502. 10.1007/s00344-020-10122-3

[B251] Siddiqui M. H. Mukherjee S. Kumar R. Alansi S. Shah A. A. Kalaji H. M. (2022). Potassium and melatonin regulated—fructose-1, 6-bisphosphatase (FBPase) and sedoheptulose-1, 7-bisphosphatase (SBPase) activity improve photosynthetic efficiency, carbon assimilation and modulate glyoxylase system and tolerance to cadmium stress in tomato seedlings. *Plant Physiol. Biochem.* 171 49–65. 10.1016/j.plaphy.2021.12.018 34971955

[B252] Singh S. Kumar V. Kapoor D. Kumar S. Singh S. Dhanjal D. S. (2020). Revealing on hydrogen sulfide and nitric oxide signals coordination for plant growth under stress conditions. *Physiol. Plant* 168 301–317. 10.1111/ppl.13066 31264712

[B253] Sirhindi G. Mir M. A. Abd-Allah E. F. Ahmad P. Gucel S. (2016). Jasmonic acid modulates the physio-biochemical attributes, antioxidant enzyme activity, and gene expression in *Glycine max* under nickel toxicity. *Front. Plant Sci.* 7:591. 10.3389/fpls.2016.00591 27242811 PMC4864666

[B254] Soares A. R. MdeL F. Siqueira R. Böhm F. M. L. Z. Ferrarese-Filho O. (2007). L-DOPA increases lignification associated with *Glycine max* root growth-inhibition. *J. Chem. Ecol.* 33 265–275. 10.1007/s10886-006-9227-4 17195115

[B255] Soliman M. H. Alayafi A. A. El Kelish A. A. Abu-Elsaoud A. M. (2018). Acetylsalicylic acid enhance tolerance of *Phaseolus vulgaris* L. to chilling stress, improving photosynthesis, antioxidants and expression of cold stress responsive genes. *Bot. Stud.* 59:6. 10.1186/s40529-018-0222-1 29450670 PMC5814394

[B256] Srivastava D. Verma G. Chauhan A. S. Pande V. Chakrabarty D. (2019). Rice (*Oryza sativa* L.) tau class glutathione *S*-transferase (*OsGSTU30*) overexpression in *Arabidopsis thaliana* modulates a regulatory network leading to heavy metal and drought stress tolerance. *Metallomics* 11 375–389. 10.1039/C8MT00204E 30516767

[B257] Steffens B. (2014). The role of ethylene and ROS in salinity, heavy metal, and flooding responses in rice. *Front. Plant Sci.* 5:685. 10.3389/fpls.2014.00685 25538719 PMC4255495

[B258] Su L. Rahat S. Ren N. Kojima M. Takebayashi Y. Sakakibara H. (2021). Cytokinin and auxin modulate cucumber parthenocarpy fruit development. *Sci. Horticult.* 282:110026. 10.1016/j.scienta.2021.110026

[B259] Su P. Yan J. Li W. Wang L. Zhao J. Ma X. (2020). A member of wheat class III peroxidase gene family, TaPRX-2A, enhanced the tolerance of salt stress. *BMC Plant Biol.* 20:392. 10.1186/s12870-020-02602-1 32847515 PMC7449071

[B260] Su Y. Qin C. Begum N. Ashraf M. Zhang L. (2020). Acetylcholine ameliorates the adverse effects of cadmium stress through mediating growth, photosynthetic activity and subcellular distribution of cadmium in tobacco (*Nicotiana benthamiana*). *Ecotoxicol. Environ. Saf.* 198:110671. 10.1016/j.ecoenv.2020.110671 32344264

[B261] Sun C. Liu L. Wang L. Li B. Jin C. Lin X. (2021). Melatonin: a master regulator of plant development and stress responses. *J. Integr. Plant Biol.* 63 126–145. 10.1111/jipb.12993 32678945

[B262] Sun G. Mei Y. Deng D. Xiong L. Sun L. Zhang X. (2017). N-terminus-mediated degradation of ACS7 is negatively regulated by senescence signaling to allow optimal ethylene production during leaf development in *Arabidopsis*. *Front. Plant Sci.* 8:2066. 10.3389/fpls.2017.02066 29270180 PMC5723933

[B263] Suzuki N. Rivero R. M. Shulaev V. Blumwald E. Mittler R. (2014). Abiotic and biotic stress combinations. *New Phytol.* 203 32–43. 10.1111/nph.12797 24720847

[B264] Szechyńska-Hebda M. Skrzypek E. Da̧browska G. Biesaga-Kościelniak J. Filek M. Wêdzony M. (2007). The role of oxidative stress induced by growth regulators in the regeneration process of wheat. *Acta Physiol. Plantarum* 29 327–337. 10.1007/s11738-007-0042-5

[B265] Tan X. Long W. Zeng L. Ding X. Cheng Y. Zhang X. (2019). Melatonin-induced transcriptome variation of rapeseed seedlings under salt stress. *Int. J. Mol. Sci.* 20:5355. 10.3390/ijms20215355 31661818 PMC6862158

[B266] Tanveer M. (2019). Role of 24-Epibrassinolide in inducing thermo-tolerance in plants. *J. Plant Growth Regul.* 38 945–955. 10.1007/s00344-018-9904-x

[B267] Tanveer M. Ahmed H. A. I. (2020). *ROS signaling in modulating salinity stress tolerance in plants. Salt and Drought Stress Tolerance in Plants.* Cham: Springer, 299–314. 10.1007/978-3-030-40277-8_11

[B268] Tanveer M. Shabala S. (2020). “Neurotransmitters in signalling and adaptation to salinity stress in plants,” in *Neurotransmitters in Plant Signaling and Communication*, eds Baluška F. Mukherjee S. Ramakrishna A. (Cham: Springer), 49–73. 10.1007/978-3-030-54478-2_3

[B269] Tanveer M. Shahzad B. Sharma A. Khan E. A. (2019). 24-Epibrassinolide application in plants: an implication for improving drought stress tolerance in plants. *Plant Physiol. Biochem.* 135 295–303. 10.1016/j.plaphy.2018.12.013 30599306

[B270] Tanveer M. Yousaf U. (2020). “Plant single-cell biology and abiotic stress tolerance,” in *Plant Life under Changing Environment*, eds Tripathi D. K. Singh V. P. Chauhan D. K. Sharma S. Prasad S. M. Dubey N. K. (Amsterdam: Academic Press), 611–626. 10.1016/B978-0-12-818204-8.00026-6

[B271] Tayal R. Kumar V. Irfan M. (2022). Harnessing the power of hydrogen sulphide (H_2_S) for improving fruit quality traits. *Plant Biol.* 24 594–601. 10.1111/plb.13372 34866296

[B272] Thrane M. Paulsen P. V. Orcutt M. W. Krieger T. M. (2017). “Soy Protein: Impacts, Production, and Applications,” in *Sustainable Protein Sources*, Chap. 2, eds Nadathur S. R. Wanasundara J. P. D. Scanlin L. (San Diego, CA: Academic Press), 23–45. 10.1016/B978-0-12-802778-3.00002-0

[B273] Tiniola R. C. Pambid R. C. Bautista A. S. Dulay R. M. R. (2021). Light-emitting diode enhances the biomass yield and antioxidant activity of Philippine wild mushroom Lentinus swartzii. *Asian J. Agric. Biol.* 2021:202008435. 10.35495/ajab.2020.08.435

[B274] Trivedi D. K. Gill S. S. Tuteja N. (2016). *Abscisic acid (ABA): biosynthesis, regulation, and role in abiotic stress tolerance. Abiotic Stress Response in Plants.* Hoboken, NJ: Wiley, 311–322. 10.1002/9783527694570.ch15

[B275] Ullah I. Dawar K. Tariq M. Sharif M. Fahad S. Adnan M. (2022). Gibberellic acid and urease inhibitor optimize nitrogen uptake and yield of maize at varying nitrogen levels under changing climate. *Environ. Sci. Pollut. Res.* 29 6568–6577. 10.1007/s11356-021-16049-w 34455561

[B276] Voß U. Bishopp A. Farcot E. Bennett M. J. (2014). Modelling hormonal response and development. *Trends Plant Sci.* 19 311–319. 10.1016/j.tplants.2014.02.004 24630843 PMC4013931

[B277] Vukašinović N. Russinova E. (2018). BRexit: Possible brassinosteroid export and transport routes. *Trends Plant Sci.* 23 285–292. 10.1016/j.tplants.2018.01.005 29463443

[B278] Waadt R. Seller C. A. Hsu P. K. Takahashi Y. Munemasa S. Schroeder J. I. (2022). Plant hormone regulation of abiotic stress responses. *Nat. Rev. Mol. Cell Biol.* [Epub ahead of print]. 10.1038/s41580-022-00479-6 35513717 PMC9592120

[B279] Wang D. Yang Z. Wu M. Wang W. Wang Y. Nie S. (2022a). Enhanced brassinosteroid signaling via the overexpression of *SlBRI1* positively regulates the chilling stress tolerance of tomato. *Plant Sci.* 320:111281. 10.1016/j.plantsci.2022.111281 35643607

[B280] Wang T. Zhang D. Chen L. Wang J. Zhang W.-H. (2022b). Genome-wide analysis of the glutathione S-transferase family in wild *Medicago ruthenica* and drought-tolerant breeding application of *mrugstu39* gene in cultivated alfalfa. *Theor. Appl. Genet.* 135 853–864. 10.1007/s00122-021-04002-x 34817619

[B281] Wang F. Tan H. Zhang Y. Huang L. Bao H. Ding Y. (2021). Salicylic acid application alleviates cadmium accumulation in brown rice by modulating its shoot to grain translocation in rice. *Chemosphere* 263:128034. 10.1016/j.chemosphere.2020.128034 33297052

[B282] Wang L. Zhang X-L Wang L. Tian Y. Jia N. Chen S. (2017). Regulation of ethylene-responsive SlWRKY s involved in color change during tomato fruit ripening. *Sci. Rep.* 7:16674. 10.1038/s41598-017-16851-y 29192231 PMC5709409

[B283] Wang W.-R. Liang J.-H. Wang G.-F. Sun M.-X. Peng F.-T. Xiao Y.-S. (2020). Overexpression of *PpSnRK1α* in tomato enhanced salt tolerance by regulating ABA signaling pathway and reactive oxygen metabolism. *BMC Plant Biol.* 20:128. 10.1186/s12870-020-02342-2 32216751 PMC7099830

[B284] Wang Y. ShenShen W. Chan Z. Wu Y. (2015). Endogenous cytokinin overproduction modulates ROS homeostasis and decreases salt stress resistance in *Arabidopsis thaliana*. *Front. Plant Sci.* 6:1004. 10.3389/fpls.2015.01004 26635831 PMC4652137

[B285] Wang Y. Wan L. Zhang L. Zhang Z. Zhang H. Quan R. (2012). An ethylene response factor *OsWR1* responsive to drought stress transcriptionally activates wax synthesis related genes and increases wax production in rice. *Plant Mol. Biol.* 78 275–288. 10.1007/s11103-011-9861-2 22130861

[B286] Wang Y.-H. Zhang G. Chen Y. Gao J. Sun Y.-R. Sun M.-F. (2019). Exogenous application of gibberellic acid and ascorbic acid improved tolerance of okra seedlings to NaCl stress. *Acta Physiol. Plant* 41:93. 10.1007/s11738-019-2869-y

[B287] Wani S. H. Kumar V. Shriram V. Sah S. K. (2016). Phytohormones and their metabolic engineering for abiotic stress tolerance in crop plants. *Crop J.* 4 162–176. 10.1016/j.cj.2016.01.010

[B288] Wasaya A. Manzoor S. Yasir T. A. Sarwar N. Mubeen K. Ismail I. A. (2021). Evaluation of Fourteen Bread Wheat (*Triticum aestivum* L.) Genotypes by Observing Gas Exchange Parameters, Relative Water and Chlorophyll Content, and Yield Attributes under Drought Stress. *Sustainability* 3:4799. 10.3390/su13094799

[B289] Wasternack C. Strnad M. (2018). Jasmonates: news on occurrence, biosynthesis, metabolism and action of an ancient group of signaling compounds. *Int. J. Mol. Sci.* 19:2539. 10.3390/ijms19092539 30150593 PMC6164985

[B290] Wen D. Gong B. Sun S. Liu S. Wang X. Wei M. (2016). Promoting roles of melatonin in adventitious root development of *Solanum lycopersicum* L. by regulating auxin and nitric oxide signaling. *Front. Plant Sci.* 7:718. 10.3389/fpls.2016.00718 27252731 PMC4879336

[B291] Widrych A. Stachowiak J. Szopa J. (2004). The catecholamine potentates starch mobilization in transgenic potato tubers. *Plant Physiol. Biochem.* 42 103–109. 10.1016/j.plaphy.2003.11.002 15283125

[B292] Wu L. Zhang Z. Zhang H. Wang X.-C. Huang R. (2008). Transcriptional modulation of ethylene response factor protein JERF3 in the oxidative stress response enhances tolerance of tobacco seedlings to salt, drought, and freezing. *Plant Physiol.* 148 1953–1963. 10.1104/pp.108.126813 18945933 PMC2593663

[B293] Xia X. J. Fang P. P. Guo X. Qian XJ. Zhou J. Shi K. (2018). Brassinosteroid-mediated apoplastic H_2_O_2_-glutaredoxin 12/14 cascade regulates antioxidant capacity in response to chilling in tomato. *Plant Cell Environ.* 41 1052–1064. 10.1111/pce.13052 28776692

[B294] Xing X. Jiang H. Zhou Q. Xing H. Jiang H. Wang S. (2016). Improved drought tolerance by early IAA- and ABA-dependent H_2_O_2_ accumulation induced by α-naphthaleneacetic acid in soybean plants. *Plant Growth Regul.* 80 303–314. 10.1007/s10725-016-0167-x

[B295] Xu Z. Wang J. Zhen W. Sun T. Hu X. (2022a). Abscisic acid alleviates harmful effect of saline–alkaline stress on tomato seedlings. *Plant Physiol. Biochem.* 175 58–67. 10.1016/j.plaphy.2022.01.018 35180529

[B296] Xu X. Zhou J. Chen K. Wang Y. Ai Y. Zhang C. (2022b). Effect of indole-3-acetic acid supplementation on the physiology of Lolium perenne L. and microbial activity in cadmium-contaminated soil. *Environ. Sci. Pollut. Res.* 29 52483–52492. 10.1007/s11356-022-19417-2 35258728

[B297] Xu L. Chen H. Zhang T. Deng Y. Yan J. Wang L. (2022c). Salicylic acid improves the salt tolerance capacity of *Saponaria officinalis* by modulating its photosynthetic rate, osmoprotectants, antioxidant levels, and ion homeostasis. *Agronomy* 12:1443. 10.3390/agronomy12061443

[B298] Yamamoto K. Oguri S. Chiba S. Momonoki Y. S. (2009). Molecular cloning of acetyl-cholinesterase gene from *Salicornia europaea* L. *Plant Signal Behav.* 4 61–366. 10.4161/psb.4.5.8360 19816117 PMC2676743

[B299] Yan J. Li H. Li Y. Zhang N. Zhang S. (2022). Abscisic acid synthesis and root water uptake contribute to exogenous methyl jasmonate-induced improved tomato drought resistance. *Plant Biotechnol. Rep.* 16 183–193. 10.1007/s11816-022-00753-1

[B300] Yan M.-Y. Xie D.-L. Cao J.-J. Xia X.-J. Shi K. Zhou Y.-H. (2020). Brassinosteroid-mediated reactive oxygen species are essential for tapetum degradation and pollen fertility in tomato. *Plant J.* 102 931–994. 10.1111/tpj.14672 31908046

[B301] Yang Q.-Q. Zhao D.-S. Zhang C.-Q. Wu H.-Y. Li Q.-F. Gu M.-H. (2018). A connection between lysine and serotonin metabolism in rice endosperm. *Plant Physiol.* 176 1965–1980. 10.1104/pp.17.01283 29363563 PMC5841688

[B302] Yang W.-J. Du Y.-T. Zhou Y.-B. Chen J. Xu Z.-S. Ma Y.-Z. (2019). Overexpression of *TaCOMT* improves melatonin production and enhances drought tolerance in transgenic Arabidopsis. *Int. J. Mol. Sci.* 20:652. 10.3390/ijms20030652 30717398 PMC6387377

[B303] Yang Y. Qi M. Mei C. (2004). Endogenous salicylic acid protects rice plants from oxidative damage caused by aging as well as biotic and abiotic stress. *Plant J.* 40 909–919. 15584956 10.1111/j.1365-313X.2004.02267.x

[B304] Yao X. Ji J. Yue J. Shi S. Ou C. Jiang Z. (2020). Exogenous abscisic acid modulates reactive oxygen metabolism and related gene expression in *Platycladus orientalis* under H_2_O_2_-induced stress. *Russ. J. Plant Physiol.* 67 85–93. 10.1134/S1021443720010264

[B305] Zahra N. Hafeez M. B. Shaukat K. Wahid A. Hussain S. Naseer R. (2021). Hypoxia and Anoxia Stress: plant responses and tolerance mechanisms. *J. Agron. Crop Sci.* 207 249–284. 10.1111/jac.12471

[B306] Zayed M. El-Kafafi E. S. El Hafnawy S. F. El-Araby H. G. (2017). Effect of auxin treatment on growth and physiological traits in two sunflower cultivars under saline conditions. *J. Plant Prod.* 8 335–345. 10.21608/jpp.2017.39633

[B307] Zhang C. He Q. Wang M. Gao X. Chen J. Shen C. (2020). Exogenous indole acetic acid alleviates Cd toxicity in tea (*Camellia sinensis*). *Ecotoxicol. Environ. Saf.* 190:110090. 10.1016/j.ecoenv.2019.110090 31874405

[B308] Zhang J. Shi Y. Zhang X. Du H. Xu B. Huang B. (2017). Melatonin suppression of heat-induced leaf senescence involves changes in abscisic acid and cytokinin biosynthesis and signaling pathways in perennial ryegrass (*Lolium perenne* L.). *Environ. Exp. Bot.* 138 36–45. 10.1016/j.envexpbot.2017.02.012

[B309] Zhang L. Wu M. Teng Y. Jia S. Yu D. Wei T. (2019). Overexpression of the glutathione peroxidase 5 (*RcGPX5*) gene from *Rhodiola crenulata* increases drought tolerance in *Salvia miltiorrhiza*. *Front. Plant Sci.* 9:1950. 10.3389/fpls.2018.01950 30687353 PMC6333746

[B310] Zhang M. Smith J. A. C. Harberd N. P. Jiang C. (2016). The regulatory roles of ethylene and reactive oxygen species (ROS) in plant salt stress responses. *Plant Mol. Biol.* 91 651–659. 10.1007/s11103-016-0488-1 27233644

[B311] Zhang X. Zhang L. Ma C. Su M. Wang J. Zheng S. (2022). Exogenous strigolactones alleviate the photosynthetic inhibition and oxidative damage of cucumber seedlings under salt stress. *Sci. Hortic.* 297:110962. 10.1016/j.scienta.2022.110962

[B312] Zhang Y. P. Zhu X. H. Ding H. D. Yang S. J. Chen Y. Y. (2013). Foliar application of 24-epibrassinolide alleviates high-temperature induced inhibition of photosynthesis in seedlings of two melon cultivars. *Photosynthetica* 51 341–349. 10.1007/s11099-013-0031-4

[B313] Zheng X. Li Y. Xi X. Ma C. Sun Z. Yang X. (2021). Exogenous Strigolactones alleviate KCl stress by regulating photosynthesis, ROS migration and ion transport in *Malus hupehensis* Rehd. *Plant Physiol. Biochem.* 159 113–122. 10.1016/j.plaphy.2020.12.015 33359960

[B314] Zhou X. Tan Z. Zhou Y. Guo S. Sang T. Wang Y. (2022). Physiological mechanism of strigolactone enhancing tolerance to low light stress in cucumber seedlings. *BMC Plant Biol.* 22:30. 10.1186/s12870-021-03414-7 35027005 PMC8756728

[B315] Zulfiqar F. Chen J. Finnegan P. M. Younis A. Nafees M. Zorrig W. (2021). Application of trehalose and salicylic acid mitigates drought stress in sweet basil and improves plant growth. *Plants* 10:1078. 10.3390/plants10061078 34072096 PMC8230182

[B316] Zwanenburg B. Blanco-Ania D. (2018). Strigolactones: new plant hormones in the spotlight. *J. Exp. Bot.* 69 2205–2218. 10.1093/jxb/erx487 29385517

